# Influence of elastomeric and steel ligatures on periodontal health during fixed appliance orthodontic treatment: a systematic review and meta-analysis

**DOI:** 10.1186/s40510-024-00520-8

**Published:** 2024-06-17

**Authors:** Umar Hussain, Alessandra Campobasso, Muhammad Noman, Shamsul Alam, Rida Mujeeb, Sofia Shehzad, Spyridon N. Papageorgiou

**Affiliations:** 1 Saidu College of Dentistry, Swat, Pakistan; 2 Department of Clinical and Experimental Medicine, University of Foggia, Foggia, Italy; 3 Sharif Medical and Dental College , Lahore, Pakistan; 4 Health Department Khyber Pakhtunkhwa, Peshawar, Pakistan; 5 Fatima Memorial Hospital, Lahore, Pakistan; 6 Community Dentistry, Sardar Begum Dental College, Gandhara University, Peshawar, Pakistan; 7 Clinic of Orthodontics and Pediatric Dentistry, Center for Dental Medicine, University of Zurich, Plattenstrasse 11, Zurich, 8032 Switzerland

**Keywords:** Orthodontics, Fixed appliances, Periodontal index, *Streptococcus mutans*, Clinical trials, Systematic review, Meta-analysis

## Abstract

**Introduction:**

Metallic and elastomeric ligatures are widely used in orthodontics to secure the archwire within the bracket slots, but elastomeric ligatures have traditionally been associated with increased microbial colonization, which could adversely affect periodontal health.

**Aim:**

This systematic review compares the periodontal effects of elastomeric and steel ligatures used for orthodontic fixed appliances.

**Methods:**

Unrestricted literature search of 7 databases (MEDLINE, Scopus, Web of Science, Embase, Cochrane Database of Systematic Reviews, Cochrane Central Register of Controlled Trials, and Virtual Health Library) up to July 2023 were performed for randomized / non-randomized clinical studies on humans comparing the two ligation methods during fixed-appliance therapy. After duplicate study selection, data extraction, and risk-of-bias assessment with the Risk of Bias (RoB) 2 or the Risk Of Bias In Non-randomized Studies - of Interventions (ROBINS-I) tool, random-effects meta-analyses of Mean Differences (MD) or Standardized Mean Differences (SMD) and their 95% confidence intervals (CIs) were carried out, followed by assessment of certainty of existing evidence with the Grades of Recommendation, Assessment, Development, and Evaluation (GRADE) approach.

**Results:**

A total of 11 studies (3 randomized / 8 non-randomized) with 354 patients (mean age 14.7 years and 42% male) were included. No statistically significant differences were seen for plaque index (5 studies; SMD = 0.48; 95% CI = -0.03 to 1.00; *P* = 0.07), gingival index (2 studies; MD = 0.01; 95% CI = -0.14 to 0.16; *P* = 0.89), probing pocket depth (2 studies; MD = 0; 95% CI = -0.17 to 0.16; *P* = 0.97), or Streptococcus mutans counts (4 studies; SMD = 0.40; 95% CI=-0.41 to 1.20; P = 0.21). Elastomeric ligatures were associated with moderately increased total bacterial load (3 studies; SMD = 0.43; 95% CI = 0.10 to 0.76; *P* = 0.03). Confidence in these estimates was low in all instances due to the inclusion of non-randomized studies with high risk of bias.

**Conclusions:**

Existing low quality evidence indicates that ligature method does not seem to influence the periodontal health during fixed treatment, even if elastomeric ligatures are associated with a moderate increase of bacterial load.

**Registration:**

PROSPERO (CRD42023444383)

**Supplementary Information:**

The online version contains supplementary material available at 10.1186/s40510-024-00520-8.

## Introduction

### Rationale

About every third (35.4%) child aged 8–15 has some kind of malocclusion [1], which can negatively influence quality of life, even to a greater extent than caries [2]. Despite the recent popularity of orthodontic aligners [3], conventional fixed appliance still remains the therapeutic gold standard [4].

The typical orthodontic fixed appliance involves the use of brackets bonded to the tooth’s labial or lingual surface that present complex morphology and favor biofilm retention [5]. Brackets, bands, archwires, and ligatures can make routine oral hygiene challenging [6] by increasing biofilm accumulation and decreasing the physiological self-cleaning action of the saliva and the tongue [5].

Fixed appliance treatment is associated with increased bacterial accumulation, which may alter the oral ecosystem towards pathogenic colonization [7, 8], and increases the risk of caries, periodontal inflammation, and enamel demineralization [6, 9].

Ligation of the archwire within the bracket slot is achieved either with metallic stainless steel ligature wires or elastomeric modules (in the form of single ‘o rings’ or elastic chains of multiple rings), which present substantial differences in their bacterial colonization [11–13]. In clinical practice, ligature choice is based on patient preference, esthetic demands, logistic reasons related to appliance interval, or differential clinical performance due the materials’ own characteristics [13]. Elastomeric ligatures have a porous and rough surface as they are composed of organic material, while steel ligatures are made of inorganic metal material, ensuring a smooth and inert surface [14]. During intraoral use, elastomeric ligatures show considerable adsorption and the progressive formation of a proteinaceous biofilm that undergoes partial calcification [15]. Therefore, the use of elastomeric ligatures has been suggested to promote bacterial retention and have a more negative effect on oral hygiene than their metallic counterparts [12–16].

Previous studies have provided conflicting evidence on the effects of ligature materials on periodontal health. A recent systematic review [17] on the subject reported that currently no recommendations for one ligation mode over the other are possible and that stainless steel ligatures might be better for biofilm management. However, in that review only two databases were searched up to 2021 and no quantitative data synthesis (meta-analysis) was performed. Another systematic review found that fixed appliances ligated with steel ligatures are associated with increased plaque index scores than self-ligating fixed appliances that have no ligature [18] but did not compare them to elastomeric ligatures.

### Objective

The primary aim of this systematic review was to compare the periodontal effects of orthodontic fixed appliances ligated with either elastomeric or stainless steel ligatures.

## Materials and methods

### Protocol and registration

This systematic review was carried out in adherence to the Cochrane Handbook [19] and its report follows the Preferred Reporting Items for Systematic reviews and Meta-Analyses (PRISMA) 2020 statement [20]. The study’s protocol was developed a priori and was pre-registered in PROSPERO (CRD42023444383), while any protocol deviations were openly disclosed for transparency reasons (Appendix 1).

### Eligibility criteria

Included were studies on patients undergoing orthodontic treatment with fixed labial appliances that are ligated either with elastomeric or stainless steel ligatures. The primary outcome was Pocket Probing Depth (PPD), while secondary outcomes included Plaque Index (PI), Gingival Bleeding Index (GBI) or Gingival Index (GI), total bacterial count, and Streptococcus mutans counts. Included were comparative clinical studies (both randomized and nonrandomized) on humans, while excluded were studies on patients diagnosed with periodontal disease, antibiotic use in the last six months or systemic disease, case reports, case series, animal studies, in vitro/in situ/ex vivo studies, and non-clinical studies.

### Information sources and search

Two authors (UH, AC) independently conducted a search of seven databases (MEDLINE via PubMed, Scopus, Web of Science, Embase, Cochrane Database of Systematic Reviews, Cochrane Central Register of Controlled Trials, and Virtual Health Library), using appropriate search terms (Appendix 2), without any restrictions for publication year, language, or type. Furthermore, the reference lists of eligible articles and existing systematic reviews were manually reviewed to identify any potentially relevant studies that might have been missed from the systematic search. Finally, all included studies were checked in Google Scholar using the “Related Articles” option to identify any additional studies.

### Selection process

The results of the literature search were imported in Endnote X9 software (Clarivate, Philadelphia, PA) for deduplication and then transferred to electronic spreadsheets. At first, the titles and/or abstracts of all studies identified in the literature were screened and then the remaining full texts were evaluated against the eligibility criteria. Study selection was conducted independently by two authors (UH, SS) and any disagreements were resolved through discussion with a third author (SA).

### Data collection process and items

Data collection utilized a pre-defined and piloted extraction form, encompassing the following data: (a) study characteristics, including the primary author with the year of publication, study design, and clinical setting (country); (b) patient characteristics, comprising age and sex; (c) sample size for each intervention; (d) follow-up duration; and (e) measured outcomes. To ensure accuracy and uniformity, two authors (UH, MN) independently performed the data extraction, while any disparities were resolved through discussion with a third author (SS).

### Risk of bias of individual studies

The risk of bias of randomized trials was assessed with the Cochrane Risk of Bias (ROB) 2 tool [21] on an intention to treat basis. The risk of bias of non-randomized comparative studies was assessed with the Risk Of Bias In Nonrandomized Studies of Interventions (ROBINS-I) tool [22]. All assessments were conducted by two authors independently (UH, RM), with discrepancies resolved through discussion with a third author (SS).

### Effect measures and synthesis measures

The Mean Difference (MD) with its 95% Confidence Interval (CI) was chosen for same outcomes used across studies, while the Standardized Mean Difference (SMD) was used when variations of indices measuring the same outcome (like different PIs) were used. As the periodontal effects of different ligatures were expected to vary among studies (according to different elastomeric materials, level of oral hygiene, and position in mouth of the teeth being measured) a random-effects model was deemed a priori more appropriate to capture this variability and calculate the average distribution of treatment effects across studies [23] and a novel restricted maximum likelihood variance estimator was chosen due to improved performance [24]. Between-study heterogeneity was gauged through forest plot inspection, tau^2^ (absolute heterogeneity), I^2^ (relative inconsistency), and uncertainty intervals for all heterogeneity estimates (while also evaluating localization of heterogeneity in the forest plot and existing uncertainty). 95% predictions were calculated to incorporate existing heterogeneity and aid in meta-analytical interpretation by providing a range of possible future effects across the various clinical settings [25]. All analyses were conducted in R 4.2.2. (R Foundation for Statistical Computing, Vienna, Austria) by one person (SNP), with open data provision [26], two-sided P-values, and alpha = 5% (Appendix 1).

### Reporting bias assessment and certainty assessment

Hints of reporting biases (including the possibility of publication bias) were planned (Appendix 1) but could ultimately not be assessed. To gauge the certainty of the meta-analytic results, the Grades of Recommendations, Assessment, Development, and Evaluation (GRADE) approach was employed [27] and findings were summarized using a revised table format [28].

## Results

### Study selection

**Fig. 1:**
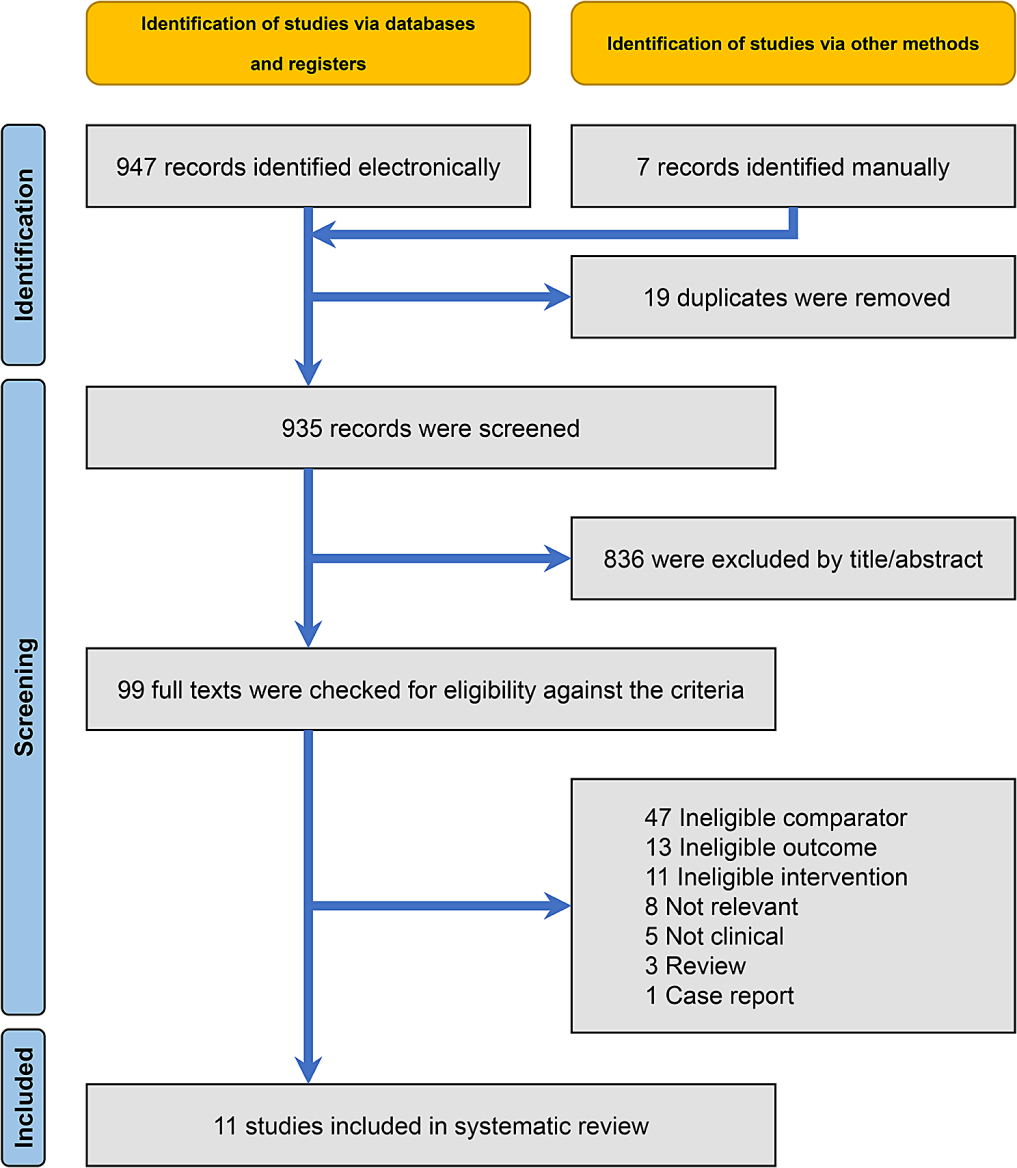
Flowdiagram for the identification and selection of studies for this review

The initial electronic database search yielded 947 records and seven additional were identified through manual searching (Fig. 1). After eliminating 19 duplicates, 935 records were left for further evaluation and were assessed against the eligibility criteria (Appendix 3). Ultimately, 11 publications, corresponding to 11 distinct clinical studies, were included in the quantitative and qualitative synthesis.

### Study characteristics

Table 1 shows the characteristics of the 11 studies included in this analysis. Among these studies, the majority (82%; 9/11) were of within-person (clustered) design where both ligation methods were used on different teeth and only 27% (3/11) were randomized trials. These studies were conducted in university clinics of six different countries (Brazil, India, Italy, Pakistan, Sweden, and Turkey). In total 354 patients were included in the 11 studies (median 21 patients per study), who were 42% male (127/301; from the 9 studies reporting sex) and were on average 14.7 years of age (from the 5 studies reporting on age). The majority of included studies (91%; 10/11) used elastomeric ligatures, one study (9%) used elastic chains, and two studies also compared different kinds of elastomeric ligatures. Among the included studies, plaque index was measured in seven studies (64%), gingival index in four studies (36%), probing pocket depth in three studies (27%), bacterial counts in two studies (18%), and Streptococcus mutans counts in five studies (45%).

**Table 1:** Characteristics of the included studies

Study	Design; Setting; country	Participants (M/F); age†	EL type	Outcomes/Related Indices	Observation (weeks)
Bretas 2005	RCT_WP_; Uni; BRA	EL/SS: 23 (NR); NR*	NR	Sm count (saliva / biofilm)	2.1, 4.3
Condo 2012	pNRS_WP_; Uni; ITA	EL/SS: 40 (20/20); NR	EL1: LTX (Leone Spa, Sesto Fiorentino, FI, Italy) EL2: PU (Micerium Spa, Avegno, Ge, Italy),) EL3: PU low friction (Leone Spa, Sesto, Fiorentino, FI, Italy	Plaque retention score	4.0
Dagdeviren 2021	pNRS_WP/CO_; Uni; TUR	EL/SS: 10 (6/4); 13.6	EL1: PU (Slide Low-Friction; Leone, Firenze, Italy) EL2: NR (Tough-O Energy; Rocky Mountain Orthodontics, DEN, USA) EL3: NR (Sili Ties; Dentsply Sirona, Surrey KT13 0NY, UK)	PI_SiLo_; GI_LoSi_; Sm count (biofilm); surface roughness	6.0, 10.0, 14.0, 18.0
de Souza 2008	pNRS_WP_; Uni; BRA	EL/SS: 14 (6/8); 17.0	NR	PI_SiLo_; GBI_AiBa_; PPD; PCR	25.7
Fosberg 1991	RCT_WP/CO_; Uni; SWE	EL/SS: 12 (6/6); NR	NR	Bacterial load (biofilm); Sm count (saliva); AeLa / AnLa count (saliva)	10.0, 19.0, 34.0, 61.0
Islam 2014	rNRS_PAR_; Uni; PAK	EL/SS: 131 (48/83); NR	NR	PI_SiLo_	≥ 4.3
Rodrigues 2011	rNRS_WP/CO_; Uni; BRA	EL/SS: 20 (9/11); 13.5	NR	PI_SiLo_; GBI_AiBa_; PPD	25.7
Savant 2016	pNRS_WP_; Uni; IND	EL/SS: 30 (NR); NR	NR	PI_QH;_ BBI	4.3
Shirozaki 2017	RCT_WP_; Uni; BRA	EC/SL: 13 (5/8); 13.8	EC: NR (Morelli, Sorocaba, SP, Brazil)	Sm count (biofilm)	1.0
Thenarasu 2018	pNRS_PAR_; Uni; IND	EL/SS: 40 (18/22); NR	NR	PI_SiLo_	≥ 4.3
Turkkahraman 2005	pNRS_WP_; Uni; TUR	EL/SS: 21 (9/12); 15.4	NR	PI_SiLo_; GI_LoSi_; PPD; bacterial count (biofilm); Sm count (saliva); AeLa / AnLa count (saliva	1.0, 5.0

*Aa* Aggregatibacter actinomycetemcomitans; *AeLa* anaerobic lactobacilli; *AiBa* Ainamo & Bay index; *AnLa* anaerobic lactobacilli; *BBI* bracket bond index; *CO* cross-over design; *EC* elastic chain; *EL* elastomeric ligature group; *GBI* gingival bleeding index; *GI* gingival index; *LoSi* Löe & Silness index; *LTX* latex; *NR* not reported pNRS, prospective non-randomized study; *PAR* parallel design; *PCR* polymerase chain reaction for bacterial identification; *Pg* Porphyromonas gingivalis; *PI* plaque index; *Pi* Prevotella intermedia; *Pn* Prevotella nigrescens; *pNRS* prospective non-randomized study; *PPD* pocket probing depth; *PU* polyurethane; *QH* Quigley Hein index; *RCT* randomized clinical trial; *rNRS* retrospective non-randomized study; *SiLo* Silness & Löe index; *SL* stainless steel long-tie; *Sm* Streptococcus mutans; *SS* stainless steel ligature; *Tf* Tannerella forsythia; *WP* within-person design

* included only half of the study sample, that didn’t use 0.4% stannous fluoride gel

### Risk of bias in studies

Three randomized controlled trials were evaluated using the ROB 2 tool, and all were found to have a high risk of bias due to randomization issues, deviations from intended interventions, and outcome measurement issues (Table 2a). Of the eight non-randomized studies evaluated using ROBINS-I, all were deemed to be in serious risk of bias, primarily attributed to confounding and participant selection. Additionally, a moderate risk of bias was identified in these studies concerning the classification of interventions and the risk of missing data (Table 2b).

**Table 2a:** Risk of bias assessment of included randomized trials with the ROB 2 tool

Study	Randomization process	Deviations from the intended interventions	Missing outcome data	Measurement of the outcome	Selection of the reported result	Overall
Bretas 2005	High risk	High risk	Low risk	Low risk	Some concerns	High risk
Fosberg 1991	High risk	High risk	Low risk	Low risk	Some concerns	High risk
Shirozaki 2017	Some concerns	High risk	Low risk	High risk	Some concerns	High risk

**Table 2b:** Risk of bias assessment of included non-randomized studies with the ROBINS-I tool

Study	Due to confounding	Due to selection of participants	In classification of interventions	Due to deviations from intended interventions	Due to missing data	In measurement of outcomes	In selection of the reported result	Overall
Condo 2012	Serious	Serious	Moderate	Low	Moderate	Low	Low	Serious
Dagdeviren 2021	Serious	Serious	Moderate	Low	Low	Low	Low	Serious
de Souza 2008	Serious	Serious	Moderate	Low	Low	Low	Low	Serious
Islam 2014	Serious	Serious	Moderate	Low	Moderate	Low	Low	Serious
Rodrigues 2011	Serious	Serious	Moderate	Low	Low	Low	Low	Serious
Savant 2016	Serious	Serious	Moderate	Low	Low	Low	Low	Serious
Thenarasu 2018	Serious	Serious	Moderate	Low	Low	Low	Low	Serious
Turkkahraman 2005	Serious	Serious	Moderate	Low	Low	Low	Low	Serious

### Data synthesis

**Fig. 2:**
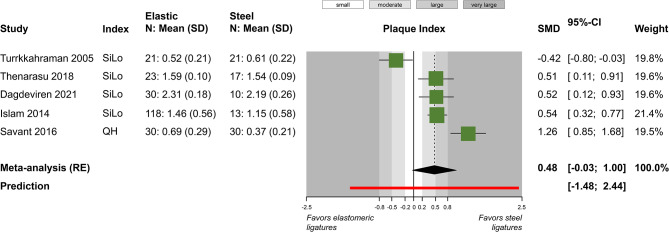
Meta-analysis comparing plaque index between fixed appliances ligated with elastomeric and stainless steel ligatures. CI, confidence interval; *N* number of patients; *QH* Quigley Hein index; *RE* random-effects model; *SD* standard deviation; *SiLo* Silness & Löe index; *SMD* standardized mean difference

**Fig. 3:**
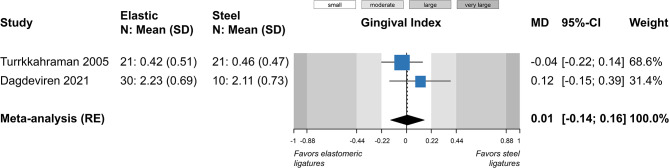
Meta-analysis comparing gingival index between fixed appliances ligated with elastomeric and stainless steel ligatures. *CI* confidence interval; *MD* mean difference; *N* number of patients; *RE* random-effects model; *SD* standard deviation

**Fig. 4:**
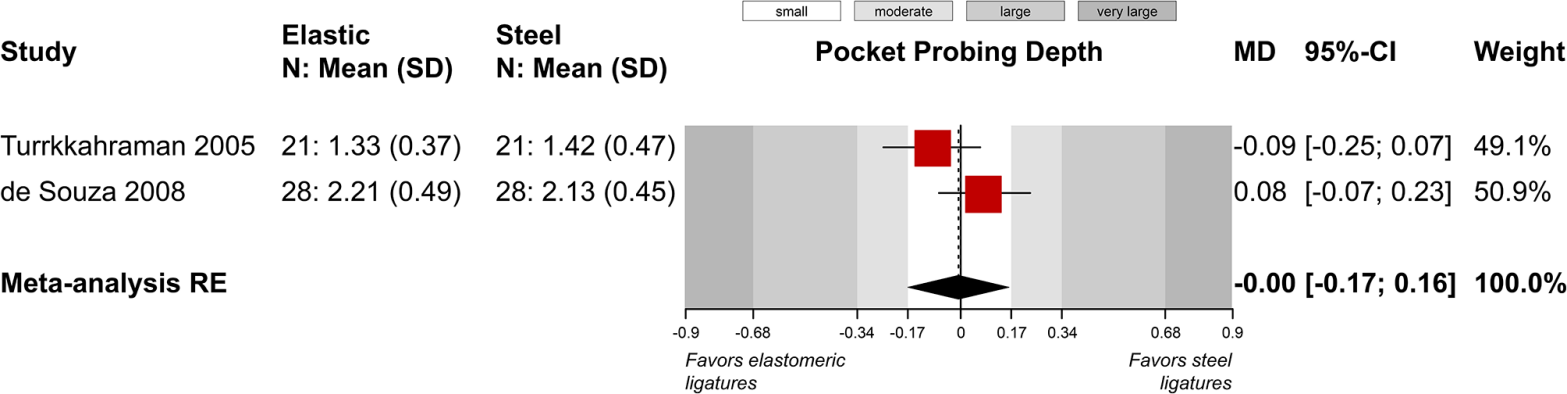
Meta-analysis comparing pocket probing depth between fixed appliances ligated with elastomeric and stainless steel ligatures. *CI* confidence interval; *MD* mean difference; *N* number of patients; *RE* random-effects model; *SD* standard deviation

**Fig. 5:**
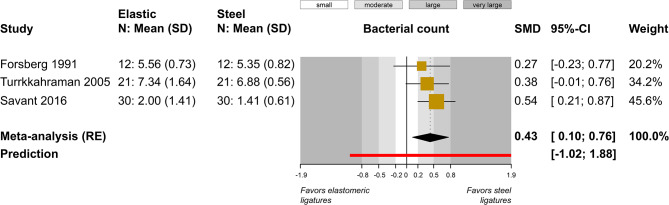
Meta-analysis comparing bacterial counts between fixed appliances ligated with elastomeric and stainless steel ligatures. *CI* confidence interval; *N* number of patients; *RE* random-effects model; *SD* standard deviation; *SMD* standardized mean difference

**Fig. 6:**
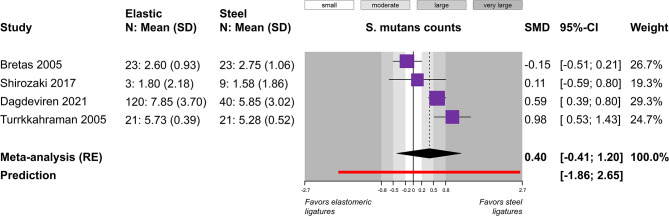
Meta-analysis comparing salivary Streptococcus mutans counts between fixed appliances ligated with elastomeric and stainless steel ligatures. *CI* confidence interval; *N* number of patients; *RE* random-effects model; *SD* standard deviation; *SMD* standardized mean difference

A total of five outcomes were assessed in a relatively similar manner from more than one study and were included in meta-analysis (Table 3). Meta-analysis of five studies did not find a statistically significant difference in plaque index between elastomeric and steel ligatures (5 studies; SMD = 0.48; 95% CI = -0.03 to 1.00; *P* = 0.07; Fig. 2), but great heterogeneity across studies was seen and 4 out of 5 studies pointed towards greater plaque accumulation with the former. Post hoc removal of the single study on the forest plot’s left side, which was responsible for the heterogeneity, led to somewhat larger difference (4 studies; SMD = 0.70; 95% CI = 0.13 to 1.27; *P* = 0.03), which was now marginally statistically significant. Similarly, no significant differences were found for gingival index (2 studies; MD = 0.01; *P* = 0.89; Fig. 3) and probing pocket depth (2 studies; MD = 0; *P* = 0.97; Fig. 4). Meta-analysis of three studies indicated that elastomeric ligatures were associated with increased bacterial load compared to steel ligatures (3 studies; SMD = 0.43; 95% CI = 0.10 to 0.76; *P* = 0.03; Fig. 5), with the effect being of moderate magnitude. Finally, no statistically significant difference was found in Streptococcus mutans counts between elastomeric and steel ligatures (4 studies; SMD = 0.40; *P* = 0.21; Fig. 6). Considerable between-study heterogeneity was seen also for this meta-analysis, but as most studies reported minimal differences between compared groups and the overall meta-analysis was similarly not statistically significance, this was deemed to be due to random variation.

**Table 3:** Meta-analyses comparing elastomeric to stainless steel ligatures

Outcome	Studies (Patients)	Effect (95% CI)	*P*	τ^2^ (95% CI)	I^2^ (95% CI)	Prediction
Plaque Index	5 (313)	SMD 0.48 (-0.03, 1.00)	0.07	0.31 (0.09, 2.91)	89% (76%, 95%)	-1.48, 2.44
Gingival Index	2 (82)	MD 0.01 (-0.14, 0.16)	0.89	0 (-)	0% (-)	-
Probing pocket depth	2 (98)	MD 0 (-0.17, 0.16)	0.97	0.01 (-)	57% (-)	-
Bacterial count	3 (126)	SMD 0.43 (0.10, 0.76)	0.03	0 (0, 0.69)	0% (0%, 90%)	-1.02, 1.88
S. mutans count	4 (260)	SMD 0.40 (-0.41, 1.20)	0.21	0.21 (0.04, 3.47)	84% (60%, 94%)	-1.86, 2.65

*CI* confidence interval; *MD* mean difference; *SMD* standardized mean difference

Table 4 shows the GRADE evaluation of strength of clinical recommendations from performed meta-analyses. In all instances, low quality of evidence was found, due to the inclusion of non-randomized studies that in many instances had methodological limitations that could pose a threat to their internal validity and increase their risk of bias. The results of the single statistically significant meta-analysis were back-translated to elastomeric ligatures having on average 0.30 × 10^5^ CFU/ml greater bacterial counts (95% CI 0.07 to 0.52 × 10^5^ CFU/ml) compared to steel ligatures.

**Table 4:** Summary of findings table according to the GRADE approach

	Anticipated absolute effects (95% CI)					
Outcome Studies (patients)	Steel group^a^	Difference in elastomeric group	Quality of the evidence (GRADE)^b^		What happens with elastomeric ligatures	Comment
Plaque index 5 studies (313 patients)	1.37	0.14 greater (0.01 smaller to 0.29 greater)	⨁⨁◯◯ Low^c^ due to bias, inconsistency		Little to no difference in plaque index	Based on an SMD for plaque indices of 0.48 (95% CI -0.03 to 1.00); back-translated to Silness & Löe plaque index using an average control SD of 0.29.
Gingival index 2 studies (82 studies)	1.27	0.01 greater (0.14º lower to 0.16 greater)	⨁⨁◯◯ Low^c^ due to bias		Little to no difference in gingival index	-
Probing pocket depth 2 studies (98 patients)	1.78	Same (0.17 smaller to 0.16 greater)	⨁⨁◯◯ Low^c^ due to bias, inconsistency		Little to no difference in pocket probing depth	-
Bacterial count (x10^5^ CFU/ml) 3 studies (126 patients)	6.13	0.30 greater (0.07 smaller to 0.52 greater)	⨁⨁◯◯ Low^c, d^ due to bias, inconsistency		Might be associated with greater bacterial count	Based on an SMD for bacterial counts of 0.43 (95% CI 0.10 to 0.76); back-translated using an average control SD of 0.69.
S. mutans count (log[CFU]) 4 studies (260 patients)	2.75	0.42 greater (0.43 smaller to 1.27 greater)	⨁⨁◯◯ Low^c^ due to bias, inconsistency		Little to no difference in S. mutans counts	Based on an SMD for S. mutans count of 0.40 (95% CI -0.41 to 1.20); back-translated using an control SD of 1.06.

Population: orthodontic patients receiving fixed-appliance treatment, ligated with either elastomeric ligatures; comparison: appliances ligated with stainless steel ligatures; setting: university clinics (Brazil, India, Italy, Pakistan, Sweden, and Turkey)

^a^ Response in the control group is based on the response of representative included studies or random-effects meta-analysis of the control response

^b^ Starts from “high”

^c^ Downgraded by two levels, due to serious potential issues with confounding, selection of participants, and deviation of intended intervention

^d^ Signs of inconsistency, as potential effects range from small to large; however, this affects only the precise identification of the effect magnitude, not the direction of effects

*CFU* colony forming unit; *CI* confidence interval; *SD* standard deviation; *SMD* standardised mean difference

Sensitivity analysis according to the design of the included studies can be found in Appendix 4. The results of the statistically significant meta-analysis on bacterial counts were no different depending on whether randomized or non-randomized studies were used (*P* = 0.45). In the assessment of Streptococcus mutans counts, significant differences were found between randomized and non-randomized studies, where the latter showed significantly more inflated negative effects (greater Streptococcus mutans counts) for elastomeric ligatures than the former (*P* < 0.001). As, however, the cumulative meta-analysis was not statistically significant, this does not affect overall recommendations.

## Discussion

### Result in context

The present review summarizes evidence from 11 studies involving 354 patients undergoing fixed appliance treatment with conventional (not self-ligating) brackets. All included studies compared the impact of metallic versus elastomeric ligatures on periodontal health and is, to our knowledge, the first of its kind.

Periodontal health was assessed among included studies through both clinical and microbiological parameters, including the plaque index, gingival index, probing pocket depth and counts of total bacteria or Streptococcus mutans. Orthodontic treatment with fixed appliances has been previously associated with increased biofilm accumulation and gingival inflammation around both self-ligating and conventionally ligated brackets [29, 30]. Additionally, orthodontic treatment with fixed appliances has been associated with minimal increases in probing pocket depth [31], which are however normalized after appliance removal [32]. Overall, clinical evidence indicates that orthodontic treatment under proper oral hygiene regimens is not associated with clinical attachment loss, even among patients with reduced but healthy periodontium [33–35]. At the same time, fixed appliance orthodontic treatment is associated with increased bacterial burden and Streptococcus mutans counts [36, 37], which although transient [32, 38], highlights the importance of fluoride supplementation to minimize the risk for dental caries or enamel demineralization [37].

The increased microbial colonization of elastomeric ligation modules compared to steel ligatures has traditionally been based on either anecdotal data [], data from in vitro studies [39–41] or in vivo retrieval studies [15, 42]. In the present review, this was the only consistent difference between ligation methods, with elastomeric ligatures showing moderately higher bacterial counts than steel ligatures (SMD = 0.4; Fig. 5) and with effects ranging from small to large (SMDs 0.10 to 0.8). It is important however to keep in mind that (i) included studies are small, which makes precise effect estimation uncertain; (ii) the wide prediction interval (ranging from − 1.0 to 1.9) indicated that even though the average trend indicates increased counts with elastomeric ligatures, this will not necessarily be the case for each single; (ii) all three studies were in high risk of bias, which indicated that caution is warranted by the interpretation of their findings. Possible explanations include among others that the complex microbial adhesion process is the result of several factors, such as specific lectin-similar reactions, electro-static interactions, and Van der Waals forces between the microorganisms and surface. A recent review [43] reported that bacterial adhesive strength is mainly determined by the amount and nature of contacts between surfaces and macromolecules on the bacterial surfaces, rather than from the physicochemical properties of surface materials. It is commonly believed that higher surface roughness (such as that of elastomeric rings) influences bacterial attachment mainly by increasing the surface area for microbial colonization. Moreover, they are reported to be more difficult to clean compared with smooth surfaces, like those of metallic materials [44–46]. However, other *in-vivo* or *in-situ* studies contradict this opinion and, according to the present findings, suggest that modification of surface roughness only plays a modest role in altering bacterial adherence and biofilm formation [47, 48]. Indeed, certain bacterial strains increase their adhesion when enhancing the material stiffness, independently from their other physico-chemical properties [49]. A possible explanation for this is that all surfaces exposed to the oral cavity are covered by the acquired pellicle within a short time [41]. This pellicle can level out surface roughness, modulating the physicochemical properties of the materials and, consequently, modifying the bacteria adherence [46, 50]. Another important issue is that periodontal health depends not only on the degree of biofilm formation, but also on the composition of the microbial community [43].

Even though the increased microbial colonization of elastomeric ligatures seems to be true, it does not necessarily translate to worse outcomes of periodontal health for the average patient compared to steel ligatures. Both elastomeric modules and elastic chains present considerable degradation that is accentuated by intraoral ageing [15, 51, 52], which is another reason why they are usually replaced at each appointment. This might partially explain why their increased bacterial load is not reflected on periodontal parameters. It should, however, be noted that these are the average effects of fixed appliance treatment and, as expected, considerable heterogeneity exists. This means that for some patients with suboptimal oral hygiene, the increased microbial load of elastomeric ligatures could contribute to the risk of periodontal inflammation. The existence of a structured oral health promotion protocol during orthodontic treatment is therefore of paramount importance [53] and novel motivation-enhancing interventions could potentially be beneficial in improving oral health and minimizing treatment-related adverse effects [54–59].

Ligation choice does not seem to be primarily influenced by differential microbial colonization and periodontal effects, but differences exist in the clinical performance of various ligation methods. Biomechanically, differences in applied force magnitude, torque expression, and frictional resistance have been reported between elastomeric and steel ligatures [60–62]. Additionally, unlike elastomeric ligatures, steel ligatures do not suffer from force decay phenomena [63–65] and can be left in place for longer periods and therefore be combined with longer intervals between appointments. Finally, elastomeric ligatures present color instability and show considerable staining after intraoral use [66–68], which can have a negative impact on esthetics.

### Limitations

This review has certain limitations in this review. The most significant is the inclusion of non-randomized studies, which are generally more prone to bias [69]. However, sensitivity analyses according to the study design did not find significant discrepancies between the results from the two study designs. Furthermore, all meta-analyses were informed by few studies with very limited sample sizes that could be biased [70] and present results should be confirmed by future studies with larger samples.

## Conclusions

Based on available evidence of low certainty from randomized and non-randomized clinical studies, there might be little clinically relevant differences in the periodontal effects of elastomeric or stainless steel ligatures used for orthodontic fixed appliances. However, the existing studies present serious methodological limitations and more well-designed prospective studies could help formulate robust clinical recommendations.

### Electronic supplementary material

Below is the link to the electronic supplementary material.

Supplementary Material 1

## Data Availability

The study’s dataset is openly available through Zenodo (10.5281/zenodo.10207373).

## References

[1] Balachandran P, Janakiram C (2021). Prevalence of malocclusion among 8–15 years old children, India - A systematic review and meta-analysis. J Oral Biol Craniofac Res.

[2] James A, Janakiram C, Meghana RV, Kumar VS, Sagarkar AR (2023). Impact of oral conditions on oral health-related quality of life among Indians- a systematic review and Meta-analysis. Health Qual Life Outcomes.

[3] Cooper-Kazaz R, Ivgi I, Canetti L, Bachar E, Tsur B, Chaushu S, Shalish M (2013). The impact of personality on adult patients’ adjustability to orthodontic appliances. Angle Orthod.

[4] Papageorgiou SN, Koletsi D, Iliadi A, Peltomaki T, Eliades T (2020). Treatment outcome with orthodontic aligners and fixed appliances: a systematic review with meta-analyses. Eur J Orthod.

[5] Campobasso A, Lo Muzio E, Battista G, Ciavarella D, Crincoli V, Lo Muzio L (2021). Taxonomic analysis of oral Microbiome during Orthodontic Treatment. Int J Dent.

[6] Freitas AO, Marquezan M, Nojima Mda C, Alviano DS, Maia LC (2014). The influence of orthodontic fixed appliances on the oral microbiota: a systematic review. Dent Press J Orthod.

[7] Gao L, Xu T, Huang G, Jiang S, Gu Y, Chen F (2018). Oral microbiomes: more and more importance in oral cavity and whole body. Protein Cell.

[8] Papageorgiou SN, Xavier GM, Cobourne MT, Eliades T (2018). Effect of orthodontic treatment on the subgingival microbiota: a systematic review and meta-analysis. Orthod Craniofac Res.

[9] Hussain U, Alam S, Rehman K, Antonoglou GN, Papageorgiou SN (2023). Effects of chlorhexidine use on periodontal health during fixed appliance orthodontic treatment: a systematic review and meta-analysis. Eur J Orthod.

[10] Koopman JE, van der Kaaij NC, Buijs MJ, Elyassi Y, van der Veen MH, Crielaard W (2015). The effect of fixed Orthodontic Appliances and Fluoride Mouthwash on the oral Microbiome of adolescents - A Randomized Controlled Clinical Trial. PLoS ONE.

[11] Forsberg CM, Brattström V, Malmberg E, Nord CE (1991). Ligature wires and elastomeric rings: two methods of ligation, and their association with microbial colonization of Streptococcus mutans and lactobacilli. Eur J Orthod.

[12] Baka ZM, Basciftci FA, Arslan U (2013). Effects of 2 bracket and ligation types on plaque retention: a quantitative microbiologic analysis with real-time polymerase chain reaction. Am J Orthod Dentofac Orthop.

[13] Al-Haifi HAA, Ishaq RAA, Al-Hammadi MSA (2021). Salivary pH changes under the effect of stainless steel versus elastomeric ligatures in fixed orthodontic patients: a single-center, randomized controlled clinical trial. BMC Oral Health.

[14] Sawhney R, Sharma R, Sharma K (2018). Microbial colonization on elastomeric ligatures during Orthodontic therapeutics: an overview. Turk J Orthod.

[15] Eliades T, Eliades G, Watts DC (1999). Structural conformation of in vitro and in vivo aged orthodontic elastomeric modules. Eur J Orthod.

[16] Türkkahraman H, Sayin MO, Bozkurt FY, Yetkin Z, Kaya S, Onal S (2005). Archwire ligation techniques, microbial colonization, and periodontal status in orthodontically treated patients. Angle Orthod.

[17] Skilbeck MG, Mei L, Mohammed H, Cannon RD, Farella M (2022). The effect of ligation methods on biofilm formation in patients undergoing multi-bracketed fixed orthodontic therapy - A systematic review. Orthod Craniofac Res.

[18] Arnold S, Koletsi D, Patcas R, Eliades T (2016). The effect of bracket ligation on the periodontal status of adolescents undergoing orthodontic treatment. A systematic review and meta-analysis. J Dent.

[19] Higgins JPT, Thomas J, Chandler J, et al. editors. (2020) Cochrane Handbook for Systematic Reviews of Interventions Version 6.1 (Updated September 2020). Cochrane. www.training.cochrane.org/handbook (9 September 2023, date last accessed).

[20] Page MJ, Moher D, Bossuyt PM, Boutron I, Hoffmann TC, Mulrow CD et al. PRISMA 2020 explanation and elaboration: updated guidance and exemplars for reporting systematic reviews. BMJ. 2021;372. 10.1136/bmj.n160 PMC8005925 33781993

[21] Sterne JA, Savović J, Page MJ, Elbers RG, Blencowe NS, Boutron I (2019). RoB 2: a revised tool for assessing risk of bias in randomised trials. BMJ.

[22] Sterne JA, Hernán MA, Reeves BC, Savović J, Berkman ND, Viswanathan M (2016). ROBINS-I: a tool for assessing risk of bias in non-randomised studies of interventions. BMJ.

[23] Papageorgiou SN (2014). Meta-analysis for orthodontists: part I–How to choose effect measure and statistical model. J Orthod.

[24] Langan D, Higgins JPT, Jackson D, Bowden J, Veroniki AA, Kontopantelis E (2019). A comparison of heterogeneity variance estimators in simulated random-effects meta-analyses. Res Synth Methods.

[25] IntHout J, Ioannidis JP, Rovers MM, Goeman JJ (2016). Plea for routinely presenting prediction intervals in meta-analysis. BMJ Open.

[26] Hussain U, Campobasso A, Noman M, Alam S, Mujeeb R, Shehzad S, Papageorgiou SN. Influence of elastomeric and steel ligatures on periodontal health during fixed appliance orthodontic treatment: a systematic review and meta-analysis [Data set]. Zenodo. 10.5281/zenodo.10207374. 10.1186/s40510-024-00520-8 PMC11180646 38880839

[27] Guyatt GH, Oxman AD, Schünemann HJ, Tugwell P, Knottnerus A (2011). GRADE guidelines: a new series of articles in the J Clin Epidemiol. J Clin Epidemiol.

[28] Carrasco-Labra A, Brignardello-Petersen R, Santesso N, Neumann I, Mustafa RA, Mbuagbaw L (2016). Improving GRADE evidence tables part 1: a randomized trial shows improved understanding of content in summary of findings tables with a new format. J Clin Epidemiol.

[29] Chhibber A, Agarwal S, Yadav S, Kuo CL, Upadhyay M (2018). Which orthodontic appliance is best for oral hygiene? A randomized clinical trial. Am J Orthod Dentofac Orthop.

[30] Marincak Vrankova Z, Rousi M, Cvanova M, Gachova D, Ruzicka F, Hola V (2022). Effect of fixed orthodontic appliances on gingival status and oral microbiota: a pilot study. BMC Oral Health.

[31] Bollen AM, Cunha-Cruz J, Bakko DW, Huang GJ, Hujoel PP (2008). The effects of orthodontic therapy on periodontal health: a systematic review of controlled evidence. J Am Dent Assoc.

[32] Ghijselings E, Coucke W, Verdonck A, Teughels W, Quirynen M, Pauwels M, Carels C, van Gastel J (2014). Long-term changes in microbiology and clinical periodontal variables after completion of fixed orthodontic appliances. Orthod Craniofac Res.

[33] Papageorgiou SN, Papadelli AA, Eliades T (2018). Effect of orthodontic treatment on periodontal clinical attachment: a systematic review and meta-analysis. Eur J Orthod.

[34] Papageorgiou SN, Antonoglou GN, Michelogiannakis D, Kakali L, Eliades T, Madianos P (2022). Effect of periodontal-orthodontic treatment of teeth with pathological tooth flaring, drifting, and elongation in patients with severe periodontitis: a systematic review with meta-analysis. J Clin Periodontol.

[35] Papageorgiou SN, Antonoglou GN, Eliades T, Martin C, Sanz M. Orthodontic treatment of patients with severe (stage IV) periodontitis. Semin Orthod 2024 [Epub ahead of print].

[36] Sifakakis I, Papaioannou W, Papadimitriou A, Kloukos D, Papageorgiou SN, Eliades T (2018). Salivary levels of cariogenic bacterial species during orthodontic treatment with thermoplastic aligners or fixed appliances: a prospective cohort study. Prog Orthod.

[37] Enerbäck H, Möller M, Nylén C, Ödman Bresin C, Östman Ros I, Westerlund A (2019). Effects of orthodontic treatment and different fluoride regimens on numbers of cariogenic bacteria and caries risk: a randomized controlled trial. Eur J Orthod.

[38] van Gastel J, Quirynen M, Teughels W, Coucke W, Carels C (2011). Longitudinal changes in microbiology and clinical periodontal parameters after removal of fixed orthodontic appliances. Eur J Orthod.

[39] Gameiro GH, Nouer DF, Cenci MS, Cury JA (2009). Enamel demineralization with two forms of archwire ligation investigated using an in situ caries model—a pilot study. Eur J Orthod.

[40] Garcez AS, Suzuki SS, Ribeiro MS, Mada EY, Freitas AZ, Suzuki H (2011). Biofilm retention by 3 methods of ligation on orthodontic brackets: a microbiologic and optical coherence tomography analysis. Am J Orthod Dentofac Orthop.

[41] Shalchi M, Hajian-Tilaki A, khanjani MS, Sabzgolin P, Aghajani Nargesi R (2018). Comparing Streptococcus Mutans adhesion by using different Orthodontic Bracket ligations: an in Vitro Study. J Dentomaxillofac Radiol Pathol Surg.

[42] Magno AF, Enoki C, Ito IY, Matsumoto MA, Faria G, Nelson-Filho P (2008). In-vivo evaluation of the contamination of super slick elastomeric rings by Streptococcus mutans in orthodontic patients. Am J Orthod Dentofac Orthop.

[43] Sterzenbach T, Helbig R, Hannig C, Hannig M (2020). Bioadhesion in the oral cavity and approaches for biofilm management by surface modifications. Clin Oral Investig.

[44] Quirynen M, Bollen CM (1995). The influence of surface roughness and surface-free energy on supra- and subgingival plaque formation in man. A review of the literature. J Clin Periodontol.

[45] Teughels W, Van Assche N, Sliepen I, Quirynen M (2006). Effect of material characteristics and/or surface topography on biofilm development. Clin Oral Implants Res.

[46] Park JW, Song CW, Jung JH, Ahn SJ, Ferracane JL (2012). The effects of surface roughness of composite resin on biofilm formation of Streptococcus mutans in the presence of saliva. Oper Dent.

[47] de Melo F, do Nascimento C, Souza DO, de Albuquerque RF (2017). Identification of oral bacteria on titanium implant surfaces by 16S rDNA sequencing. Clin Oral Implants Res.

[48] Bevilacqua L, Milan A, Del Lupo V, Maglione M, Dolzani L (2018). Biofilms developed on Dental Implant Titanium surfaces with different roughness: comparison between in Vitro and in vivo studies. Curr Microbiol.

[49] Guégan C, Garderes J, Le Pennec G, Gaillard F, Fay F, Linossier I (2014). Alteration of bacterial adhesion induced by the substrate stiffness. Colloids Surf B.

[50] McConnell MD, Liu Y, Nowak AP, Pilch S, Masters JG, Composto RJ (2010). Bacterial plaque retention on oral hard materials: effect of surface roughness, surface composition, and physisorbed polycarboxylate. J Biomed Mater Res A.

[51] Eliades T, Eliades G, Silikas N, Watts DC (2004). Tensile properties of orthodontic elastomeric chains. Eur J Orthod.

[52] Eliades T, Eliades G, Silikas N, Watts DC (2005). In vitro degradation of polyurethane orthodontic elastomeric modules. J Oral Rehabil.

[53] Gray D, McIntyre G (2008). Does oral health promotion influence the oral hygiene and gingival health of patients undergoing fixed appliance orthodontic treatment? A systematic literature review. J Orthod.

[54] Mohammed H, Rizk MZ, Wafaie K, Ulhaq A, Almuzian M (2019). Reminders improve oral hygiene and adherence to appointments in orthodontic patients: a systematic review and meta-analysis. Eur J Orthod.

[55] Tasios T, Papageorgiou SN, Papadopoulos MA, Tsapas A, Haidich AB (2019). Prevention of orthodontic enamel demineralization: a systematic review with meta-analyses. Orthod Craniofac Res.

[56] Fernández CE, Maturana CA, Coloma SI, Carrasco-Labra A, Giacaman RA (2021). Teledentistry and mHealth for Promotion and Prevention of Oral Health: a systematic review and Meta-analysis. J Dent Res.

[57] Patil S, Hedad IA, Jafer AA, Abutaleb GK, Arishi TM, Arishi SA, Arishi HA, Jafer M, Gujar AN, Khan S, Raj AT (2021). Effectiveness of mobile phone applications in improving oral hygiene care and outcomes in orthodontic patients. J Oral Biol Craniofac Res.

[58] Sangalli L, Savoldi F, Dalessandri D, Bonetti S, Gu M, Signoroni A, Paganelli C (2021). Effects of remote digital monitoring on oral hygiene of orthodontic patients: a prospective study. BMC Oral Health.

[57] Snider V, Homsi K, Kusnoto B, Atsawasuwan P, Viana G, Allareddy V, Gajendrareddy P, Elnagar MH. Effectiveness of AI-driven remote monitoring technology in improving oral hygiene during orthodontic treatment. Orthod Craniofac Res. 2023. Epub ahead of print. 10.1111/ocr.12666 37113065

[60] Gioka C, Eliades T (2004). Materials-induced variation in the torque expression of preadjusted appliances. Am J Orthod Dentofac Orthop.

[61] Papageorgiou SN, Keilig L, Vandevska-Radunovic V, Eliades T, Bourauel C (2017). Torque differences due to the material variation of the orthodontic appliance: a finite element study. Prog Orthod.

[62] Pieroni M, Ferraz Facury AGB, Santamaria-Jr M, Correr AB, Correr-Sobrinho L, Vedovello Filho M, Costa AR, Neves JG (2022). Comparison of the friction forces delivered by different elastomeric patterns and metal ligature on conventional metal brackets with a NiTi arch wire versus a self-ligating system: an in vitro study. Int Orthod.

[63] Taloumis LJ, Smith TM, Hondrum SO, Lorton L (1997). Force decay and deformation of orthodontic elastomeric ligatures. Am J Orthod Dentofac Orthop.

[64] Balhoff DA, Shuldberg M, Hagan JL, Ballard RW, Armbruster PC (2011). Force decay of elastomeric chains - a mechanical design and product comparison study. J Orthod.

[65] Halimi A, Benyahia H, Doukkali A, Azeroual MF, Zaoui F (2012). A systematic review of force decay in orthodontic elastomeric power chains. Int Orthod.

[66] Kim SH, Lee YK (2009). Measurement of discolouration of orthodontic elastomeric modules with a digital camera. Eur J Orthod.

[67] Aldrees AM, Al-Foraidi SA, Murayshed MS, Almoammar KA (2015). Color stability and force decay of clear orthodontic elastomeric chains: an in vitro study. Int Orthod.

[68] Kawabata E, Dantas VL, Kato CB, Normando D (2016). Color changes of esthetic orthodontic ligatures evaluated by orthodontists and patients: a clinical study. Dent Press J Orthod.

[69] Papageorgiou SN, Xavier GM, Cobourne MT (2015). Basic study design influences the results of orthodontic clinical investigations. J Clin Epidemiol.

[70] Cappelleri JC, Ioannidis JP, Schmid CH, de Ferranti SD, Aubert M, Chalmers TC, Lau J (1996). Large trials vs meta-analysis of smaller trials: how do their results compare?. JAMA.

