# Supplementary material for: Influence of elastomeric and steel ligatures on periodontal health during fixed appliance orthodontic treatment: a systematic review and meta-analysis
Source: Prog Orthod. 2024 Jun 17;25:24. doi: 10.1186/s40510-024-00520-8 (PMC11180646; doi:10.1186/s40510-024-00520-8)
Supplement: Supplementary file 1 — Supplementary Material 1 [file 40510_2024_520_MOESM1_ESM.pdf]

Appendix 1. Additional review methods and deviations from protocol.

Additional method details

- The count of Streptococcus mutans was transformed into a logarithmic value using a base-ten logarithm unless the data had already been reported in logarithmic form.
- For within-persons (cluster) randomized trials, all effect sizes were calculated with adjusted standard errors according to Cochrane guidelines to take into account clustering.
- Results of meta-anayses were presented in contour-enhanced forest plots illustrating the magnitude of observed effects [Papageorgiou, 2014]. This helps to assess the precision, heterogeneity, and clinical relevance of observed effects. For Mean Differences (MD), effects greater than half, one, and two Standard Deviations (SD) were used as cut-off points to denote small, moderate, large, and very large effects, using the average SD of the control group among studies included in the meta-analyses. For Standardised Mean Differences (SMD), cut-offs of 0.2, 0.5, and 0.8 were used.

Deviations from protocol

- Initially, reporting biases (including small-study effects and the possibility of publication bias) were planned to be assessed for meta-analyses with at least 10 studies [Sterne et al., 2011] through contour-enhanced funnel plots and Egger's linear regression test [Egger et al., 1997]. Ultimately, all meta-analyses included less than 10 studies and no reporting bias method was used.
- Alpha was going to be set at 10% for between-studies or between-subgroups heterogeneity (tests), but no such analyses were ultimately undertaken.

References for Appendix 1

Sterne JA, Sutton AJ, Ioannidis JP, Terrin N, Jones DR, Lau J, Carpenter J, Rücker G, Harbord RM, Schmid CH, Tetzlaff J, Deeks JJ, Peters J, Macaskill P, Schwarzer G, Duval S, Altman DG, Moher D, Higgins JP. Recommendations for examining and interpreting funnel plot asymmetry in meta-analyses of randomised controlled trials. BMJ. 2011;343:d4002.

Egger M, Davey Smith G, Schneider M, Minder C. Bias in meta-analysis detected by a simple, graphical test. BMJ. 1997;315(7109):629-34.

Papageorgiou SN. Meta-analysis for orthodontists: Part II--Is all that glitters gold? J Orthod. 2014;41(4):327-36.

Appendix 2. Search strategy used for all databases.

Database	Search strategy	Limits	Hits
Pubmed	(ligat*) AND ("elastic" OR "elastomeric" OR elastom* OR "steel" OR "stainless steel" OR steel OR elastomeric-ligated OR conventional ligation) AND orthodon* AND (treat* OR therap* OR "fixed appliance" OR "fixed appliances" OR brace* OR bracket* OR "multi-bracket" OR "multi-band" OR multibracket OR multiband)	Humans	452
Embase	Same as PubMed	Human	371
Scopus	Same as PubMed		220
WOS	Same as PubMed	Dentistry Oral Surgery Medicine	606
CDSR	Same as PubMed		2
CENTRAL	Same as PubMed		197
VHL	Same as PubMed		296
Last searchdate July 2023			

Appendix 3. Studies identified from the literature search with their inclusion / exclusion status (with reasons).

Paper	inclusion/exclusion	by
SharadAmbekar A, Kangane SK. Simple Elastomeric Chain Ligation Method for Palatally / Linguall Placed Lateral or Canines. Int J Orthod Milwaukee. 2017;28(1):47-8.	excluded/irrelevant	title
Normando D, de Araujo AM, Vieira Marques IdS, Barroso Tavares Dias CG, Mendes Miguel JA. Archwire cleaning after intraoral ageing: the effects on debris, roughness, and friction. European Journal of Orthodontics. 2013;35(2):223-9.	excluded/irrelevant	title
Israel M, Kusnoto B, Evans CA, Begole E. A comparison of traditional and computer-aided bracket placement methods. Angle Orthod. 2011;81(5):828-35.	excluded/irrelevant	title
Monteiro MRG, da Silva LE, Elias CN, Vilella OdV. Frictional resistance of self-ligating versus conventional brackets in different bracket-archwire-angle combinations. Journal of Applied Oral Science. 2014;22(3):228-34.	excluded/irrelevant	title
Ziegler TF. A modified technique for ligating impacted canines. Am J Orthod. 1977;72(6):665-70.	excluded/irrelevant	title
Shivapuja PK, Berger J. A comparative study of conventional ligation and self-ligation bracket systems. Am J Orthod Dentofacial Orthop. 1994;106(5):472-80.	excluded/irrelevant	title
Mendes Bde A, Neto Ferreira RA, Pithon MM, Horta MC, Oliveira DD. Physical and chemical properties of orthodontic brackets after 12 and 24 months: in situ study. J Appl Oral Sci. 2014;22(3):194-203.	excluded/irrelevant	title
Fernandes DJ, Almeida RCC, Quintão CCA, Elias CN, Miguel JAM. A estética no sistema de braquetes autoligáveis. Rev dent press ortodon ortopedi facial. 2008;13(3):97-103.	excluded/non-english language	abstract
Kumar D, Dua V, Mangla R, Solanki R, Solanki M, Sharma R. Frictional force released during sliding mechanics in nonconventional elastomerics and self-ligation: An in vitro comparative study. Indian J Dent. 2016;7(2):60-5.	excluded/irrelevant	title
Taloumis LJ, Smith TM, Hondrum SO, Lorton L. Force decay and deformation of orthodontic elastomeric ligatures. American Journal of Orthodontics and Dentofacial Orthopedics. 1997;111(1):1-11.	excluded/irrelevant	title
Forsberg CM, Brattström V, Malmberg E, Nord CE. Ligature wires and elastomeric rings: Two methods of ligation, and their association with microbial colonization of Streptococcus mutans and iactobacilli. The European Journal of Orthodontics. 1991 Oct 1;13(5):416-20.	included	
Monalisa R, Arun AV. Effects of variation in temperature on the permanent deformation of elastic chains. Research Journal of Pharmacy and Technology. 2018;11(6):2306-8.	excluded/irrelevant	title
Cobb NW, 3rd, Kula KS, Phillips C, Proffit WR. Efficiency of multi-strand steel, superelastic Ni-Ti and ion-implanted Ni-Ti archwires for initial alignment. Clin Orthod Res. 1998;1(1):12-9.	excluded/irrelevant	title
Shahid F, Rahman N, Khamis M, Husein A, Alam M. Pain perception with low-level laser therapy on orthodontic wire sequences. Journal of dental research. 2019;98(Spec Iss A).	excluded/ irrelevant	title
Folco AA, Benítez-Rogé SC, Iglesias M, Calabrese D, Pelizardi C, Rosa A, et al. Gingival response in orthodontic patients: Comparative study between self-ligating and conventional brackets. Acta Odontol Latinoam. 2014;27(3):120-4.	excluded/irrelevant	title
Sridharan K, Sandbhor S, Rajasekaran UB, Sam G, Ramees MM, Abraham EA. An in vitro Evaluation of Friction Characteristics of Conventional Stainless Steel and Self-ligating Stainless Steel Brackets with different Dimensions of Archwires in Various Bracket-archwire Combination. The journal of contemporary dental practice. 2017;18(8):660-4.	excluded/irrelevant	abstract
Lee RT. A splint for immediate surgical orthognathic fixation and release during orthodontic treatment. Eur J Orthod. 1991;13(3):209-11.	excluded/irrelevant	title
Nishi SE, Rahman NA, Basri R, Alam MK, Noor NFM, Zainal SA, et al. Surface Electromyography (sEMG) Activity of Masticatory Muscle (Masseter and Temporalis) with Three Different Types of Orthodontic Bracket. Biomed Res Int. 2021;2021:6642254.	excluded/irrelevant	title
Ewing M. Bond failure in clinical practice. Aust Orthod J. 2009;25(2):128-35.	excluded/irrelevant	title
Nct. Canine Retraction Using Different Bracket Slots' Sizes. https://clinicaltrialsgov/show/NCT05361863. 2022.	excluded/ irrelevant	title
Drks. Duration of tooth brushing with a manual and an electric toothbrush in children and adults with fixed appliances. https://trialsearchwhoint/Trial2aspx?TrialID=DRKS00012463. 2017.	excluded/ irrelevant	title
Budd S, Daskalogiannakis J, Tompson BD. A study of the frictional characteristics of four commercially available self-ligating bracket systems. Eur J Orthod. 2008;30(6):645-53.	excluded/irrelevant	title
Kapila S, Angolkar PV, Duncanson MG, Nanda RS. Evaluation of Friction between Edgewise Stainless-Steel Brackets and Orthodontic Wires of 4 Alloys. American Journal of Orthodontics and Dentofacial Orthopedics. 1990;98(2):117-26.	excluded/irrelevant	title
Shin JS, Oh KT, Hwang CJ. In vitro surface corrosion of stainless steel and NiTi orthodontic appliances. Aust Orthod J. 2003;19(1):13-8.	excluded/irrelevant	title
Moro N, Murakami T, Tanaka T, Ohto C. Uprighting of impacted lower third molars using brass ligature wire. Aust Orthod J. 2002;18(1):35-8.	excluded/irrelevant	title
Sánchez Domínguez M, Yeste Ojeda FM, Megía Córdoba A, Ventureira Pedrosa C. Sistemas autoligables de mínima fricción: ¿la fricción imperfecta? Ortod esp (Ed impr). 2011;51(2):84-94.	excluded/irrelevant	title
Lombardo L, Wierusz W, Toscano D, Lapenta R, Kaplan A, Siciliani G. Frictional resistance exerted by different lingual and labial brackets: an in vitro study. Prog Orthod. 2013;14:37.	excluded/irrelevant	title
Fleiner B, Härle F. [Active canine tooth movement by spongiosa graft in bilateral cheilognathopalatoschisis]. Fortschr Kieferorthop. 1991;52(4):252-6.	excluded/irrelevant	title
Bednar JR, Gruendeman GW, Sandrik JL. A comparative study of frictional forces between orthodontic brackets and arch wires. Am J Orthod Dentofacial Orthop. 1991;100(6):513-22.	excluded/irrelevant	title
Pandis N, Polychronopoulou A, Eliades T. Active or passive self-ligating brackets? A randomized controlled trial of comparative efficiency in resolving maxillary anterior crowding in adolescents. American journal of orthodontics and dentofacial orthopedics. 2010;137(1):12.e1-6; discussion -3.	excluded/irrelevant	title
Stutzmann JJ, Petrovic AG. Role of the lateral pterygoid muscle and meniscotemporomandibular frenum in spontaneous growth of the mandible and in growth stimulated by the postural hyperpropulsor. Am J Orthod Dentofacial Orthop. 1990;97(5):381-92.	excluded/irrelevant	title
Arnold S, Koletsi D, Patcas R, Eliades T. The effect of bracket ligation on the periodontal status of adolescents undergoing orthodontic treatment. A systematic review and meta-analysis. J Dent. 2016;54:13-24.	excluded/duplicate	duplicate
Lee Y-T, Liou EJ-W, Huang L-L, Wu H-J, Chen S-W. Effect of anodization on friction behavior of beta-titanium orthodontic archwires. Journal of Orofacial Orthopedics-Fortschritte Der Kieferorthopadie. 2021.	excluded/ irrelevant	title
Pan W, Li ZY, Zhang T, Shi EY, Deng JY, Wang YS, et al. [<i>In vitro</i> and <i>in vivo</i> anti-periodontitis effects of combination treatment of photodynamic and antibiotic therapies]. Zhonghua Kou Qiang Yi Xue Za Zhi. 2021;56(10):1011-9.	excluded/irrelevant	title
Curiel P, Salah P, Ayache W, inventors; American Orthodontics Corporation, assignee. Assembly formed by a bracket, a clip and a base for an orthodontic apparatus, and orthodontic apparatus comprising same patent US 08932053. 2015 Jan 13 2015.	excluded/duplicate	duplicate
Arun AV, Vaz AC. Frictional characteristics of the newer orthodontic elastomeric ligatures. Indian Journal of Dental Research. 2011;22(1):95-9.	excluded/irrelevant	title
Sueiro Flores MJ, Pereira Saraiva MdC, Scheicher GV, Panzeri Pires de Souza FdC, Sasso Stuani MB, Romano FL, et al. The effect of whitening toothpastes on polyurethane and silicone orthodontic clear ligatures: A clinical study. International Journal of Dental Hygiene. 2022;20(3):487-95.	excluded/ irrelevant	title
Suwa N, Watari F, Yamagata S, Iida J, Kobayashi M. Static-dynamic friction transition of FRP esthetic orthodontic wires on various brackets by suspension-type friction test. Journal of Biomedical Materials Research Part B-Applied Biomaterials. 2003;67B(2):765-71.	excluded/irrelevant	title
Wong H, Collins J, Tinsley D, Sandler J, Benson P. Does the bracket-ligature combination affect the amount of orthodontic space closure over three months? A randomized controlled trial. J Orthod. 2013;40(2):155-62.	excluded/ineligible comparator	full text
Akin M, Tezcan M, Ileri Z, Ayhan F. Incidence of white spot lesions among patients treated with self- and conventional ligation systems. Clin Oral Investig. 2015;19(6):1501-6.	excluded/irrelevant	title
Andreasen GF, Montagano L, Krell D. An Investigation of Linear Dimensional Changes as a Function of Temperature in an 0.010 Inch Cobalt-55-Substituted Annealed Nitinol Alloy Wire. American Journal of Orthodontics and Dentofacial Orthopedics. 1982;82(6):469-72.	excluded/irrelevant	title
Yi J, Li M, Li Y, Li X, Zhao Z. Root resorption during orthodontic treatment with self-ligating or conventional brackets: a systematic review and meta-analysis. BMC Oral Health. 2016;16(1):125.	excluded/ineligible comparator	full text
Dugdale CA, Malik OH, Waring DT. The "Floss-Ligature" Ligation Technique. Int J Orthod Milwaukee. 2015;26(4):53-4.	excluded/irrelevant	title
Articolo LC, Kusy RP. Influence of angulation on the resistance to sliding fixed appliances. American Journal of Orthodontics and Dentofacial Orthopedics. 1999;115(1):39-51.	excluded/irrelevant	title
Kaygisiz E, Uzuner FD, Yuksel S, Taner L, Çulhaoğlu R, Sezgin Y, et al. Effects of self-ligating and conventional brackets on halitosis and periodontal conditions. Angle Orthod. 2015;85(3):468-73.	excluded/irrelevant	title
Nct. The Omission of Pumice Prophylaxis on Bracket Failure Rates Using Self Etch Primers. https://clinicaltrialsgov/show/NCT04131855. 2019.	excluded/ irrelevant	title
Rinchuse DJ, Miles PG. Self-ligating brackets: Present and future. American Journal of Orthodontics and Dentofacial Orthopedics. 2007;132(2):216-22.	excluded/irrelevant	title
Brauchli LM, Senn C, Wichelhaus A. Active and passive self-ligation-a myth? Angle Orthod. 2011;81(2):312-8.	excluded/irrelevant	abstract
Rinchuse DJ, Rinchuse DJ. Modification of the bidimensional system. Orthodontics (Chic). 2011;12(1):10-21.	excluded/irrelevant	title
Varga S, Spalj S, Anic Milosevic S, Lapter Varga M, Mestrovic S, Trinajstic Zrinski M, et al. Changes of bite force and occlusal contacts in the retention phase of orthodontic treatment: A controlled clinical trial. Am J Orthod Dentofacial Orthop. 2017;152(6):767-77.	excluded/irrelevant	abstract
Gick MR, Nóbrega C, Benetti JJ, Jakob SR, Zucchi TU, Arsati F. Estudo comparativo do movimento de torque induzido pelos sistemas autoligantes e convencionais. Ortho Sci, Orthod sci pract. 2012;5(17):37-46.	excluded/irrelevant	title
[No authors] Erratum to Frictional forces in stainless steel and plastic brackets using four types of ligation (Dental Press Journal of Orthodontics, (2010), 15, 2, (82-86)). Dental Press Journal of Orthodontics. 2010;15(4):5.	excluded/ ineligible outcome	full text
Psaltis GL, Kupietzky A. A simplified isolation technique for preparation and placement of resin composite strip crowns. Pediatr Dent. 2008;30(5):436-8.	excluded/irrelevant	title
Crincoli V, Perillo L, Di Bisceglie MB, Balsamo A, Serpico V, Chiatante F, et al. Friction forces during sliding of various brackets for malaligned teeth: an in vitro study. ScientificWorldJournal. 2013;2013:871423.	excluded/irrelevant	title
Pandis N, Eliades T, Partowi S, Bourauel C. Forces exerted by conventional and self-ligating brackets during simulated first- and second-order corrections. Am J Orthod Dentofacial Orthop. 2008;133(5):738-42.	excluded/irrelevant	title
Alam MK. Laser assisted orthodontic tooth movement in saudi population: a randomized clinical trial. Bangladesh journal of medical science. 2019;18(2):385-90.	excluded/irrelevant	title
Tinsley D, O'Dwyer JJ, Benson PE. Fluoridated elastomers: in vivo versus in vitro fluoride release. J Orthod. 2003;30(4):317-22; discussion 298.	excluded/irrelevant	title
Chen YP, Li S. [Self-ligating vs. conventional brackets in the treatment of patients with Class II division 2 malocclusion: a clinical trial of dental and cephalometric analysis]. Zhonghua Kou Qiang Yi Xue Za Zhi. 2012;47(3):139-43.	excluded/irrelevant	title
Ehsani S, Mandich MA, El-Bialy TH, Flores-Mir C. Frictional resistance in self-ligating orthodontic brackets and conventionally ligated brackets. A systematic review. Angle Orthod. 2009;79(3):592-601.	excluded/irrelevant	title
Krishnadas A, Kashyap A, Pai VS, Adhikari S. Slide ligature: A low friction ligation system. Research Journal of Pharmaceutical, Biological and Chemical Sciences. 2016;7(5):2113-7.	excluded/ irrelevant	title
Pieroni M, Ferraz Facury AGB, Santamaria-Jr M, Correr AB, Correr-Sobrinho L, Vedovello Filho M, et al. Comparison of the friction forces delivered by different elastomeric patterns and metal ligature on conventional metal brackets with a NiTi arch wire versus a self-ligating system: An in vitro study. Int Orthod. 2022;20(2):100633.	excluded/ineligible comparator	full text
Machibya FM, Bao X, Zhao L, Hu M. Treatment time, outcome, and anchorage loss comparisons of self-ligating and conventional brackets. Angle Orthod. 2013;83(2):280-5.	excluded/irrelevant	abstract
Ireland AJ, Sherriff M, McDonald F. Effect of bracket and wire composition on frictional forces. Eur J Orthod. 1991;13(4):322-8.	excluded/irrelevant	title
Archambault A, Major TW, Carey JP, Heo G, Badawi H, Major PW. A comparison of torque expression between stainless steel, titanium molybdenum alloy, and copper nickel titanium wires in metallic self-ligating brackets. Angle Orthod. 2010;80(5):884-9.	excluded/irrelevant	title
Sabarinath VP, Sreeja R, Aljehani GA, Bajrai SS. Improved indirect bonding of self-ligating brackets. J Clin Orthod. 2013;47(12):744.	excluded/irrelevant	title
Lisniewska-Machorowska B, Cannon J, Williams S, Bantleon H-P. Evaluation of force systems from a "free-end" force system. Am J Orthod Dentofacial Orthop. 2008;133(6):791.e1-10.	excluded/irrelevant	title
Jung BA, Becker C, Wehrbein H. Uprighting and distalisation of first permanent maxillary molars in patients with undermining resorption: a case report. Eur J Paediatr Dent. 2011;12(2):128-30.	excluded/ irrelevant	title

Nct. Anchorage Reinforcement in Bimaxillary Proclination Cases. https://clinicaltrialsgov/show/NCT04705545. 2021.	excluded/ irrelevant	title
Negreiros PO, Freitas KMS, Pinzan-Vercelino CRM, Janson G, Freitas MR. Smile attractiveness in cases treated with self-ligating and conventional appliances with and without rapid maxillary expansion. Orthod Craniofac Res. 2020;23(4):413-8.	excluded/irrelevant	title
Ge N, Peng J, Yu L, Huang S, Xu L, Su Y, et al. Orthodontic treatment induces Th17/Treg cells to regulate tooth movement in rats with periodontitis. Iran J Basic Med Sci. 2020;23(10):1315-22.	excluded/ irrelevant	title
Thermac G, Morgon L, Godeneche J. [Friction: self-ligating brackets]. Orthod Fr. 2008;79(4):239-49.	excluded/irrelevant	title
Lin JH, Chang CH, Eugene RW. Vertical incision subperiosteal tunnel access and three-dimensional OBS lever arm to recover a labially-impacted canine: Differential biomechanics to control root resorption. Apos Trends in Orthodontics. 2019;9(1):7-18.	excluded/irrelevant	title
Kamelchuk LS, Rossouw PE. Development of a laboratory model to test kinetic orthodontic friction. Seminars in Orthodontics. 2003;9(4):251-61.	excluded/irrelevant	title
Gualano C, inventor; OrthoAmerica Holdings LLC, assignee. Method for producing a customized orthodontic appliance patent US 11382719. 2022 Jul 12 2022.	excluded/irrelevant	title
Isrctn. A prospective randomized trial investigating lower incisor inclination and mandibular arch dimensional changes of two pre-adjusted edgewise orthodontic bracket systems in non-extraction cases. https://trialsearchwhoint/Trial2aspx?TrialID=ISRCTN67900267. 2007.	excluded/ irrelevant	title
Yassir YA, McIntyre GT, Bearn DR. The impact of labial fixed appliance orthodontic treatment on patient expectation, experience, and satisfaction: an overview of systematic reviews. Eur J Orthod. 2020;42(3):223-30.	excluded/irrelevant	title
Sathler R, Silva RG, Janson G, Branco NCC, Zanda M. Desmistificando os braquetes autoligáveis. Dental Press Journal of Orthodontics. 2011;16(2):e1-e8.	excluded/non-english language	title
Sivakumar A, Gandhi S, Valiathan A. Re: Failure rate of self ligating and edgewise brackets bonded with conventional acid etching and a self etching primer: a prospective in vivo study. Angle Orthod. 2006; 76: 119-122. Angle Orthod. 2006;76(5):iii; author reply iii.	excluded/irrelevant	title
Parmar NP, Thompson GL, Atack NE, Ireland AJ, Sherriff M, Haworth JA. Microbial colonisation associated with conventional and self-ligating brackets: a systematic review. J Orthod. 2022;49(2):151-62.	excluded/review	full text
Hirai M, Nakajima A, Kawai N, Tanaka E, Igarashi Y, Sakaguchi M, et al. Measurements of the torque moment in various archwire-bracket-ligation combinations. Eur J Orthod. 2012;34(3):374-80.	excluded/irrelevant	title
Kim Y-J, Lee D-Y, Lee J-Y, Lim Y-K. The effect of silver ion-releasing elastomers on mutans streptococci in dental plaque. Korean Journal of Orthodontics. 2012;42(2):87-93.	excluded/ineligible comparatore	full text
Demange C. Modern biomechanical use of shape memory archwires and self ligating brackets: Theoretical and clinical aspects. Acta Stomatologica Croatica. 2017;51(2):165-6.	excluded/ irrelevant	title
Tecco S, Di Iorio D, Nucera R, Di Bisceglie B, Cordasco G, Festa F. Evaluation of the friction of self-ligating and conventional bracket systems. Eur J Dent. 2011;5(3):310-7.	excluded/irrelevant	title
Huntley PN. A modified over-tie for the ligation of Incognito™ lingual fixed appliances. J Orthod. 2013;40(3):244-8.	excluded/irrelevant	title
Didier VF, Almeida-Pedrin RR, Pedron Oltramari PV, Freire Fernandes TM, Lima Dos Santos L, Cláudia de Castro Ferreira Conti A. Influence of orthodontic appliance design on employment hiring preferences. Am J Orthod Dentofacial Orthop. 2019;156(6):758-66.	excluded/irrelevant	title
Moresca RC, Vigorito JW, Dominguez GC, Tortamano A, Moraes DR, Moro A, et al. Effects of active and passive lacebacks on antero-posterior position of maxillary first molars and central incisors. Braz Dent J. 2012;23(4):433-7.	excluded/irrelevant	title
Schumacher HA, Bourauel C, Drescher D. Influence of the Ligature on the Friction between Bracket and Arch Wire. Fortschritte der Kieferorthopaedie. 1990;51(2):106-16.	excluded/irrelevant	title
Mehta A, Paramshivam G, Chugh VK, Singh S, Halkai S, Kumar S. Effect of light-curable fluoride varnish on enamel demineralization adjacent to orthodontic brackets: an in-vivo study. Am J Orthod Dentofacial Orthop. 2015;148(5):814-20.	excluded/irrelevant	title
Dudnik OV, Mamedov AA, Dybov AM, Kharke VV. Clinical and diagnostic rationale for the final stage of orthodontic treatment. Stomatologiia. 2017;96(2):43-50.	excluded/irrelevant	title
Manni A, Mutinelli S, Pasini M, Mazzotta L, Cozzani M. Herbst appliance anchored to miniscrews with 2 types of ligation: Effectiveness in skeletal Class II treatment. Am J Orthod Dentofacial Orthop. 2016;149(6):871-80.	excluded/ineligible outcome	full text
Queiroz GV, Ballester RY, De Paiva JB, Neto JR, Galon GM. Comparative study of frictional forces generated by NiTi archwire deformation in different orthodontic brackets: In vitro evaluation. Dental Press Journal of Orthodontics. 2012;17(4):45-50.	excluded/irrelevant	title
Morikawa Y, Yotsui Y, Matsumoto N. Effect of orthodontic appliances on magnetic resonance images. Shika Igaku. 2008;71(1):35-48.	excluded/ irrelevant	title
Verse T. [Tongue fixation in OSA]. HNO. 2010;58(12):1190-1.	excluded/irrelevant	title
Iriarte AJ, Ortiz S, Cubides-Flechas K, Olaya C, Jaimes-Monroy G. Sliding resistance of rectangular vs. beveled archwires in two self-ligating brackets: a finite element study. Acta odontologica latinoamericana : AOL. 2018;31(3):149-55.	excluded/irrelevant	title
Nct. Evaluation of the Efficacy of a Messaging Application to Improve Oral Hygiene in Orthodontic Patients.ECA. https://clinicaltrialsgov/show/NCT05527613. 2022.	excluded/ irrelevant	title
Bergamo AZN, Nelson-Filho P, do Nascimento C, Casarin RCV, Casati MZ, Andrucioli MCD, et al. Cytokine profile changes in gingival crevicular fluid after placement different brackets types. Arch Oral Biol. 2018;85:79-83.	excluded/ineligible comparator	full text
Chain S, Tan K, Loomba A, Seth P, Sarkar SR. Double-Ligation Technique for Rotated Teeth. J Clin Orthod. 2016;50(4):258-.	excluded/irrelevant	abstract
Cash A, Curtis R, Garrigia-Majo D, McDonald F. A comparative study of the static and kinetic frictional resistance of titanium molybdenum alloy archwires in stainless steel brackets. Eur J Orthod. 2004;26(1):105-11.	excluded/irrelevant	title
Tupinamba RA, Claro CAdA, Pereira CA, Nobrega CJP, Claro APRA. Bacterial adhesion on conventional and self-ligating metallic brackets after surface treatment with plasma-polymerized hexamethyldisiloxane. Dental press journal of orthodontics. 2017;22(4):77-85.	excluded/irrelevant	full text
Khatri JM, Vispute SS, Kolhe VD, Sawant SS, Salve RS. Ligation ties in orthodontics. International Journal of Orthodontic Rehabilitation. 2020;11(4):193-8.	excluded/irrelevant	title
Shook C, Kim SM, Burnheimer J. Maxillary arch width and buccal corridor changes with Damon and conventional brackets: A retrospective analysis. Angle Orthod. 2016;86(4):655-60.	excluded/irrelevant	title
Kannan MS, Murali RV, Kishorekumar S, Gnanashanmugam K, Jayanth V. Comparison of frictional resistance of esthetic and semi-esthetic self-ligating brackets. J Pharm Bioallied Sci. 2015;7(Suppl 1):S116-20.	excluded/irrelevant	title
Lee Y, Lee DY, Kim YJ. Dimensional accuracy of ceramic self-ligating brackets and estimates of theoretical torsional play. Angle Orthod. 2016;86(5):804-9.	excluded/irrelevant	title
Bergamo AZ, Nelson-Filho P, Romano FL, da Silva RA, Saraiva MC, da Silva LA, et al. Gingival crevicular fluid volume and periodontal parameters alterations after use of conventional and self-ligating brackets. J Orthod. 2016;43(4):260-7.	excluded/ineligible intervention	full text
do Nascimento LE, de Souza MM, Azevedo AR, Maia LC. Are self-ligating brackets related to less formation of Streptococcus mutans colonies? A systematic review. Dental Press J Orthod. 2014;19(1):60-8.	excluded/Review	abstract
Kusy RP, Whitley JQ. Resistance to sliding of orthodontic appliances in the dry and wet states: influence of archwire alloy, interbracket distance, and bracket engagement. J Biomed Mater Res. 2000;52(4):797-811.	excluded/irrelevant	title
Irvine R, Power S, McDonald F. The effectiveness of laceback ligatures: a randomized controlled clinical trial. J Orthod. 2004;31(4):303-11; discussion 0.	excluded/irrelevant	title
[No authors] Mandibular arch orthodontic treatment stability using passive self-ligating and conventional systems in adults: a randomized controlled trial. Korean journal of orthodontics. 2017;47(1):11-20.	excluded/irrelevant	title
Dai K, Chu Y. Studies and applications of NiTi shape memory alloys in the medical field in China. Biomed Mater Eng. 1996;6(4):233-40.	excluded/irrelevant	abstract
Neela PK, Tatikonda VK, Syed MW, Mamillapalli PK, Sesham VM, Keesara S. Influence of orthodontic brackets and permanent retainers on the diagnostic image quality of MRI scans: A preliminary study. Dent Med Probl. 2021;58(4):499-508.	excluded/irrelevant	title
Huang T-H, Luk H-S, Hsu Y-C, Kao C-T. An in vitro comparison of the frictional forces between archwires and self-ligating brackets of passive and active types. European Journal of Orthodontics. 2012;34(5):625-32.	excluded/irrelevant	title
Echarri P, Pedernera M. Actualización en el posicionamiento de brackets para cementado indirecto. Ortodoncia. 2013;76(154):20-7.	excluded/non-english language	title
Baccetti T, Franchi L, Camporesi M. Forces in the presence of ceramic versus stainless steel brackets with unconventional vs conventional ligatures. Angle Orthod. 2008;78(1):120-4.	excluded/irrelevant	title
Berger JL. The influence of the SPEED bracket's self-ligating design on force levels in tooth movement: a comparative in vitro study. Am J Orthod Dentofacial Orthop. 1990;97(3):219-28.	excluded/irrelevant	abstract
Baek SH, Kim NY, Paeng JY, Kim MJ. Trifocal distraction-compression osteosynthesis in conjunction with passive self-ligating brackets for the reconstruction of a large bony defect and multiple missing teeth. Am J Orthod Dentofacial Orthop. 2008;133(4):601-11.	excluded/irrelevant	title
Bishara SE, Ajlouni R, Laffoon J, Warren J. Effects of modifying the adhesive composition on the bond strength of orthodontic brackets. Angle Orthod. 2002;72(5):464-7.	excluded/irrelevant	title
Kim S, Yonemitsu I, Takemura H, Shimoda K, Suga K, Soga K, et al. Influence of different ligation methods on force and moment generation in a simulated condition of the maxillary crowded anterior dentition with linguo-version and rotation. Bio-Medical Materials and Engineering. 2022;33(6):453-63.	excluded/irrelevant	title
Kim TK, Kim KD, Baek SH. Comparison of frictional forces during the initial leveling stage in various combinations of self-ligating brackets and archwires with a custom-designed typodont system. Am J Orthod Dentofacial Orthop. 2008;133(2):187.e15-24.	excluded/irrelevant	title
Liebenberg WH. Manipulation of rubber dam septa: an aid to the meticulous isolation of splinted prostheses. J Endod. 1995;21(4):208-11.	excluded/irrelevant	title
Bian C, Zhang K, Yang K, Bai Y, Zhang N. An in vivo and in vitro study on the force degradation and surface morphology of the orthodontic elastic ligatures. American journal of orthodontics and dentofacial orthopedics : official publication of the American Association of Orthodontists, its constituent societies, and the American Board of Orthodontics. 2022.	excluded/irrelevant	title
Panda S, Verma V, Sachan A, Singh K. Perception of pain due to various orthodontic procedures. Quintessence Int. 2015;46(7):603-9.	excluded/ irrelevant	title
Henriques JFC, Higa RH, Semenara NT, Janson G, Fernandes TMF, Sathler R. Evaluation of deflection forces of orthodontic wires with different ligation types. Brazilian Oral Research. 2017;31:1-7.	excluded/irrelevant	title
Husain N, Kumar A. Frictional resistance between orthodontic brackets and archwire: an in vitro study. The journal of contemporary dental practice. 2011;12(2):91-9.	excluded/irrelevant	title
Nucera R, Gatto E, Borsellino C, Aceto P, Fabiano F, Matarese G, et al. Influence of bracket-slot design on the forces released by superelastic nickel-titanium alignment wires in different deflection configurations. Angle Orthod. 2014;84(3):541-7.	excluded/irrelevant	title
Ivashchenko NI. [Experience in using the surgical-orthodontic method proposed by V. V. Donskoi for treating mandibular fractures]. Stomatologiia. 1990;69(6):40-2.	excluded/irrelevant	title
Franchi L, Baccetti T, Camporesi M, Lupoli M. Maxillary arch changes during leveling and aligning with fixed appliances and low-friction ligatures. Am J Orthod Dentofacial Orthop. 2006;130(1):88-91.	excluded/irrelevant	title
Miresmaeili A, Farhadian N, Rezaei-soufi L, Saharkhizan M, Veisi M. Effect of carbon dioxide laser irradiation on enamel surface microhardness around orthodontic brackets. Am J Orthod Dentofacial Orthop. 2014;146(2):161-5.	excluded/irrelevant	title
Stasinopoulos D, Papageorgiou SN, Kirsch F, Daratsianos N, Jäger A, Bourauel C. Failure patterns of different bracket systems and their influence on treatment duration: A retrospective cohort study. Angle Orthod. 2018;88(3):338-47.	excluded/irrelevant	title
Har-Zion G. Self-ligation: a clinician's point of view. Am J Orthod Dentofacial Orthop. 2009;136(6):756-7.	excluded/review	title
Ireland AJ, Soro V, Sprague SV, Harradine NW, Day C, Al-Anezi S, et al. The effects of different orthodontic appliances upon microbial communities. Orthod Craniofac Res. 2014;17(2):115-23.	excluded/ineligible comparator	full text
Fleming PS, DiBiase AT, Sarri G, Lee RT. Comparison of mandibular arch changes during alignment and leveling with 2 preadjusted edgewise appliances. Am J Orthod Dentofacial Orthop. 2009;136(3):340-7.	excluded/irrelevant	title
Harradine NW, Birnie DJ. The clinical use of Activa self-ligating brackets. Am J Orthod Dentofacial Orthop. 1996;109(3):319-28.	excluded/irrelevant	title
Sander C, Sander FM, Sander FG. The derotation of premolars and canines with NiTi elements. J Orofac Orthop. 2006;67(2):117-26.	excluded/irrelevant	title
Araújo RC, Bichara LM, Araujo AM, Normando D. Debris and friction of self-ligating and conventional orthodontic brackets after clinical use. Angle Orthod. 2015;85(4):673-7.	excluded/irrelevant	abstract

Sorake A, Jayakrishnan U, Suneja R, Sam S, Abraham T, Jayaraj A, et al. Lingual Bracket Systems with Self Etching Primers- An In Vitro Study to Evaluate Shear Bond Strength. J Contemp Dent Pract. 2019;20(2):166-72.	excluded/irrelevant	abstract
Pizzoni L, Ravnholt G, Melsen B. Frictional forces related to self-ligating brackets. Eur J Orthod. 1998;20(3):283-91.	excluded/irrelevant	title
Qamruddin I, Alam MK, Mahroof V, Fida M, Khamis MF, Husein A. Effects of low-level laser irradiation on the rate of orthodontic tooth movement and associated pain with self-ligating brackets. Am J Orthod Dentofacial Orthop. 2017;152(5):622-30.	excluded/irrelevant	title
Johansson K, Lundström F. Orthodontic treatment efficiency with self-ligating and conventional edgewise twin brackets: a prospective randomized clinical trial. Angle Orthod. 2012;82(5):929-34.	excluded/irrelevant	title
Yeh CL, Kusnoto B, Viana G, Evans CA, Drummond JL. In-vitro evaluation of frictional resistance between brackets with passive-ligation designs. American Journal of Orthodontics and Dentofacial Orthopedics. 2007;131(6):704.e11-.e22.	excluded/ineligible comparator	full text
Yatabe K. [Two cases of orthodontia treated with elastic thread ligature]. Shikwa Gakuho. 1968;68(8):79-82.	excluded/ineligible outcome	full text
Suyama H, Higashi K, Nakata S, Nakasima A. New edgewise bracket with rounded slot and variable ligation. J Clin Orthod. 1995;29(6):398-402.	excluded/ irrelevant	title
Fourie Z, Ozcan M, Sandham A. Effect of dental arch convexity and type of archwire on frictional forces. Am J Orthod Dentofacial Orthop. 2009;136(1):14.e1-7; discussion -5.	excluded/irrelevant	title
Cassetta M, Altieri F. The influence of mandibular third molar germectomy on the treatment time of impacted mandibular second molars using brass wire: a prospective clinical pilot study. Int J Oral Maxillofac Surg. 2017;46(7):905-11.	excluded/irrelevant	title
Nalçacı R, Özat Y, Çokakoğlu S, Türkkahraman H, Önal S, Kaya S. Effect of bracket type on halitosis, periodontal status, and microbial colonization. Angle Orthod. 2014;84(3):479-85.	excluded/irrelevant	title
Kanagasabapathy B, Varadharaja M, Saravanan R, Kumar V, Mahalakshmi R, Ninan R, et al. Frictional forces produced by three different ligation methods in two different types of brackets in 0.016 nickel-titanium wire: An in vitro study. Journal of Pharmacy and Bioallied Sciences. 2021;13(6):S1624-S7.	excluded/irrelevant	title
Voudouris JC. Interactive edgewise mechanisms: form and function comparison with conventional edgewise brackets. Am J Orthod Dentofacial Orthop. 1997;111(2):119-40.	excluded/irrelevant	abstract
Rosvall MD, Fields HW, Ziuchkovski J, Rosenstiel SF, Johnston WM. Attractiveness, acceptability, and value of orthodontic appliances. Am J Orthod Dentofacial Orthop. 2009;135(3):276.e1-12; discussion -7.	excluded/irrelevant	title
Weltman B, Vig KWL, Fields HW, Shanker S, Kaizar EE. Root resorption associated with orthodontic tooth movement: a systematic review. Am J Orthod Dentofacial Orthop. 2010;137(4):462-76; discussion 12A.	excluded/irrelevant	title
Wagner D, Didier P, Peltier L, Piotrowski B, Laheurte P. Innovative contribution of computer-aided design for optimal brackets' placement during in vitro mechanical characterization of orthodontic archwires. Computer Methods in Biomechanics and Biomedical Engineering. 2021;24(SUPPL 1):S160-S2.	excluded/irrelevant	title
O'Dywer L, Littlewood SJ, Rahman S, Spencer RJ, Barber SK, Russell JS. A multi-center randomized controlled trial to compare a self-ligating bracket with a conventional bracket in a UK population: Part 1: Treatment efficiency. Angle Orthod. 2016;86(1):142-8.	excluded/irrelevant	title
Dowling PA, Jones WB, Lagerstrom L, Sandham JA. An investigation into the behavioural characteristics of orthodontic elastomeric modules. Br J Orthod. 1998;25(3):197-202.	excluded/irrelevant	title
Nct. Evaluation of the Rate of Maxillary En-masse Retraction Using 0.018-inch Versus 0.022-inch Slot Brackets in Adults. https://clinicaltrialsgov/show/NCT04468295. 2020.	excluded/ irrelevant	title
Tselepis M, Brockhurst P, West VC. The Dynamic Frictional Resistance between Orthodontic Brackets and Arch Wires. American Journal of Orthodontics and Dentofacial Orthopedics. 1994;106(2):131-8.	excluded/irrelevant	title
Park J-B, Yoo J-A, Mo S-S, Choi K-C, Kim Y-J, Han SH, et al. Effect of friction from differing vertical bracket placement on the force and moment of NiTi wires. Korean Journal of Orthodontics. 2011;41(5):337-45.	excluded/irrelevant	title
Corghi RG, Malavazi DF, Quintela MM, Aquino DR, Silva HGd, Roman-Torres CVG. Avaliação clínica periodontal de indivíduos portadores de aparelhos ortodônticos com braquetes convencionais e autoligáveis. Periodontia. 2014;24(1):30-4.	excluded/non-english language	title
Olszewska A, Hańć A, Barałkiewicz D, Rzymski P. The contribution of orthodontic braces to aluminum exposure in humans: an experimental in vitro study. Environ Sci Pollut Res Int. 2020;27(4):4541-5.	excluded/irrelevant	title
Al-Kharsa SS, Masoud AI. A Proposed Method for Covering a Mini-Screw Head While Maintaining Space for Ligation. Int J Orthod Milwaukee. 2017;28(1):65-6.	excluded/irrelevant	title
Akgun OM, Altug H, Karacay S, Guven Polat G, Duyan S, Bedir O. Effect of 2 elastomeric ligatures on microbial flora and periodontal status in orthodontic patients. Am J Orthod Dentofacial Orthop. 2014;145(5):667-71.	excluded/ineligible comparator	full text
Dridi A, Bensalah W, Mezlini S, Tobji S, Zidi M. Influence of bio-lubricants on the orthodontic friction. J Mech Behav Biomed Mater. 2016;60:1-7.	excluded/irrelevant	title
Sifakakis I, Pandis N, Makou M, Eliades T, Katsaros C, Bourauel C. A comparative assessment of torque generated by lingual and conventional brackets. Eur J Orthod. 2013;35(3):375-80.	excluded/irrelevant	title
Takada M, Nakajima A, Kuroda S, Horiuchi S, Shimizu N, Tanaka E. In vitro evaluation of frictional force of a novel elastic bendable orthodontic wire. Angle Orthodontist. 2018;88(5):602-10.	excluded/irrelevant	title
Zhou JG, Feng JY. [Analysis of the effect of relieving dental crowding by using a newly designed low frictional force single-wing bracket on typodont model]. Shanghai Kou Qiang Yi Xue. 2011;20(6):608-10.	excluded/irrelevant	title
Vajaria R, BeGole E, Kusnoto B, Galang MT, Obrez A. Evaluation of incisor position and dental transverse dimensional changes using the Damon system. Angle Orthod. 2011;81(4):647-52.	excluded/irrelevant	title
Montasser MA, Keilig L, Bourauel C. Archwire diameter effect on tooth alignment with different bracket-archwire combinations. Am J Orthod Dentofacial Orthop. 2016;149(1):76-83.	excluded/irrelevant	title
Schumacher HA, Bourauel C, Drescher D. [The effect of the ligature on the friction between bracket and arch]. Fortschr Kieferorthop. 1990;51(2):106-16.	excluded/irrelevant	title
Nakano T, Nakajima A, Watanabe H, Osada A, Namura Y, Yoneyama T, et al. Evaluation of torque moment in esthetic brackets from bendable alloy wires. Angle Orthodontist. 2021;91(5):656-63.	excluded/irrelevant	title
DiBiase AT, Nasr IH, Scott P, Cobourne MT. Duration of treatment and occlusal outcome using Damon3 self-ligated and conventional orthodontic bracket systems in extraction patients: a prospective randomized clinical trial. Am J Orthod Dentofacial Orthop. 2011;139(2):e111-6.	excluded/irrelevant	title
Vale F, Maló L, Caramelo F, Ramos J, Cavaleiro J. Dynamic behavior and surface characteristics of conventional and self-ligating brackets. Revista Portuguesa de Estomatologia, Medicina Dentaria e Cirurgia Maxilofacial. 2016;57(1):1-8.	excluded/irrelevant	title
Jaeger R, Schmidt F, Naziris K, Lapatki BG. Evaluation of orthodontic loads and wire-bracket contact configurations in a three-bracket setup: Comparison of in-vitro experiments with numerical simulations. Journal of Biomechanics. 2021;121.	excluded/irrelevant	title
Damon DH. The rationale, evolution and clinical application of the self-ligating bracket. Clin Orthod Res. 1998;1(1):52-61.	excluded/irrelevant	title
Eliades T, Bourauel C. Intraoral aging of orthodontic materials: the picture we miss and its clinical relevance. Am J Orthod Dentofacial Orthop. 2005;127(4):403-12.	excluded/irrelevant	title
Voudouris JC, Schismenos C, Lackovic K, Kuftinec MM. Self-ligation esthetic brackets with low frictional resistance. Angle Orthod. 2010;80(1):188-94.	excluded/irrelevant	title
Bednar JR, Gruendeman GW. The influence of bracket design on moment production during axial rotation. Am J Orthod Dentofacial Orthop. 1993;104(3):254-61.	excluded/irrelevant	title
Barlow M, Kula K. Factors influencing efficiency of sliding mechanics to close extraction space: a systematic review. Orthod Craniofac Res. 2008;11(2):65-73.	excluded/irrelevant	title
Actrn. Change in salivary pH under the effect of stainless steel versus elastomeric ligatures in selected group of orthodontic patients. https://trialsearchwhoint/Trial2aspx?TrialID=ACTRN12618001647224. 2018.	excluded/ineligible outcome	full text
Thomas S, Sherriff M, Birnie D. A comparative in vitro study of the frictional characteristics of two types of self-ligating brackets and two types of pre-adjusted edgewise brackets tied with elastomeric ligatures. European Journal of Orthodontics. 1998;20(5):589-96.	excluded/irrelevant	title
Abels N, Backes CH, inventorsSelf-ligating orthodontic brackets including a metal ligation cover hingedly connected to a bracket base patent US 07210927. 2007 May 1 2007.	excluded/ineligible comparator	full text
Montasser MA, El-Bialy T, Keilig L, Reimann S, Jäger A, Bourauel C. Force levels in complex tooth alignment with conventional and self-ligating brackets. Am J Orthod Dentofacial Orthop. 2013;143(4):507-14.	excluded/irrelevant	title
Bazzini E, Bagatta S, Cattaneo S, Garattini G. Semipermanent replacement of missing teeth by orthodontic mini-implants, in growing patients, to maintain bone density: clinical case. Dental Cadmos. 2020;88(6):380-7.	excluded/irrelevant	title
Azevedo C, Forestier JP, Tavernier B. Effect of time on the flexural strength of glass ionomer and composite orthodontic adhesives. Angle Orthodontist. 2005;75(1):114-8.	excluded/irrelevant	title
Tiwari A, Sumathi Felicita A. Effectiveness of herbal mouthwash vs chlorhexidine mouthwash on the bacterial count in orthodontic patients. Plant Cell Biotechnology and Molecular Biology. 2020;21(33):1-5.	excluded/irrelevant	title
Almuzian M, McConnell E, Darendeliler MA, Alharbi F, Mohammed H. The effectiveness of alternating rapid maxillary expansion and constriction combined with maxillary protraction in the treatment of patients with a class III malocclusion: a systematic review and meta-analysis. Journal of orthodontics. 2018;45(4):250-9.	excluded/irrelevant	title
Liu Y, Guo HM. [Comparison of root resorption between self-ligating and conventional brackets using cone-beam CT]. Shanghai Kou Qiang Yi Xue. 2016;25(2):238-41.	excluded/irrelevant	title
Roy AS, Singh GK, Tandon P, Chaudhary R. Modified protrusion arch for anterior crossbite correction - a case report. Int J Orthod Milwaukee. 2013;24(4):41-3.	excluded/ irrelevant	title
Wright N, Modarai F, Cobourne MT, Dibiase AT. Do you do Damon®? What is the current evidence base underlying the philosophy of this appliance system? J Orthod. 2011;38(3):222-30.	excluded/ineligible comparator	full text
Banks P, Elton V, Jones Y, Rice P, Derwent S, Odondi L. The use of fixed appliances in the UK: a survey of specialist orthodontists. J Orthod. 2010;37(1):43-55.	excluded/irrelevant	title
Bergamo AZN, Casarin RCV, do Nascimento C, Matsumoto MAN, de Carvalho FK, da Silva RAB, et al. Self-ligating brackets exhibit accumulation of high levels of periodontopathogens in gingival crevicular fluid. Odontology. 2022;110(3):460-6.	excluded/ineligible comparator	full text
Bezerra Bandeira AM, Alves dos Santos MP, Pulitini G, Elias CN, da Costa MF. Influence of thermal or chemical degradation on the frictional force of an experimental coated NiTi wire. Angle Orthodontist. 2011;81(3):484-9.	excluded/irrelevant	title
Li H, Stocker T, Bamidis EP, Sabbagh H, Baumert U, Mertmann M, et al. Effect of different media on frictional forces between tribological systems made from self-ligating brackets in combination with different stainless steel wire dimensions. Dent Mater J. 2021;40(5):1250-6.	excluded/irrelevant	title
Wiltshire WA. In vitro and in vivo fluoride release from orthodontic elastomeric ligature ties. Am J Orthod Dentofacial Orthop. 1999;115(3):288-92.	excluded/irrelevant	title
Schumacher HA, Bourauel C, Drescher D. [The deactivation behavior and effectiveness of different orthodontic leveling arches--a dynamic analysis of the force systems]. Fortschr Kieferorthop. 1992;53(5):273-85.	excluded/duplicate	duplicate
Ferraz C, Castellucci M, Sobral M. Influence of in vitro pigmenting of esthetic orthodontic ligatures on smile attractiveness. Dental Press Journal of Orthodontics. 2012;17(5):123-30.	excluded/irrelevant	title
Savoldi F, Paganelli C. In vitro evaluation of loop design influencing the sliding of orthodontic wires: A preliminary study. Journal of Applied Biomaterials & Functional Materials. 2019;17(2).	excluded/irrelevant	title
Astl E, Onodera K, Celar A, Mitteroecker P, Bantleon H-P. Comparison of Conventional and Self-Ligating Brackets During Alignment. Informationen Aus Orthodontie Und Kieferorthopaedie. 2017;49(1):49-55.	excluded/irrelevant	title
Zhang A, Song Y, Cui X, Wang B, Li R. Application of in vivo traction-assisted resection of proximal colon lesions: a case series (with video). Surgical Endoscopy. 2022;36(11):8231-6.	excluded/irrelevant	title
George MG, Romanyk DL, George A, Li Y, Heo G, Major PW, et al. Comparison of third-order torque simulation with and without a periodontal ligament simulant. American Journal of Orthodontics and Dentofacial Orthopedics. 2015;148(3):431-9.	excluded/irrelevant	title
Kailasam V, Jagdish N, Chitharanjan AB. Self-ligation and faster treatment time--a myth? Am J Orthod Dentofacial Orthop. 2014;146(5):544-.	excluded/irrelevant	title
Li Y, Tang N, Xu Z, Feng X, Yang L, Zhao Z. Bidimensional techniques for stronger anterior torque control in extraction cases A combined clinical and typodont study. Angle Orthodontist. 2012;82(4):715-22.	excluded/irrelevant	title
Lin B, Jiang F, Chen J, Liang J. A Comparison of the Ligation Torque Expression of a Ribbonwise Bracket-Archwire Combination and a Conventional Combination: A Primary Study. Int J Clin Pract. 2022;2022:9251172.	excluded/irrelevant	title
Pliska BT, Fuchs RW, Beyer JP, Larson BE. Effect of applied moment on resistance to sliding among esthetic self-ligating brackets. Angle Orthod. 2014;84(1):134-9.	excluded/irrelevant	title

Kim K-R, Baek S-H. Effect of passive self-ligating bracket placement on the posterior teeth on reduction of frictional force in sliding mechanics. Korean Journal of Orthodontics. 2016;46(2):73-80.	excluded/irrelevant	title
Soma S, Iwamoto M, Higuchi Y, Kurisu K. Effects of continuous infusion of PTH on experimental tooth movement in rats. Journal of Bone and Mineral Research. 1999;14(4):546-54.	excluded/irrelevant	title
Islam ZU, Shaikh A, Fida M. Plaque index in multi-bracket fixed appliances. J Coll Physicians Surg Pak. 2014;24(11):791-5.	included	
Pithon MM, dos Santos RL, Pasini Judice RL, de Assuncao PS, Restle L. Evaluation of the cytotoxicity of elastomeric ligatures after sterilisation with 0.25% peracetic acid. Australian Orthodontic Journal. 2013;29(2):139-44.	excluded/irrelevant	title
Kuhlman DC, Lima TA, Duplat CB, Capelli JJ. Esthetic perception of orthodontic appliances by Brazilian children and adolescents. Dental Press J Orthod. 2016;21(5):58-66.	excluded/irrelevant	title
Sfondrini MF, Gatti S, Scribante A. Shear bond strength of self-ligating brackets. European Journal of Orthodontics. 2011;33(1):71-4.	excluded/irrelevant	title
Pandis N, Polychronopoulou A, Eliades T. Self-ligating vs conventional brackets in the treatment of mandibular crowding: a prospective clinical trial of treatment duration and dental effects. Am J Orthod Dentofacial Orthop. 2007;132(2):208-15.	excluded/irrelevant	title
Ioi H, Yanase Y, Uehara M, Hara A, Nakata S, Nakasima A, et al. Frictional resistance in plastic preadjusted brackets ligated with low-friction and conventional elastomeric ligatures. J Orthod. 2009;36(1):17-22; discussion 13.	excluded/irrelevant	abstract
Boyd RL. Clinical assessment of injuries in orthodontic movement of impacted teeth. I. Methods of attachment. Am J Orthod. 1982;82(6):478-86.	excluded/irrelevant	title
Papageorgiou SN, Konstantinidis I, Papadopoulou K, Jäger A, Bourauel C. Clinical effects of pre-adjusted edgewise orthodontic brackets: a systematic review and meta-analysis. Eur J Orthod. 2014;36(3):350-63.	excluded/irrelevant	title
Ctri. To compare the effect of two different methods of tying braces on the position of front and back teeth. https://trialsearchwhoint/Trial2aspx?TrialID=CTRI/2018/05/014220. 2018.	excluded/protocol	title
Durgekar SG, M S, Belludi A, K N. Quick and easy placement of coil spring. Int J Orthod Milwaukee. 2015;26(3):59-60.	excluded/irrelevant	title
Garino F, Favero L. Control of tooth movements with the Speed system. Prog Orthod. 2003;4:23-30.	excluded/irrelevant	title
Sifakakis I, Pandis N, Makou M, Eliades T, Bourauel C. A comparative assessment of the forces and moments generated at the maxillary incisors between conventional and self-ligating brackets using a reverse curve of Spee NiTi archwire. Aust Orthod J. 2010;26(2):127-33.	excluded/irrelevant	title
Morina E, Eliades T, Pandis N, Jaeger A, Bourauel C. Torque expression of self-ligating brackets compared with conventional metallic, ceramic, and plastic brackets. European Journal of Orthodontics. 2008;30(3):233-8.	excluded/irrelevant	title
Queiroz GV, Rino Neto J, De Paiva JB, Rossi JL, Ballester RY. Comparative study of classic friction among different archwire ligation systems. Dental Press Journal of Orthodontics. 2012;17(3):64-70.	excluded/irrelevant	title
Montasser MA, Keilig L, Bourauel C. Change in crown inclination accompanying initial tooth alignment with round archwires. Dental press journal of orthodontics. 2022;27(3):e2220489-e.	excluded/irrelevant	title
Major TW, Carey JP, Nobes DS, Heo G, Melenka GW, Major PW. An investigation into the mechanical characteristics of select self-ligated brackets at a series of clinically relevant maximum torquing angles: loading and unloading curves and bracket deformation. Eur J Orthod. 2013;35(6):719-29.	excluded/irrelevant	title
Atik E, Akarsu-Guven B, Kocadereli I, Ciger S. Evaluation of maxillary arch dimensional and inclination changes with self-ligating and conventional brackets using broad archwires. Am J Orthod Dentofacial Orthop. 2016;149(6):830-7.	excluded/irrelevant	abstract
Baghdadi D, Reimann S, Keilig L, Reichert C, Jäger A, Bourauel C. Biomechanical analysis of initial incisor crowding alignment in the periodontally reduced mandible using the finite element method. J Orofac Orthop. 2019;80(4):184-93.	excluded/irrelevant	title
Chhibber A, Agarwal S, Upadhyay M. Comparison of microbial colonization and periodontal status between conventional, self ligating and clear aligner therapy. A randomized controlled clinical trial. Journal of dental research. 2015;94(Spec Iss A).	excluded/ineligible comparator	full text
Fonseca AC, Pando MA. Peruvian adolescent preferences and acceptability of orthodontic fixed appliances. Revista Estomatologica Herediana. 2022;32(2):136-44.	excluded/irrelevant	title
Park KH, Han SJ, Choi S, Kim KS, Park S, Park JH. Surface Roughness on the Slots and Wings of Various Ceramic Self-Ligating Brackets and their Potential Concern on Biofilm Formation. J Clin Pediatr Dent. 2020;44(6):451-8.	excluded/irrelevant	title
Jacobs C, Jacobs-Müller C, Luley C, Erbe C, Wehrbein H. Orthodontic space closure after first molar extraction without skeletal anchorage. J Orofac Orthop. 2011;72(1):51-60.	excluded/irrelevant	title
Ong E, McCallum H, Griffin MP, Ho C. Efficiency of self-ligating vs conventionally ligated brackets during initial alignment. Am J Orthod Dentofacial Orthop. 2010;138(2):138.e1-7; discussion -9.	excluded/irrelevant	title
Sharma M, Sharma V, Khanna B. Mini-screw implant or transpalatal arch-mediated anchorage reinforcement during canine retraction: a randomized clinical trial. J Orthod. 2012;39(2):102-10.	excluded/irrelevant	title
Albertini P, Franciosi F, Palone M, Mollica F, Cremonini F. Comparative Analysis of Sliding Resistance of Different Lingual Systems. Pesquisa Brasileira em Odontopediatria e Clinica Integrada. 2021;21.	excluded/irrelevant	title
Contardo L, Perinetti G, Ceschi M, Vidoni G, Antoniolli F, Castaldo A, et al. Effects of deflection and archwire dimension on the mechanical performance of two self-ligating orthodontic systems. Prog Orthod. 2012;13(1):2-9.	excluded/irrelevant	title
Varella JR, Tomazini JE, Zangrandi Filho J. Avaliação de instrumentos para inserção de ligaduras elásticas em braquete ortodôntico. Rev paul odontol. 2011;33(2):23-7.	excluded/irrelevant	title
Gyawali R. An Effective Technique for Minor Rotation Correction. Int J Orthod Milwaukee. 2016;27(2):21-2.	excluded/irrelevant	title
Choi S, Hwang EY, Park HK, Park YG. Correlation between frictional force and surface roughness of orthodontic archwires. Scanning. 2015;37(6):399-405.	excluded/irrelevant	title
Sharma P, Valiathan A, Arora A, Agarwal S. A comparative evaluation of the retention of metallic brackets bonded with resin-modified glass ionomer cement under different enamel preparations: A pilot study. Contemporary clinical dentistry. 2013;4(2):140-6.	excluded/irrelevant	title
Lupi L, Paggetti H, Bertrand MF, Charavet C. [Biofilm and Orthodontic Materials: literature reviews and Scanning Electron Microscopy (SEM) images gallery]. Orthod Fr. 2022;93(2):111-23.	excluded/irrelevant	title
Fok J, Toogood RW, Badawi H, Carey JP, Major PW. Analysis of maxillary arch force/couple systems for a simulated high canine malocclusion: Part 2. Elastic ligation. Angle Orthod. 2011;81(6):960-5.	excluded/duplicate	duplicate
Downing A, McCabe JF, Gordon PH. The effect of artificial saliva on the frictional forces between orthodontic brackets and archwires. British journal of orthodontics. 1995;22(1):41-6.	excluded/duplicate	duplicate
Wiechmann D, Bantleon H-P, Melsen B, Zachrisson B, Hagg U, Canal P, et al. Incorrect measurements and misleading conclusions in the article "Comparison of the efficacy of tooth alignment among lingual and labial brackets: an in vitro study". Head & Face Medicine. 2020;16(1).	excluded/irrelevant	title
Bach RM. Self-ligation is not a scientific concept. Am J Orthod Dentofacial Orthop. 2009;136(6):757-8.	excluded/irrelevant	title
da Costa Monini A, Júnior LG, Martins RP, Vianna AP. Canine retraction and anchorage loss: self-ligating versus conventional brackets in a randomized split-mouth study. Angle Orthod. 2014;84(5):846-52.	excluded/irrelevant	title
Briseño-Marroquín B, López-Murillo H, Kuchen R, Casasa-Araujo A, Wolf TG. Pulp sensitivity changes during orthodontic treatment at different time periods: a prospective study. Clin Oral Investig. 2021;25(5):3207-15.	excluded/irrelevant	title
Nct. The Impact of Surgical Technique on PDC. https://clinicaltrialsgov/show/NCT02186548. 2014.	excluded/ irrelevant	title
Kumar V, Batra P, Sharma K, Raghavan S, Srivastava A. Comparative assessment of the rate of orthodontic tooth movement in adolescent patients undergoing treatment by first bicuspid extraction and en mass retraction, associated with low-frequency mechanical vibrations in passive self-ligating and conventional brackets: A randomized controlled trial. Int Orthod. 2020;18(4):696-705.	excluded/irrelevant	title
Brauchli LM, Steineck M, Wichelhaus A. Active and passive self-ligation: a myth? Part 1: torque control. Angle Orthod. 2012;82(4):663-9.	excluded/ineligible comparator	abstract
Yanase Y, Ioi H, Uehara M, Hara A, Nakata S, Nakasima A, et al. Comparison of the kinetic frictional force between conventional plastic brackets with thermoplastic low-friction module ligation and self-ligating brackets. World J Orthod. 2009;10(3):220-3.	excluded/ineligible comparator	full text
Garat JA, Martin AE, Gordillo ME, Ubios AM. Effect of orthodontic forces on root resorption in molars submitted to experimental periodontitis. Acta odontologica latinoamericana : AOL. 2004;17(1-2):3-7.	excluded/irrelevant	title
Lin H-p, Wu J-y. [A study of frictional resistance of archwires and ligating methods]. Shanghai kou qiang yi xue = Shanghai journal of stomatology. 2005;14(2):164-8.	excluded/ irrelevant	title
Syahdinda MR, Lucynda L, Triwardhani A, Hamid T. Comparison of frictional coefficient and surface roughness between three different active self-ligating brackets: An experimental in vitro study. Journal of International Oral Health. 2020;12(6):551-5.	excluded/irrelevant	title
Ustaoglu G, Korkmaz YN, Halicioglu K, Uysal O. Comparison of effects of bracket types and treatment duration on periodontal health of adult patients. Apos Trends in Orthodontics. 2019;9(2):94-8.	excluded/irrelevant	title
Cortesi R, Molinari L. A simple and efficient procedure for indirect bonding. Prog Orthod. 2010;11(2):180-4.	excluded/irrelevant	title
Yazicioglu S, Oz AA, Oz AZ, Arici N, Ozer M, Arici S. Buccolingual Inclination Effects of Self-Ligating and Conventional Premolar Brackets: A Cone Beam Computed Tomography Study. Turkish Journal of Orthodontics. 2020;33(2):110-4.	excluded/ineligible comparator	full text
Prettyman C, Best AM, Lindauer SJ, Tufekci E. Self-ligating vs conventional brackets as perceived by orthodontists. Angle Orthod. 2012;82(6):1060-6.	excluded/irrelevant	title
Fernandes DJ, Miguel JA, Quintão CC, Elias CN. Evaluation of frictional forces of polycarbonate self-ligating brackets. World J Orthod. 2010;11(3):250-5.	excluded/irrelevant	abstract
Karim Soltani M, Golfeshan F, Alizadeh Y, Mehrzad J. Resistance to Sliding in Clear and Metallic Damon 3 and Conventional Edgewise Brackets: an In vitro Study. J Dent (Shiraz). 2015;16(1 Suppl):15-20.	excluded/irrelevant	title
Aird JC, Durning P. Fracture of polycarbonate edgewise brackets: a clinical and SEM study. British journal of orthodontics. 1987;14(3):191-5.	excluded/irrelevant	title
Ogata RH, Nanda RS, Duncanson MG, Sinha PK, Currier GF. Frictional resistances in stainless steel bracket-wire combinations with effects of vertical deflections. Am J Orthod Dentofacial Orthop. 1996;109(5):535-42.	excluded/irrelevant	title
McLaughlin RP, Bennett JC. Evolution of treatment mechanics and contemporary appliance design in orthodontics: A 40-year perspective. Am J Orthod Dentofacial Orthop. 2015;147(6):654-62.	excluded/irrelevant	title
Lin C-Y, Kim JS, Lin ET, Lin ET. Prolonged water immersion alters resistance to sliding of aesthetic orthodontic coated wires. Orthodontics & Craniofacial Research. 2021;24(1):111-20.	excluded/irrelevant	title
Turner S, Harrison JE, Sharif FN, Owens D, Millett DT. Orthodontic treatment for crowded teeth in children. Cochrane Database Syst Rev. 2021;12(12):Cd003453.	excluded/irrelevant	title
Pringle AM, Petrie A, Cunningham SJ, McKnight M. Prospective randomized clinical trial to compare pain levels associated with 2 orthodontic fixed bracket systems. Am J Orthod Dentofacial Orthop. 2009;136(2):160-7.	excluded/irrelevant	title
Mazhari M, Moradinejad M, Mazhary M, Rekabi A, Rakhshan V. Effects of Rigid and Nonrigid Connections between the Miniscrew and Anchorage Tooth on Dynamics, Efficacy, and Adverse Effects of Maxillary Second Molar Protraction: A Finite Element Analysis. Biomed Research International. 2022;2022.	excluded/irrelevant	title
Ibiapina DJ, Oltramari-Navarro PV, Navarro RL, Almeida MR, Mendonça DL, Conti AC. Assessment of Dental Arch Changes and Buccal Bone Thickness in Patients treated with Self-ligating Brackets. J Contemp Dent Pract. 2016;17(6):434-9.	excluded/irrelevant	title
Marques IS, Araújo AM, Gurgel JA, Normando D. Debris, roughness and friction of stainless steel archwires following clinical use. Angle Orthod. 2010;80(3):521-7.	excluded/irrelevant	title
Ctri. Study procedures used for increasing height of small tooth. https://trialsearchwhoint/Trial2aspx?TrialID=CTRI/2019/11/022018. 2019.	excluded/ irrelevant	title
Khoshbin E, Soheilifar S, Donyavi Z, Shahsavand N. Evaluation of sensibility threshold of dental pulp to electric pulp test (EPT) in the teeth under fixed orthodontic treatment with 0.014 and 0.012 initial NiTi archwire. Journal of clinical and diagnostic research. 2019;13(1):ZC16-ZC9.	excluded/irrelevant	title
Diddige R, Negi G, Kiran KVS, Chitra P. Comparison of pain levels in patients treated with 3 different orthodontic appliances - a randomized trial. Medicine and pharmacy reports. 2020;93(1):81-8.	excluded/irrelevant	abstract
Actrn. A randomised prospective study of analgesic protocols for the management of orthodontic pain in relation to fitting of fixed appliances. https://trialsearchwhoint/Trial2aspx?TrialID=ACTRN12612000541808. 2012.	excluded/irrelevant	title

Fleming PS, Lee RT, Marinho V, Johal A. Comparison of maxillary arch dimensional changes with passive and active self-ligation and conventional brackets in the permanent dentition: a multicenter, randomized controlled trial. Am J Orthod Dentofacial Orthop. 2013;144(2):185-93.	excluded/irrelevant	abstract
Cavuoti S, Matarese G, Isola G, Abdolreza J, Femiano F, Perillo L. Combined orthodontic-surgical management of a transmigrated mandibular canine. Angle Orthod. 2016;86(4):681-91.	excluded/irrelevant	title
Bergamo AZN, Nelson-Filho P, Andrucioli MCD, do Nascimento C, Pedrazzi V, Matsumoto MAN. Microbial complexes levels in conventional and self-ligating brackets. Clin Oral Investig. 2017;21(4):1037-46.	excluded/ineligible comparator	full text
Nct. Effect of Mechanical Vibration on Root Resorption. https://clinicaltrialsgov/show/NCT04686617. 2020.	excluded/ irrelevant	title
Szczupakowski A, Reimann S, Dirk C, Keilig L, Weber A, Jäger A, et al. Friction behavior of self-ligating and conventional brackets with different ligature systems. J Orofac Orthop. 2016;77(4):287-95.	excluded/irrelevant	title
Ctri. MEASURING SALIVARY BACTERIAL COUNTS IN PATIENTS UNDERGOING ORTHODONTIC TREATMENT USING GREEN COFFEE BEAN MOUTHWASH. https://trialsearchwhoint/Trial2aspx?TrialID=CTRI/2021/09/036877. 2021.	excluded/ irrelevant	title
Arici N, Akdeniz BS, Arici S. Comparison of the frictional characteristics of aesthetic orthodontic brackets measured using a modified in vitro technique. Korean Journal of Orthodontics. 2015;45(1):29-37.	excluded/ineligible intervention	full text
Al-Ibrahim HM, Hajeer MY, Alkhouri I, Zinah E. Leveling and alignment time and the periodontal status in patients with severe upper crowding treated by corticotomy-assisted self-ligating brackets in comparison with conventional or self-ligating brackets only: a 3-arm randomized controlled clinical trial. J World Fed Orthod. 2022;11(1):3-11.	excluded/irrelevant	abstract
Hockstein NG, Samadi DS, Gendron K, Handler SD. Sialorrhea: a management challenge. Am Fam Physician. 2004;69(11):2628-34.	excluded/irrelevant	title
Prévost J, Granjon Y. An in vitro study of the passivity of splints in dental trauma. J Dent. 1998;26(1):39-45.	excluded/irrelevant	title
Türkkahraman H, Sayin MO, Bozkurt FY, Yetkin Z, Kaya S, Onal S. Archwire ligation techniques, microbial colonization, and periodontal status in orthodontically treated patients. Angle Orthod. 2005;75(2):231-6.	included	
Barragan G, Pereira R, Jardim L. Static and kinetic friction of self-ligating and conventional ceramic brackets. Revista Portuguesa De Estomatologia Medicina Dentaria E Cirurgia Maxilofacial. 2017;58(3):161-7.	excluded/irrelevant	title
Postlethwaite KM. Advances in fixed appliance design and use: 2. Auxiliaries, adhesives, appliance care and debonding. Dent Update. 1992;19(8):331-5.	excluded/irrelevant	title
Guimarães GS, de Morais LS, de Souza MM, Elias CN. Superficial morphology and mechanical properties of in vivo aged orthodontic ligatures. Dental Press J Orthod. 2013;18(3):107-12.	excluded/ineligible outcome	full text
Adenwalla ST, Attarzadeh F. The bonded mandibular lingual retainer. Br J Orthod. 1986;13(3):159-63.	excluded/irrelevant	title
Cha J-Y, Kim K-S, Hwang C-J. Friction of conventional and silica-insert ceramic brackets in various bracket-wire combinations. Angle Orthodontist. 2007;77(1):100-7.	excluded/irrelevant	title
Tanne K, Matsubara S, Hotei Y, Sakuda M, Yoshida M. Frictional forces and surface topography of a new ceramic bracket. Am J Orthod Dentofacial Orthop. 1994;106(3):273-8.	excluded/irrelevant	title
Kostopoulos L, Karring T. Susceptibility of GTR-regenerated periodontal attachment to ligature-induced periodontitis. J Clin Periodontol. 2004;31(5):336-40.	excluded/irrelevant	title
Kumar S, Singh S, Hamsa P R R, Ahmed S, Bhatnagar A, Sidhu M, et al. Evaluation of friction in orthodontics using various brackets and archwire combinations-an in vitro study. J Clin Diagn Res. 2014;8(5):ZC33-6.	excluded/irrelevant	title
Rock WP, Wilson HJ. The effect of bracket type and ligation method upon forces exerted by orthodontic archwires. British journal of orthodontics. 1989;16(3):213-7.	excluded/irrelevant	title
Galvão MB, Camporesi M, Tortamano A, Dominguez GC, Defraia E. Frictional resistance in monocrystalline ceramic brackets with conventional and nonconventional elastomeric ligatures. Prog Orthod. 2013;14:9.	excluded/irrelevant	title
Celli D, Catalfamo L, Gasperoni E, Deli R. A hybrid straightwire technique. Int Orthod. 2017;15(3):424-51.	excluded/irrelevant	title
Asensi JC. Mixed unilateral transposition of a maxillary canine, central incisor, and lateral incisor. Am J Orthod Dentofacial Orthop. 2010;137(4 Suppl):S141-53.	excluded/irrelevant	title
Shen H, Shao S, Zhang J, Wang Z, Lv D, Chen W, et al. Fixed orthodontic appliances cause pain and disturbance in somatosensory function. Eur J Oral Sci. 2016;124(1):26-32.	excluded/irrelevant	title
Fleming PS, DiBiase AT, Lee RT. Randomized clinical trial of orthodontic treatment efficiency with self-ligating and conventional fixed orthodontic appliances. Am J Orthod Dentofacial Orthop. 2010;137(6):738-42.	excluded/ineligible outcome	full text
Apiwantanakul N, Chantarawaratit PO. Cytotoxicity, genotoxicity, and cellular metal accumulation caused by professionally applied fluoride products in patients with fixed orthodontic appliances: A randomized clinical trial. Journal of the World Federation of Orthodontists. 2021;10(3):98-104.	excluded/irrelevant	abstract
Skaf Z, Nabbout F. Class II Correction with Microimplant Supported Molar Distalization: A Report of Two Cases. Case Reports in Dentistry. 2022;2022.	excluded/ irrelevant	title
Aznan K, Khan M, Benson PE. Gingival temperature measurements with fluoride and nonfluoride elastomeric ligatures. Am J Orthod Dentofacial Orthop. 2007;131(3):378-83.	excluded/irrelevant	abstract
Reznikov N, Har-Zion G, Barkana I, Abed Y, Redlich M. Measurement of friction forces between stainless steel wires and "reduced-friction" self-ligating brackets. Am J Orthod Dentofacial Orthop. 2010;138(3):330-8.	excluded/irrelevant	title
Chen W, Haq AA, Zhou Y. Root resorption of self-ligating and conventional preadjusted brackets in severe anterior crowding Class I patients: a longitudinal retrospective study. BMC Oral Health. 2015;15:115.	excluded/irrelevant	title
Popova NV, Arsenina OI, Popova AV, Gavrilova MV, Glukhova NV, Khvorostenko EA. Assessment of dental arches expansion using ligature and passive self-ligating braces in combination with Pitts broad arches and early elastics. Stomatologiia. 2021;100(4):55-62.	excluded/irrelevant	title
Loftus BP, Artum J. A model for evaluating friction during orthodontic tooth movement. Eur J Orthod. 2001;23(3):253-61.	excluded/irrelevant	title
Leander D, Kumar JK. Comparative evaluation of frictional characteristics of coated low friction ligatures - Super Slick Ties with conventional uncoated ligatures. Indian journal of dental research : official publication of Indian Society for Dental Research. 2011;22(1):90-4.	excluded/irrelevant	title
Condò R, Casaglia A, Condò SG, Cerroni L. Plaque retention on elastomeric ligatures. An in vivo study. Oral Implantol (Rome). 2012;5(4):92-9.	included	
Wong JK, Romanyk DL, Toogood RW, Heo G, Carey JP, Major PW. The effect of perturbations on resistance to sliding in second-order moments comparing two different bracket types. J Dent Biomech. 2014;5:1758736014557500-.	excluded/irrelevant	title
Nct. Microbial and Periodontal Changes Associated With Conventional Versus Self Ligating Brackets. https://clinicaltrialsgov/show/NCT02436525. 2015.	excluded/ irrelevant	title
Zhang MC, Ma XZ, Wu J, Zhao W, Sun HY, Zhao YS. Frictional resistance of archwires with various ligations in alignment phase. Chinese journal of tissue engineering research. 2018;22(18):2860-5.\	excluded/irrelevant	title
Ma XZ, Li HF, Zhao YH, Wu J, Zhang MC, Zhao W. Frictional force of different ligations with niti archwires of different sizes. Chinese Journal of Tissue Engineering Research. 2018;22(22):3491-7.	excluded/irrelevant	title
Gandini P, Orsi L, Bertoncini C, Massironi S, Franchi L. In vitro frictional forces generated by three different ligation methods. Angle Orthod. 2008;78(5):917-21.	excluded/irrelevant	title
Dimitriu T, Bolfa P, Suciu S, Cimpean A, Daradics Z, Catoi C, et al. Grape Seed Extract Reduces the Degree of Atherosclerosis in Ligature-Induced Periodontitis in Rats - An Experimental Study. J Med Life. 2020;13(4):580-6.	excluded/irrelevant	title
Ctri. Evaluation of rate of orthodontic tooth movement with and without low-intensity laser therapy. https://trialsearchwhoint/Trial2aspx?TrialID=CTRI/2022/07/044202. 2022.	excluded/ irrelevant	abstract
Cordasco G, Lo Giudice A, Militi A, Nucera R, Triolo G, Matarese G. In vitro evaluation of resistance to sliding in selfligating and conventional bracket systems during dental alignment. Korean Journal of Orthodontics. 2012;42(4):218-24.	excluded/irrelevant	title
Mittal R, Attri S, Batra P, Sonar S, Sharma K, Raghavan S. Comparison of orthodontic space closure using micro-osteoperforation and passive self-ligating appliances or conventional fixed appliances. Angle Orthod. 2020;90(5):634-9.	excluded/irrelevant	title
Reicheneder CA, Baumert U, Gedrange T, Proff P, Faltermeier A, Muessig D. Frictional properties of aesthetic brackets. European Journal of Orthodontics. 2007;29(4):359-65.	excluded/irrelevant	title
Burrow SJ. Friction and resistance to sliding in orthodontics: a critical review. Am J Orthod Dentofacial Orthop. 2009;135(4):442-7.	excluded/irrelevant	title
Major TW, Carey JP, Nobes DS, Heo G, Major PW. Mechanical effects of third-order movement in self-ligated brackets by the measurement of torque expression. Am J Orthod Dentofacial Orthop. 2011;139(1):e31-44.	excluded/irrelevant	title
Mester A, Onisor F, Mesaros AS. Periodontal Health in Patients with Self-Ligating Brackets: A Systematic Review of Clinical Studies. Journal of Clinical Medicine. 2022;11(9).	excluded/ineligible comparator	full text
Ferrari S, Bellincampi M, Sfondrini MF, Caprioglio A, Gandini P. Finishing effectiveness of different archwires using SmartClip™ self-ligating brackets: a clinical study. Int Orthod. 2014;12(1):125-38.	excluded/irrelevant	title
Heo W, Baek SH. Friction properties according to vertical and horizontal tooth displacement and bracket type during initial leveling and alignment. Angle Orthod. 2011;81(4):653-61.	excluded/irrelevant	title
Saporito I, Butti AC, Salvato A, Biagi R. A "typodont" study of rate of orthodontic space closure: self-ligating systems vs. conventional systems. Minerva stomatologica. 2011;60(11-12):555-65.	excluded/irrelevant	title
Olszewska A, Hanć A, Barałkiewicz D, Rzymski P. Metals and Metalloids Release from Orthodontic Elastomeric and Stainless Steel Ligatures: In Vitro Risk Assessment of Human Exposure. Biol Trace Elem Res. 2020;196(2):646-53.	excluded/irrelevant	title
Ai R, Li D, Shi L, Zhang X, Ding Z, Zhu Y, et al. Periodontitis induced by orthodontic wire ligature drives oral microflora dysbiosis and aggravates alveolar bone loss in an improved murine model. Front Microbiol. 2022;13:875091-.	excluded/irrelevant	title
Berger J, Byloff FK. The clinical efficiency of self-ligated brackets. J Clin Orthod. 2001;35(5):304-8.	excluded/irrelevant	title
Cacciafesta V, Sfondrini MF, Ricciardi A, Scribante A, Klersy C, Auricchio F. Evaluation of friction of stainless steel and esthetic self-ligating brackets in various bracket-archwire combinations. Am J Orthod Dentofacial Orthop. 2003;124(4):395-402.	excluded/ineligible outcome	abstract
Moyano J, Montagut D, Perera R, Fernández-Bozal J, Puigdollers A. Comparison of changes in the dental transverse and sagittal planes between patients treated with self-ligating and with conventional brackets. Dental Press J Orthod. 2020;25(1):47-55.	excluded/irrelevant	title
Miles PG. SmartClip versus conventional twin brackets for initial alignment: is there a difference? Aust Orthod J. 2005;21(2):123-7.	excluded/irrelevant	title
Chang C-J, Lee T-M, Liu J-K. Effect of bracket bevel design and oral environmental factors on frictional resistance. Angle Orthodontist. 2013;83(6):956-65.	excluded/irrelevant	title
Pandis N, Vlachopoulos K, Polychronopoulou A, Madianos P, Eliades T. Periodontal condition of the mandibular anterior dentition in patients with conventional and self-ligating brackets. Orthod Craniofac Res. 2008;11(4):211-5.	excluded/ineligible comparator	full text
Sakata S, Hallett KB, Brandon MS, McBride CA. Easy come, easy go: a simple and effective orthodontic enamel anchor for endotracheal tube stabilization in a child with extensive facial burns. Burns. 2009;35(7):983-6.	excluded/irrelevant	title
Zhang DP, Liu J, Liu Y, Sun N, Yi JC. [Influence of self-ligating and conventional brackets on dental arch width in non-extraction treatment: a meta analysis]. Shanghai Kou Qiang Yi Xue. 2014;23(3):367-72.	excluded/ineligible comparator	full text
Yang L, Yin G, Liao X, Yin X, Ye N. A novel customized ceramic bracket for esthetic orthodontics: in vitro study. Progress in Orthodontics. 2019;20(1).	excluded/irrelevant	title
Meling TR, Odegaard J, Holthe K, Segner D. The effect of friction on the bending stiffness of orthodontic beams: A theoretical and in vitro study. American Journal of Orthodontics and Dentofacial Orthopedics. 1997;112(1):41-9.	excluded/irrelevant	title
Kahlon S, Rinchuse D, Robison JM, Close JM. In-vitro evaluation of frictional resistance with 5 ligation methods and Gianelly-type working wires. American Journal of Orthodontics and Dentofacial Orthopedics. 2010;138(1):67-71.	excluded/irrelevant	title
Sahoo N, Kailasam V, Padmanabhan S, Chitharanjan AB. In-vivo evaluation of salivary nickel and chromium levels in conventional and self-ligating brackets. Am J Orthod Dentofacial Orthop. 2011;140(3):340-5.	excluded/irrelevant	title
Wang G, inventorOrthodontic self-locking bracket patent US 10092376. 2018 Oct 9 2018.	excluded/irrelevant	title
Al-Thomali Y, Mohamed RN, Basha S. Torque expression in self-ligating orthodontic brackets and conventionally ligated brackets: A systematic review. Journal of Clinical and Experimental Dentistry. 2017;9(1):e123-e8.	excluded/ irrelevant	title
Sebastian B, Bhuvaraghan A, Thiruvenkatachari B. Orthodontic space closure in sliding mechanics: a systematic review and meta-analysis. Eur J Orthod. 2022;44(2):210-25.	excluded/irrelevant	abstract
Tecco S, Teté S, Festa M, Festa F. An in vitro investigation on friction generated by ceramic brackets. World J Orthod. 2010;11(4):e133-44.	excluded/irrelevant	title
Sander FM, Sander C, Sander FG. Alignment of displaced or impacted teeth with the traction chain. J Orofac Orthop. 2006;67(4):289-96.	excluded/irrelevant	title
Mittal N, Xia Z, Chen J, Stewart KT, Liu SS-Y. Three-dimensional quantification of pretorqued nickel-titanium wires in edgewise and prescription brackets. Angle Orthodontist. 2013;83(3):484-90.	excluded/irrelevant	title

Sander CH, Sander FM, Sander FG. The behaviour of the periodontal ligament is influencing the use of new treatment tools. J Oral Rehabil. 2006;33(9):706-11.	excluded/irrelevant	title
Thorstenson GA, Kusy RP. Resistance to sliding of self-ligating brackets versus conventional stainless steel twin brackets with second-order angulation in the dry and wet (saliva) states. Am J Orthod Dentofacial Orthop. 2001;120(4):361-70.	excluded/irrelevant	title
Feres MFN, Vicioni-Marques F, Romano FL, Roscoe MG, Souza VMd, Torneri AL, et al. Streptococcus mutans adherence to conventional and self-ligating brackets: an in vitro study. Dental press journal of orthodontics. 2021;26(6):e212019-e.	excluded/ineligible comparator	full text
Liu X, Lin J, Ding P. Changes in the surface roughness and friction coefficient of orthodontic bracket slots before and after treatment. Scanning. 2013;35(4):265-72.	excluded/irrelevant	title
Vitale MC, Nardi MG, Pellegrini M, Spadari F, Pulicari F, Alcozer R, et al. Impacted Palatal Canines and Diode Laser Surgery: A Case Report. Case Reports in Dentistry. 2022;2022.	excluded/irrelevant	title
Sims AP, Waters NE, Birnie DJ. A comparison of the forces required to produce tooth movement ex vivo through three types of pre-adjusted brackets when subjected to determined tip or torque values. British journal of orthodontics. 1994;21(4):367-73.	excluded/irrelevant	abstract
Nuñez Alcántaro LN. Caso clínico tratado con brackets autoligantes activos. Actas odontol. 2015;12(2):32-40.	excluded/irrelevant	title
Ctri. Oral cell changes due to use of fluoride while undergoing orthodontic treatment. https://trialsearchwhoint/Trial2aspx?TrialID=CTRI/2022/04/042323. 2022.	excluded/ irrelevant	title
Yu YL, Qian YF. [The clinical implication of self-ligating brackets]. Shanghai Kou Qiang Yi Xue. 2007;16(4):431-5.	excluded/ineligible comparator	full text
Rudge P, Sherriff M, Bister D. A comparison of roughness parameters and friction coefficients of aesthetic archwires. Eur J Orthod. 2015;37(1):49-55.	excluded/irrelevant	title
Johnson G, Walker MP, Kula K. Fracture strength of ceramic bracket tie wings subjected to tension. Angle Orthodontist. 2005;75(1):95-100.	excluded/irrelevant	title
Nct. The Effect of Surgical Technique on PDC. https://clinicaltrialsgov/show/NCT05067712. 2021.	excluded/ irrelevant	title
Sifakakis I, Pandis N, Makou M, Eliades T, Katsaros C, Bourauel C. Torque efficiency of different archwires in 0.018-and 0.022-inch conventional brackets. Angle Orthodontist. 2014;84(1):149-54.	excluded/irrelevant	title
Flores-Mir C. No reliable evidence to guide initial arch wire choice for fixed appliance therapy. Evid Based Dent. 2013;14(4):114-5.	excluded/ irrelevant	title
Bergamo AZN, Matsumoto MAN, Nascimento CD, Andrucioli MCD, Romano FL, Silva RAB, et al. Microbial species associated with dental caries found in saliva and in situ after use of self-ligating and conventional brackets. J Appl Oral Sci. 2019;27:e20180426.	excluded/ineligible comparator	full text
Kusy RP, Whitley JQ, Prewitt MJ. Comparison of the Frictional Coefficients for Selected Archwire-Bracket Slot Combinations in the Dry and Wet States. Angle Orthodontist. 1991;61(4):293-302.	excluded/irrelevant	title
Ho CS, Ming Y, Foong KW, Rosa V, Thuyen T, Seneviratne CJ. Streptococcus mutans forms xylitol-resistant biofilm on excess adhesive flash in novel ex-vivo orthodontic bracket model. Am J Orthod Dentofacial Orthop. 2017;151(4):669-77.	excluded/ineligible comparator	full text
Grzegocka K, Krzyściak P, Hille-Padalis A, Loster JE, Talaga-Ćwiertnia K, Loster BW. Candida prevalence and oral hygiene due to orthodontic therapy with conventional brackets. BMC Oral Health. 2020;20(1):277.	excluded/irrelevant	
Defranco DJ, Spiller RE, Vonfraunhofer JA. Frictional Resistances Using Teflon-Coated Ligatures with Various Bracket Archwire Combinations. Angle Orthodontist. 1995;65(1):63-72.	excluded/irrelevant	abstract
Pires MSM, Reinhardt LC, Antonello GdM, Torres do Couto R. Use of orthodontic mini-implants for maxillomandibular fixation in mandibular fracture. Craniomaxillofacial trauma & reconstruction. 2011;4(4):213-6.	excluded/irrelevant	title
Migliorati M, Poggio D, Drago S, Lagazzo A, Stradi R, Barberis F, et al. Torque efficiency of a customized lingual appliance : Performance of wires with three different ligature systems. J Orofac Orthop. 2019;80(6):304-14.	excluded/irrelevant	title
Bousquet JA, Jr., Tuesta O, Flores-Mir C. In vivo comparison of force decay between injection molded and die-cut stamped elastomers. Am J Orthod Dentofacial Orthop. 2006;129(3):384-9.	excluded/irrelevant	title
Ctri. Comparing the efficiency of two different types of bracket system in correcting irregularly placed teeth without removal of teeth. https://trialsearchwhoint/Trial2aspx?TrialID=CTRI/2021/10/037227. 2021.	excluded/irrelevant	title
Zhang J, Qi S, Chen W, Liu L, Hui H. Evaluation of IL-1β and TNF-α expression and periodontitis under the influence of orthodontic appliances with minocycline. Cell Mol Biol (Noisy-le-grand). 2020;66(5):41-4.	excluded/ineligible comparator	full text
Rangarajan S, Mogra S, Shetty VS, Shetty S, Jose NP. Comparison of contamination of low-frictional elastomeric rings with that of conventional elastomeric rings by Streptococcus mutans - An in-vivo study. Journal of Clinical and Diagnostic Research. 2015;9(4):ZC26-ZC9.	excluded/comparator ineligible	abstract
Seru S, Romanyk DL, Toogood RW, Carey JP, Major PW. Effect of ligation method on maxillary arch force/moment systems for a simulated lingual incisor malalignment. Open Biomedical Engineering Journal. 2014;8:106-13.	excluded/irrelevant	title
Cardoso Mde A, Saraiva PP, Maltagliati L, Rhoden FK, Costa CC, Normando D, et al. Alterations in plaque accumulation and gingival inflammation promoted by treatment with self-ligating and conventional orthodontic brackets. Dental Press J Orthod. 2015;20(2):35-41.	excluded/ineligible comparator	abstract
Nct. Evaluation of Fixed Mandibular Retainer Using 3D Printed Positioning Tray Versus Direct Bonding Technique. https://clinicaltrialsgov/show/NCT03572179. 2018.	excluded/irrelevant	title
Franchi L, Baccetti T, Fortini A, Lupoli M. Differential friction in treatment with preadjusted fixed appliances. J Clin Orthod. 2008;42(9):504-7.	excluded/irrelevant	title
Aras I, Unal I, Huniler G, Aras A. Root resorption due to orthodontic treatment using self-ligating and conventional brackets : A cone-beam computed tomography study. J Orofac Orthop. 2018;79(3):181-90.	excluded/irrelevant	abstract
Youssef A, Dennis C, Beyer JP, Grunheid T. Resistance to sliding of orthodontic archwires under increasing applied moments. Journal of Applied Biomaterials & Functional Materials. 2020;18.	excluded/ineligible comparator	full text
Songra G, Clover M, Atack NE, Ewings P, Sherriff M, Sandy JR, et al. Comparative assessment of alignment efficiency and space closure of active and passive self-ligating vs conventional appliances in adolescents: a single-center randomized controlled trial. Am J Orthod Dentofacial Orthop. 2014;145(5):569-78.	excluded/irrelevant	abstract
Gupta S, Singh G, Kannan S, Singh RK, Goyal V, Gupta N. Comparing bite force in untreated and orthodontically treated non- extraction cases with MBT appliance and self ligation technique using T-scan. J Oral Biol Craniofac Res. 2022;12(1):187-91.	excluded/irrelevant	title
Albertini P, Mazzanti V, Mollica F, Lombardo L, Siciliani G. Comparative analysis of passive play and torque expression in self-ligating and traditional lingual brackets. Journal of Orofacial Orthopedics-Fortschritte Der Kieferorthopadie. 2022;83(1):13-22.	excluded/ ineligible outcome	abstract
Maizeray R, Wagner D, Lefebvre F, Lévy-Bénichou H, Bolender Y. Is there any difference between conventional, passive and active self-ligating brackets? A systematic review and network meta-analysis. Int Orthod. 2021;19(4):523-38.	excluded/irrelevant	title
Thorstenson GA, Kusy RP. Comparison of resistance to sliding between different self-ligating brackets with second-order angulation in the dry and saliva states. Am J Orthod Dentofacial Orthop. 2002;121(5):472-82.	excluded/irrelevant	title
Dixon V, Read MJ, O'Brien KD, Worthington HV, Mandall NA. A randomized clinical trial to compare three methods of orthodontic space closure. Journal of orthodontics. 2002;29(1):31-6.	excluded/irrelevant	title
Alabdullah MM, Burhan AS, Nabawia A, Nawaya F, Saltaji H. Comparative assessment of dental and basal arch dimensions of passive and active self-ligating versus conventional appliances A randomized clinical trial. Journal of Orofacial Orthopedics-Fortschritte Der Kieferorthopadie. 2022.	excluded/irrelevant	title
Paquette DE. Biased look at self-ligation. Am J Orthod Dentofacial Orthop. 2011;139(5):574; author reply -5.	excluded/irrelevant	title
Shivapuja PK, Berger J. A Comparative-Study of Conventional Ligation and Self-Ligation Bracket Systems. American Journal of Orthodontics and Dentofacial Orthopedics. 1994;106(5):472-80.	excluded/duplicate	duplicate
Odegaard J, Meling E, Meling T. An Evaluation of the Torsional Moments Developed in Orthodontic Applications - an in-Vitro Study. American Journal of Orthodontics and Dentofacial Orthopedics. 1994;105(4):392-400.	excluded/irrelevant	title
Pandis N, Polychronopoulou A, Makou M, Eliades T. Mandibular dental arch changes associated with treatment of crowding using self-ligating and conventional brackets. Eur J Orthod. 2010;32(3):248-53.	excluded/irrelevant	title
Elayyan F, Silikas N, Bearn D. Ex vivo surface and mechanical properties of coated orthodontic archwires. Eur J Orthod. 2008;30(6):661-7.	excluded/irrelevant	title
Henao SP, Kusy RP. Evaluation of the frictional resistance of conventional and self-ligating bracket designs using standardized archwires and dental typodonts. Angle Orthod. 2004;74(2):202-11.	excluded/irrelevant	title
Dagdeviren C, Gulec A, Eksi F, Saglam M, Kahraman M. Contamination of Low Frictional Elastomeric Ligatures by Streptococcus mutans: A Prospective RT-PCR and AFM Study. Turkish Journal of Orthodontics. 2021;34(3):163-9.	excluded/irrelevant	full text
Nct. Alignment Efficiency and Coating Durability of Aesthetic Archwires. https://clinicaltrialsgov/show/NCT03876184. 2019.	excluded/ irrelevant	title
Verma P, Jain RK. Comparative evaluation of stability of mandibular anterior crowding correction done with two different treatment protocols: A retrospective study. Journal of International Oral Health. 2022;14(2):189-94.	excluded/irrelevant	title
Zhou Q, Ul Haq AA, Tian L, Chen X, Huang K, Zhou Y. Canine retraction and anchorage loss self-ligating versus conventional brackets: a systematic review and meta-analysis. BMC Oral Health. 2015;15(1):136.	excluded/irrelevant	title
Kao C-T, Ding S-J, Wang C-K, He H, Chou M-Y, Huang T-H. Comparison of frictional resistance after immersion of metal brackets and orthodontic wires in a fluoride-containing prophylactic agent. American journal of orthodontics and dentofacial orthopedics : official publication of the American Association of Orthodontists, its constituent societies, and the American Board of Orthodontics. 2006;130(5):568.e1-9.	excluded/irrelevant	title
Al Fakir H, Carey JP, Melenka GW, Nobes DS, Heo G, Major PW. Investigation into the effects of stainless steel ligature ties on the mechanical characteristics of conventional and self-ligated brackets subjected to torque. J Orthod. 2014;41(3):188-200.	excluded/ineligible outcome	abstract
Berger J. The engaging concept of self-ligation. Ont Dent. 1999;76(3):26-33.	excluded/irrelevant	title
Capistrano A, Cordeiro A, Siqueira DF, Capelozza Filho L, Cardoso Mde A, Almeida-Pedrin RR. From conventional to self-ligating bracket systems: is it possible to aggregate the experience with the former to the use of the latter? Dental Press J Orthod. 2014;19(3):139-57.	excluded/irrelevant	title
Kim T-W, 최원철, 박주영, 곽재혁, 나효정, 박두남. The effect of bracket width on frictional force between bracket and arch wire during sliding tooth movement. The Korean Journal of Orthodontics. 2004;34(3):253-60.	excluded/irrelevant	title
Kim Y, Cha J-Y, Hwang C-J, Yu HS, Tahk SG. Comparison of frictional forces between aesthetic orthodontic coated wires and self-ligation brackets. Korean J Orthod. 2014;44(4):157-67.	excluded/irrelevant	title
Sawhney R, Sharma R, Sharma K. Microbial Colonization on Elastomeric Ligatures during Orthodontic Therapeutics: An Overview. Turkish Journal of Orthodontics. 2018;31(1):21-5.	excluded/review	title
Sanders E, Johannessen L, Nadal J, Jaeger A, Bourauell C. Comparison of multiforce nickel-titanium wires to multistrand wires without force zones in bending and torque measurements. Journal of Orofacial Orthopedics-Fortschritte Der Kieferorthopadie. 2022;83(6):382-94.	excluded/irrelevant	title
Bertl MH, Onodera K, Čelar AG. A prospective randomized split-mouth study on pain experience during chairside archwire manipulation in self-ligating and conventional brackets. Angle Orthod. 2013;83(2):292-7.	excluded/irrelevant	abstract
Meazzini MC, Torre C, Cappello A, Tintinelli R, De Ponti E, Mazzoleni F. Long-term follow-up of late maxillary orthopedic advancement with the Liou-Alternate rapid maxillary expansion-constriction technique in patients with skeletal Class III malocclusion. American journal of orthodontics and dentofacial orthopedics : official publication of the American Association of Orthodontists, its constituent societies, and the American Board of Orthodontics. 2021;160(2):221-30.	excluded/irrelevant	title
Nct. Photobiomodulation on Orthodontic Movement of Molar Verticalization With Mini-implant. https://clinicaltrialsgov/show/NCT04036539. 2019.	excluded/ irrelevant	title
Daratsianos N, Bourauel C, Fimmers R, Jäger A, Schwestka-Polly R. In vitro biomechanical analysis of torque capabilities of various 0.018″ lingual bracket-wire systems: total torque play and slot size. Eur J Orthod. 2016;38(5):459-69.	excluded/irrelevant	title
Rahman S, Spencer RJ, Littlewood SJ, O'Dywer L, Barber SK, Russell JS. A multicenter randomized controlled trial to compare a self-ligating bracket with a conventional bracket in a UK population: Part 2: Pain perception. Angle Orthod. 2016;86(1):149-56.	excluded/irrelevant	abstract
Melsen B, Agerbaek N, Eriksen J, Terp S. New Attachment through Periodontal Treatment and Orthodontic Intrusion. American Journal of Orthodontics and Dentofacial Orthopedics. 1988;94(2):104-16.	excluded/irrelevant	abstract
Griffiths HS, Sherriff M, Ireland AJ. Resistance to sliding with 3 types of elastomeric modules. Am J Orthod Dentofacial Orthop. 2005;127(6):670-5; quiz 754.	excluded/irrelevant	title

Miles P, Weyant R. Porcelain brackets during initial alignment: are self-ligating cosmetic brackets more efficient? Aust Orthod J. 2010;26(1):21-6.	excluded/irrelevant	title
Quadrelli C, Veneziani A. The Stealth in comparison with other lingual brackets: properties and procedures for indirect bonding. Prog Orthod. 2007;8(1):156-72.	excluded/irrelevant	title
Fleming PS, DiBiase AT, Lee RT. Self-ligating appliances: evolution or revolution? Aust Orthod J. 2008;24(1):41-9.	excluded/irrelevant	title
Flores DA, Caruso JM, Scott GE, Jeiroudi MT. The fracture strength of ceramic brackets: a comparative study. Angle Orthod. 1990;60(4):269-76.	excluded/irrelevant	title
Yu Z, RongDang H. Comparison of self- and conventional-ligating brackets in the alignment stage. Eur J Orthod. 2013;35(1):139.	excluded/ineligible comparator	full text
Suresh N, Naveen Kumar M. Comparison of initial alignment between self ligation and conventional ligation systems for decrowding of mandibular anterior teeth. International Journal of Dentistry and Oral Science. 2020;7(10):918-22.	excluded/irrelevant	title
Nyman S, Houston F, Sarhed G, Lindhe J, Karring T. Healing Following Reimplantation of Teeth Subjected to Root Planing and Citric-Acid Treatment. Journal of Clinical Periodontology. 1985;12(4):294-305.	excluded/irrelevant	title
Dalessandri D, Lazzaroni E, Migliorati M, Piancino MG, Tonni I, Bonetti S. Self-ligating fully customized lingual appliance and chair-time reduction: a typodont study followed by a randomized clinical trial. Eur J Orthod. 2013;35(6):758-65.	excluded/irrelevant	title
Jang T-H, Kim S-C, Cho J-H, Chae J-M, Chang N-Y, Kang KH. The comparison of the frictional force by the type and angle of orthodontic bracket and the coated or non-coated feature of archwire. Korean Journal of Orthodontics. 2011;41(6):399-410.	excluded/irrelevant	title
Sueri MY, Turk T. Effectiveness of laceback ligatures on maxillary canine retraction. Angle Orthod. 2006;76(6):1010-4.	excluded/irrelevant	title
Nayak RS, Shafiuddin B, Pasha A, Vinay K, Narayan A, Shetty SV. Comparison of Galvanic Currents Generated Between Different Combinations of Orthodontic Brackets and Archwires Using Potentiostat: An In Vitro Study. J Int Oral Health. 2015;7(7):29-35.	excluded/irrelevant	title
Pandis N, Nasika M, Polychronopoulou A, Eliades T. External apical root resorption in patients treated with conventional and self-ligating brackets. Am J Orthod Dentofacial Orthop. 2008;134(5):646-51.	excluded/irrelevant	title
Romanyk DL, George A, Li Y, Heo G, Carey JP, Major PW. Influence of second-order bracket-archwire misalignments on loads generated during third-order archwire rotation in orthodontic treatment. Angle Orthodontist. 2016;86(3):358-64.	excluded/irrelevant	title
Iwasaki LR, Beatty MW, Nickel JC. Friction and orthodontic mechanics: Clinical studies of moment and ligation effects. Seminars in Orthodontics. 2003;9(4):290-7.	excluded/irrelevant	title
Perrey W, Konermann A, Keilig L, Reimann S, Jaeger A, Bourauel C. Effect of archwire qualities and bracket designs on the force systems during leveling of malaligned teeth. Journal of Orofacial Orthopedics-Fortschritte Der Kieferorthopadie. 2015;76(2):129-42.	excluded/irrelevant	title
Sodor A, Zegan G, Golovcencu L, Anistoroaei D. The Cytotoxicity of Orthodontic Polymeric Biomaterials. Romanian Journal of Oral Rehabilitation. 2018;10(3):176-82.	excluded/irrelevant	title
Morrier JJ. [White spot lesions and orthodontic treatment. Prevention and treatment]. Orthod Fr. 2014;85(3):235-44.	excluded/irrelevant	title
Murakami-Malaquias-Silva F, Rosa EP, Almeida PA, Schalch TO, Tenis CA, Negreiros RM, et al. Evaluation of the effects of photobiomodulation on orthodontic movement of molar verticalization with mini-implant: A randomized double-blind protocol study. Medicine (Baltimore). 2020;99(13):e19430.	excluded/irrelevant	title
Franco É M, Valarelli FP, Fernandes JB, Cançado RH, de Freitas KM. Comparative study of torque expression among active and passive self-ligating and conventional brackets. Dental Press J Orthod. 2015;20(6):68-74.	excluded/irrelevant	title
Ctri. To check the effect of laser treatment on the rate of orthodontic tooth movement using two types of orthodontic brackets-specialized and normal brackets. https://trialsearchwhoint/Trial2aspx?TrialID=CTRI/2018/04/013156. 2018.	excluded/ irrelevant	title
Baart JA, Weststrate CM, Bosgra JFL. [Surgical-orthodontic treatment of an impacted maxillary incisor]. Ned Tijdschr Tandheelkd. 2008;115(8):439-41.	excluded/irrelevant	title
Franchi L, Baccetti T, Camporesi M, Giuntini V. Forces released by nonconventional bracket or ligature systems during alignment of buccally displaced teeth. Am J Orthod Dentofacial Orthop. 2009;136(3):316.e1-6; discussion -7.	excluded/irrelevant	title
Zuñiga-Heredia EE, Muguruma T, Kawamura N, Iijima M. Frictional Forces of Three Types of Lingual Appliance with Self-ligating Mechanisms. J Contemp Dent Pract. 2021;22(6):605-9.	excluded/irrelevant	abstract
Maurya RK, Bhardwaj P, Singh H, Mishra HA. Comparative evaluation of duration of extraction space closure and degree of root resorption with conventional and self-Ligation brackets. Indian J Dent Res. 2022;33(1):52-7.	excluded/irrelevant	title
Wagner L. [Alignment of malpositioned canines]. Prakt Kieferorthop. 1991;5(1):29-36.	excluded/ineligible intervention	full text
Barbosa JA, Elias CN, Basting RT. Evaluation of friction produced by self-ligating, conventional and Barbosa Versatile brackets Barbosa Versátil. Revista de Odontologia da UNESP. 2016;45(2):71-7.	excluded/irrelevant	title
St George G, Donachie MA. Case report: orthodontic separators as periodontal ligatures in periodontal bone loss. The European journal of prosthodontics and restorative dentistry. 2002;10(3):97-9.	excluded/duplicate	duplicate
Alam MK. Laser-Assisted Orthodontic Tooth Movement in Saudi Population: A Prospective Clinical Intervention of Low-Level Laser Therapy in the 1st Week of Pain Perception in Four Treatment Modalities. Pain Res Manag. 2019;2019:6271835.	excluded/duplicate	duplicate
Actrn. The effects of a clinically feasible application of low-level laser therapy on the rate of orthodontic tooth movement: a triple-blinded randomized controlled clinical trial. https://trialsearchwhoint/Trial2aspx?TrialID=ACTRN12619001237178. 2019.	excluded/irrelevant	title
Lima VNC, Coimbra MER, Derech CDA, Ruellas ACdO. A força de atrito em braquetes plásticos e de aço inoxidável com a utilização de quatro diferentes tipos de amarração. Dental press j orthod (Impr). 2010;15(2):82-6.	excluded/irrelevant	title
Qin F, Zhou Y. The influence of bracket type on the external apical root resorption in class I extraction patients - a retrospective study. BMC Oral Health. 2019;19(1):53.	excluded/irrelevant	title
Chung M, Nikolai RJ, Kim KB, Oliver DR. Third-Order Torque and Self-Ligating Orthodontic Bracket-Type Effects on Sliding Friction. Angle Orthodontist. 2009;79(3):551-7.	excluded/duplicate	duplicate
Sobral GC, Vedovello Filho M, Degan VV, Santamaria M. Photoelastic analysis of stress generated by wires when conventional and self-ligating brackets are used: a pilot study. Dental press journal of orthodontics. 2014;19(5):74-8.	excluded/irrelevant	title
Henao SP, Kusy RP. Frictional evaluations of dental typodont models using four self-ligating designs and a conventional design. Angle Orthod. 2005;75(1):75-85.	excluded/irrelevant	title
Kostopoulos L, Karring T. Susceptibility of GTR-regenerated periodontal attachment to ligature-induced periodontitis: An experiment in the monkey. Journal of Clinical Periodontology. 2004;31(5):336-40.	excluded/duplicate	duplicate
Shi J, Liu Z, Kawai T, Zhou Y, Han X. Antibiotic administration alleviates the aggravating effect of orthodontic force on ligature-induced experimental periodontitis bone loss in mice. J Periodontal Res. 2017;52(4):725-33.	excluded/irrelevant	title
do Nascimento LE, Pithon MM, dos Santos RL, Freitas AO, Alviano DS, Nojima LI, et al. Colonization of Streptococcus mutans on esthetic brackets: self-ligating vs conventional. Am J Orthod Dentofacial Orthop. 2013;143(4 Suppl):S72-7.	excluded/ineligible comparatore	full text
Choi S, Joo HJ, Cheong Y, Park YG, Park HK. Effects of self-ligating brackets on the surfaces of stainless steel wires following clinical use: AFM investigation. J Microsc. 2012;246(1):53-9.	excluded/irrelevant	title
Huang J, Li CY, Jiang JH. Effects of fixed orthodontic brackets on oral malodor: A systematic review and meta-analysis according to the preferred reporting items for systematic reviews and meta-analyses guidelines. Medicine (Baltimore). 2018;97(14):e0233.	excluded/irrelevant	title
Feu D, Catharino F, Duplat CB, Capelli Junior J. Esthetic perception and economic value of orthodontic appliances by lay Brazilian adults. Dental Press Journal of Orthodontics. 2012;17(5):102-14.	excluded/irrelevant	title
Arnold S, Koletsi D, Patcas R, Eliades T. The effect of bracket ligation on the periodontal status of adolescents undergoing orthodontic treatment. A systematic review and meta-analysis. J Dent. 2016;54:13-24.	excluded/review	title
Baka ZM, Basciftci FA, Arslan U. Effects of 2 bracket and ligation types on plaque retention: a quantitative microbiologic analysis with real-time polymerase chain reaction. Am J Orthod Dentofacial Orthop. 2013;144(2):260-7.	excluded/ineligible comparator	full text
Southard TE, Marshall SD, Grosland NM. Friction does not increase anchorage loading. Am J Orthod Dentofacial Orthop. 2007;131(3):412-4.	excluded/irrelevant	title
Wang Z, Wang Y, Yan Y, Zeng L. Effect of self-ligating brackets on periodontal tissues and inflammatory factors in patients with chronic periodontitis undergoing orthodontic treatment. International journal of clinical and experimental medicine. 2021;14(2):1391-6.	excluded/ineligible comparator	full text
Sims AP, Waters NE, Birnie DJ, Pethybridge RJ. A comparison of the forces required to produce tooth movement in vitro using two self-ligating brackets and a pre-adjusted bracket employing two types of ligation. Eur J Orthod. 1993;15(5):377-85.	excluded/irrelevant	abstract
Crawford NL, McCarthy C, Murphy TC, Benson PE. Physical properties of conventional and Super Slick elastomeric ligatures after intraoral use. Angle Orthod. 2010;80(1):175-81.	excluded/irrelevant	title
Montasser MA, Keilig L, Bourauel C. An in vitro study into the efficacy of complex tooth alignment with conventional and self-ligating brackets. Orthod Craniofac Res. 2015;18(1):33-42.	excluded/irrelevant	title
Mitra R, Londhe SM, Kumar P. A comparative evaluation of rate of space closure after extraction using E-chain and stretched modules in bimaxillary dentoalveolar protrusion cases. Medical Journal Armed Forces India. 2011;67(2):152-6.	excluded/irrelevant	title
Manea A, Dinu C, Băciuţ M, Buduru S, Almăşan O. Intrusion of Maxillary Posterior Teeth by Skeletal Anchorage: A Systematic Review and Case Report with Thin Alveolar Biotype. Journal of Clinical Medicine. 2022;11(13).	excluded/irrelevant	title
Ctri. Clinical study to evaluate Pain perception after Interproximal reduction Using Neuromodulation Device. https://trialsearchwhoint/Trial2aspx?TrialID=CTRI/2022/10/046373. 2022.	excluded/irrelevant	title
Actrn. Efficacy of Modified Piezocision Corticotomy Procedure to Accelerate Orthodontic Tooth Movement. https://trialsearchwhoint/Trial2aspx?TrialID=ACTRN12621001350819. 2021.	excluded/irrelevant	title
Curiel P, Salah P, Ayache W, inventors; H, assignee. Assembly formed by a self-ligating bracket and an elastic clip, for an orthodontic apparatus patent US 09498303. 2016 Nov 22 2016.	excluded/irrelevant	title
Lee GJ, Park KH, Park YG, Park HK. A quantitative AFM analysis of nano-scale surface roughness in various orthodontic brackets. Micron. 2010;41(7):775-82.	excluded/irrelevant	title
Wichelhaus A, Geserick M, Hibst R, Sander FG. The effect of surface treatment and clinical use on friction in NiTi orthodontic wires. Dent Mater. 2005;21(10):938-45.	excluded/irrelevant	title
Reicheneder CA, Gedrange T, Berrisch S, Proff P, Baumert U, Faltermeier A, et al. Conventionally ligated versus self-ligating metal brackets--a comparative study. Eur J Orthod. 2008;30(6):654-60.	excluded/irrelevant	title
Bindayel NA, Alwadei A, Almosa N, Aasser W, Qazali A, Samran A, et al. Evaluation of bracket failure in relation to different factors in patients experiencing comprehensive orthodontic treatment: a retrospective cohort study. J oral res (Impresa). 2019;8(2):116-21.	excluded/irrelevant	title
Nct. The Validity of Customized Orthodontic Bracket Set up. https://clinicaltrialsgov/show/NCT05549089. 2022.	excluded/ irrelevant	title
Yang X, Su N, Shi Z, Xiang Z, He Y, Han X, et al. Effects of self-ligating brackets on oral hygiene and discomfort: a systematic review and meta-analysis of randomized controlled clinical trials. Int J Dent Hyg. 2017;15(1):16-22.	excluded/Review	abstract
Jacobs C, Gebhardt PF, Jacobs V, Hechtner M, Meila D, Wehrbein H. Root resorption, treatment time and extraction rate during orthodontic treatment with self-ligating and conventional brackets. Head Face Med. 2014;10:2.	excluded/irrelevant	title
Morina E, Keilig L, Jaeger A, Bourauel C. Biomechanical analysis of orthodontic brackets with different closing mechanisms. Biomedizinische Technik. 2009;54(2):89-97.	excluded/irrelevant	title
Moradinejad M, Ghorani N, Heidarpour M, Noori M, Rakhshan V. Effects of a ceramic active self-ligating bracket on retraction/tipping/rotation of canine, premolar mesialization, and transverse arch dimensions: A preliminary single-blind split-mouth randomized clinical trial. Dental Research Journal. 2021;18(1).	excluded/irrelevant	title
Baccetti T, Franchi L, Fortini A. Orthodontic treatment with preadjusted appliances and low-friction ligatures: experimental evidence and clinical observations. World J Orthod. 2008;9(1):7-13.	excluded/irrelevant	title
Turner S, Harrison JE, Sharif FNJ, Owens D, Millett DT. Orthodontic treatment for crowded teeth in children. Cochrane Database of Systematic Reviews. 2021(12).	excluded/irrelevant	title
Vinay K, Venkatesh MJ, Nayak RS, Pasha A, Rajesh M, Kumar P. A comparative study to evaluate the effects of ligation methods on friction in sliding mechanics using 0.022" slot brackets in dry state: An In-vitro study. J Int Oral Health. 2014;6(2):76-83.	excluded/irrelevant	title
Curiel P, Salah P, Ayache W, inventors; American Orthodontics Corporation, assignee. Clip-base-bracket assembly having a base for an orthodontic apparatus and orthodontic apparatus comprising same patent US 10226311. 2019 Mar 12 2019.	excluded/irrelevant	title

Miles PG. Self-ligating brackets in orthodontics: Do they deliver what they claim? Aust Dent J. 2009;54(1):9-11.	excluded/irrelevant	title
Khambay B, Millett D, McHugh S. Archwire seating forces produced by different ligation methods and their effect on frictional resistance. Eur J Orthod. 2005;27(3):302-8.	excluded/irrelevant	title
Bashir R, Sonar S, Batra P, Srivastava A, Singla A. Comparison of transverse maxillary dental arch width changes with self-ligating and conventional brackets in patients requiring premolar extraction - A randomised clinical trial. Int Orthod. 2019;17(4):687-92.	excluded/irrelevant	title
Fok J, Toogood RW, Badawi H, Carey JP, Major PW. Analysis of maxillary arch force/couple systems for a simulated high canine malocclusion: Part 1. Passive ligation. Angle Orthod. 2011;81(6):953-9.	excluded/irrelevant	title
Yamaguchi K, Nanda RS, Morimoto N, Oda Y. A study of force application, amount of retarding force, and bracket width in sliding mechanics. Am J Orthod Dentofacial Orthop. 1996;109(1):50-6.	excluded/irrelevant	title
da Costa Monini A, Júnior LGG, Vianna AP, Martins RP. A comparison of lower canine retraction and loss of anchorage between conventional and self-ligating brackets: a single-center randomized split-mouth controlled trial. Clin Oral Investig. 2017;21(4):1047-53.	excluded/irrelevant	abstract
Pandis N, Polychronopoulou A, Eliades T. Failure rate of self-ligating and edgewise brackets bonded with conventional acid etching and a self-etching primer: a prospective in vivo study. Angle Orthod. 2006;76(1):119-22.	excluded/irrelevant	title
Dong L, Xu P. Danzhi Jiangtang capsule alleviate hyperglycemia and periodontitis via Wnt/ß-catenin signaling in diabetic rat. J Tradit Chin Med. 2021;41(4):608-16.	excluded/irrelevant	title
Yamaguchi M, Takizawa T, Nakajima R, Imamura R, Kasai K. The Damon System and release of substance P in gingival crevicular fluid during orthodontic tooth movement in adults. World J Orthod. 2009;10(2):141-6.	excluded/ irrelevant	title
Alves de Souza R, Borges de Araújo Magnani MB, Nouer DF, Oliveira da Silva C, Klein MI, Sallum EA, et al. Periodontal and microbiologic evaluation of 2 methods of archwire ligation: ligature wires and elastomeric rings. Am J Orthod Dentofacial Orthop. 2008;134(4):506-12.	included	
Mateu ME, Benítez-Rogé S, Iglesias M, Calabrese D, Lumi M, Solla M, et al. Increased interpremolar development with self-ligating orthodontics. A prospective randomized clinical trial. Acta Odontol Latinoam. 2018;31(2):104-9.	excluded/irrelevant	title
Polat Ö, Gökçelik A, Arman A, Arhun N. A comparison of white spot lesion formation between a self-ligating bracket and a conventional preadjusted straight wire bracket. World J Orthod. 2008;9(2):e46-50.	excluded/irrelevant	title
Mendes K, Rossouw PE. Friction: Validation of manufacturer's claim. Seminars in Orthodontics. 2003;9(4):236-50.	excluded/irrelevant	title
Fertik SM. Periodontal surgical management of impacted maxillary cuspids. R I Dent J. 1995;28(2):5-7.	excluded/irrelevant	title
Radhakrishnan PD, Varma NKS, Ajith VV. Assessment of Bracket Surface Morphology and Dimensional Change. Contemporary Clinical Dentistry. 2017;8(1):71-80.	excluded/irrelevant	title
Wahab RM, Idris H, Yacob H, Ariffin SH. Comparison of self- and conventional-ligating brackets in the alignment stage. Eur J Orthod. 2012;34(2):176-81.	excluded/ineligible comparator	full text
Sander FG, Wichelhaus A, Schiemann C. Intrusion mechanics according to Burstone with the NiTi-SE-steel uprighting spring. J Orofac Orthop. 1996;57(4):210-23.	excluded/irrelevant	title
bdrc RBR. Evaluation of microbial contamination of aesthetic orthodontic ligatures by using a molecular biology technique. https://trialsearchwhoint/Trial2aspx?TrialID=RBR-4bdrc9. 2019.	excluded/protocol	abstract
Chimenti C, Franchi L, Di Giuseppe MG, Lucci M. Friction of orthodontic elastomeric ligatures with different dimensions. Angle Orthod. 2005;75(3):421-5.	excluded/irrelevant	abstract
Ortan YO, Arslan TY, Aydemir B. A comparative in vitro study of frictional resistance between lingual brackets and stainless steel archwires. European Journal of Orthodontics. 2012;34(1):119-25.	excluded/irrelevant	title
Alcan T, Ceylanoglu C. Upper midline correction in conjunction with rapid maxillary expansion. Am J Orthod Dentofacial Orthop. 2006;130(5):671-5.	excluded/irrelevant	title
Nct. Influence of Four Orthodontic Bracket Prescriptions in Arch Width and Torque. https://clinicaltrialsgov/show/NCT04043013. 2019.	excluded/ irrelevant	title
Yu Y, Sun J, Lai W, Wu T, Koshy S, Shi Z. Interventions for managing relapse of the lower front teeth after orthodontic treatment. Cochrane Database of Systematic Reviews. 2013(9).	excluded/irrelevant	title
Prévost J, Nivoit M, Granjon Y. Management of dental trauma: development of a 2D data acquisition system to evaluate passivity of dental splints. Med Biol Eng Comput. 1997;35(4):409-14.	excluded/irrelevant	title
Stocker T, Li H, Bamidis EP, Baumert U, Hoffmann L, Wichelhaus A, et al. Influence of normal forces on the frictional behavior in tribological systems made of different bracket types and wire dimensions. Dental materials journal. 2022;41(3):402-13.	excluded/irrelevant	title
Nct. Efficacy of Twin Arch Brackets in the Control of Orthodontic Tooth Movement. https://clinicaltrialsgov/show/NCT05071599. 2021.	excluded/ irrelevant	title
Montasser MA, Keilig L, El-Bialy T, Reimann S, Jäger A, Bourauel C. Effect of archwire cross-section changes on force levels during complex tooth alignment with conventional and self-ligating brackets. Am J Orthod Dentofacial Orthop. 2015;147(4 Suppl):S101-8.	excluded/irrelevant	title
Greene M, Rizkalla A, Burkhart T, Mamandras A, Tassi A. Friction and archwire engagement in contemporary self-ligating appliance systems An in vitro comparison. Journal of Orofacial Orthopedics-Fortschritte Der Kieferorthopadie. 2021.	excluded/irrelevant	title
Lo Giudice A, Nucera R, Matarese G, Portelli M, Cervino G, Lo Giudice G, et al. Analysis of resistance to sliding expressed during first order correction with conventional and self-ligating brackets: An in-vitro study. International Journal of Clinical and Experimental Medicine. 2016;9(8):15575-81.	excluded/irrelevant	title
Shirozaki MU, Ferreira JT, Küchler EC, Matsumoto MA, Aires CP, Nelson-Filho P, Romano FL. Quantification of Streptococcus mutans in different types of ligature wires and elastomeric chains. Brazilian dental journal. 2017 Jul;28:498-503.	included	
Thorstenson GA, Kusy RP. Effects of ligation type and method on the resistance to sliding of novel orthodontic brackets with second-order angulation in the dry and wet states. Angle Orthod. 2003;73(4):418-30.	excluded/irrelevant	title
Yang X, Xue C, He Y, Zhao M, Luo M, Wang P, et al. Transversal changes, space closure, and efficiency of conventional and self-ligating appliances : A quantitative systematic review. J Orofac Orthop. 2018;79(1):1-10.	excluded/irrelevant	abstract
Seo YJ, Lim BS, Park YG, Yang IH, Ahn SJ, Kim TW, et al. Effect of tooth displacement and vibration on frictional force and stick-slip phenomenon in conventional brackets: a preliminary in vitro mechanical analysis. Eur J Orthod. 2015;37(2):158-63.	excluded/ineligible outcome	full text
Rosenberg ES, Garber DA. A temporary-permanent splint. Refuat Hapeh Vehashinayim. 1979;28(2):33-7, 27-30.	excluded/irrelevant	title
Garcez AS, Suzuki SS, Ribeiro MS, Mada EY, Freitas AZ, Suzuki H. Biofilm retention by 3 methods of ligation on orthodontic brackets: a microbiologic and optical coherence tomography analysis. Am J Orthod Dentofacial Orthop. 2011;140(4):e193-8.	included	
de Almeida MR, Herrero F, Fattal A, Davoody AR, Nanda R, Uribe F. A comparative anchorage control study between conventional and self-ligating bracket systems using differential moments. Angle Orthod. 2013;83(6):937-42.	excluded/irrelevant	title
Cacciafesta V, Sfondrini MF. Correction of horizontal and vertical discrepancies with a new interactive self-ligating bracket system: the Quick system. World J Orthod. 2010;11(4):404-12.	excluded/irrelevant	abstract
Baccetti T, Franchi L. Friction produced by types of elastomeric ligatures in treatment mechanics with the preadjusted appliance. Angle Orthod. 2006;76(2):211-6.	excluded/irrelevant	title
Castro RM, Neto PS, Rebello Horta MC, Pithon MM, Oliveira DD. Comparison of static friction with self-ligating, modified slot design and conventional brackets. Journal of Applied Oral Science. 2013;21(4):314-9.	excluded/ineligible intervention	full text
Lee SM, Hwang C-J. A comparative study of frictional force in self-ligating brackets according to the bracket-archwire angulation, bracket material, and wire type. Korean Journal of Orthodontics. 2015;45(1):13-9.	excluded/irrelevant	title
Ireland AJ, Songra G, Clover M, Atack NE, Sherriff M, Sandy JR. Effect of gender and Frankfort mandibular plane angle on orthodontic space closure: a randomized controlled trial. Orthod Craniofac Res. 2016;19(2):74-82.	excluded/irrelevant	title
Nct. SmartArch vs Super Elastic NiTi Aligning Archwires. https://clinicaltrialsgov/show/NCT05510206. 2022.	excluded/ irrelevant	title
Ferrando Cascales Á, Ferrando Cascales R, Lacal Luján J, Abella Sans F. Forced orthodontic extrusion for an apparently hopeless anterior tooth by using a simplified approach. The Journal of prosthetic dentistry. 2022.	excluded/irrelevant	abstract
Maltagliati LA, Myiahira YI, Fattori L, Filho LC, Cardoso M. Transversal changes in dental arches from non-extraction treatment with self ligating brackets. Dental Press J Orthod. 2013;18(3):39-45.	excluded/irrelevant	title
Kaklamanos EG, Mavreas D, Tsalikis L, Karagiannis V, Athanasiou AE. Treatment duration and gingival inflammation in Angle's Class I malocclusion patients treated with the conventional straight-wire method and the Damon technique: a single-centre, randomised clinical trial. J Orthod. 2017;44(2):75-81.	excluded/irrelevant	title
Edwards GD, Davies EH, Jones SP. The ex vivo effect of ligation technique on the static frictional resistance of stainless steel brackets and archwires. Br J Orthod. 1995;22(2):145-53.	excluded/ineligible intervention	full text
Vág J, Gánti B, Mikecs B, Szabó E, Molnár B, Lohinai Z. Epinephrine penetrates through gingival sulcus unlike keratinized gingiva and evokes remote vasoconstriction in human. BMC Oral Health. 2020;20(1):305.	excluded/irrelevant	title
Schumacher HA, Bourauel C, Drescher D. The influence of bracket design on frictional losses in the bracket/arch wire system. J Orofac Orthop. 1999;60(5):335-47.	excluded/irrelevant	title
Lee S, Moon C-H. Orthodontic treatment in a patient with Moebius syndrome: A case report. Korean J Orthod. 2022;52(6):451-60.	excluded/irrelevant	title
Lee D, Heo G, El-Bialy T, Carey JP, Major PW, Romany DL. Initial forces experienced by the anterior and posterior teeth during dental anchored or skeletal-anchored en masse retraction in vitro. Angle Orthodontist. 2017;87(4):549-55.	excluded/ irrelevant	abstract
Badawi HM, Toogood RW, Carey JP, Heo G, Major PW. Three-dimensional orthodontic force measurements. Am J Orthod Dentofacial Orthop. 2009;136(4):518-28.	excluded/irrelevant	title
차정열, 김경석, 김동춘, 황충주. Evaluation of friction of ceramic brackets in various bracket-wire combinations. The Korean Journal of Orthodontics. 2006;36(2):125-35.	excluded/irrelevant	title
Dalstra M, Eriksen H, Bergamini C, Melsen B. Actual versus theoretical torsional play in conventional and self-ligating bracket systems. J Orthod. 2015;42(2):103-13.	excluded/irrelevant	title
Buljan ZI, Ribaric SP, Abram M, Ivankovic A, Spalj S. In vitro oxidative stress induced by conventional and self-ligating brackets. Angle Orthodontist. 2012;82(2):340-5.	excluded/ineligible outcome	abstract
Fleming PS, Lee RT, McDonald T, Pandis N, Johal A. The timing of significant arch dimensional changes with fixed orthodontic appliances: data from a multicenter randomised controlled trial. J Dent. 2014;42(1):1-6.	excluded/irrelevant	title
Condò R, Casaglia A, Armellin E, Condò SG, Cerroni L. Traditional elastic ligatures versus slide ligation system. A morphological evaluation. ORAL and Implantology. 2013;6(1):15-24.	excluded/ineligible intervention	full text
Schneevoigt R, Bourauel C, Harzer W, Eckardt L. Biomechanical analysis of arch-guided molar distalization when employing superelastic NiTi coil springs. J Orofac Orthop. 1999;60(2):124-35.	excluded/non-english language	title
Zhu K, Wang CL, Wang J, Zhao YH. [Comparison study of friction of FAS self-ligating bracket and traditional self-ligating bracket]. Hua Xi Kou Qiang Yi Xue Za Zhi. 2007;25(4):371-4.	excluded/ineligible comparator	full text
Cattaneo PM, Tepedino M, Hansen EB, Gram AR, Cornelis MA. Operating time for wire ligation with self-ligating and conventional brackets: A standardized in vitro study. Clinical and Experimental Dental Research. 2022.	excluded/irrelevant	title
Turpin DL. In-vivo studies offer best measure of self-ligation. Am J Orthod Dentofacial Orthop. 2009;136(2):141-2.	excluded/irrelevant	title
Hu B, Song J, Wu P, He P, Deng F, Gao X. [A comparative study on three-dimensional movement of anterior teeth between applying MDD appliances and applying three common fixed appliances in the initial alignment stage]. Sheng Wu Yi Xue Gong Cheng Xue Za Zhi. 2013;30(5):1039-43.	excluded/irrelevant	title
Kortam S, Deguchi T, Geuy P, Johnston WM, Fields HW, Palomo JM, et al. Associations of pretreatment parameters with changes during orthodontic treatment. Orthod Craniofac Res. 2019;22 Suppl 1:120-6.	excluded/irrelevant	title
Buzzoni R, Elias CN, Fernandes DJ, Miguel JAM. Assessment of surface friction of self-ligating brackets under conditions of angulated traction. Dental Press Journal of Orthodontics. 2012;17(4):51-6.	excluded/irrelevant	title
Iwasaki LR, Beatty MW, Randall CJ, Nickel JC. Clinical ligation forces and intraoral friction during sliding on a stainless steel archwire. Am J Orthod Dentofacial Orthop. 2003;123(4):408-15.	excluded/irrelevant	title
Bazakidou E, Nanda RS, Duncanson MG, Jr., Sinha P. Evaluation of frictional resistance in esthetic brackets. Am J Orthod Dentofacial Orthop. 1997;112(2):138-44.	excluded/irrelevant	title
Yu Z, Jiaqiang L, Weiting C, Wang Y, Zhen M, Ni Z. Stability of treatment with self-ligating brackets and conventional brackets in adolescents: a long-term follow-up retrospective study. Head Face Med. 2014;10:41.	excluded/ineligible comparator	full text

Danisman H, Celebi F, Danisman S, Bicakci AA. Effects of diamond-like carbon coating on frictional and mechanical properties of orthodontic brackets: An in vitro study. Apos Trends in Orthodontics. 2022;12(1):13-9.	excluded/irrelevant	title
Lima DVd, Freitas KMSd, Ursi W, Matias M. Bráquetes autoligáveis. Ortho Sci, Orthod sci pract. 2016;9(35):58-64.	excluded/ irrelevant	title
Pan YC, Zhang D, Fu MK. [Changes of Streptococcus mutans concentration of plaque during fixed appliance treatment]. Zhonghua Kou Qiang Yi Xue Za Zhi. 2007;42(1):41-2.	excluded/non-english language	full text
m93s RBR. Evaluation of elasticity, color change and ultrastructure of aesthetic orthodontic ligatures. https://trialsearchwhoint/Trial2aspx?TrialID=RBR-2m93s5. 2019.	excluded/irrelevant	title
Martin C, Barbieri G, Solano P, Vernal R, Herrera D, Sanz M. Bone biochemical markers in gingival crevicular fluid during orthodontic movements. Journal of dental research. 2008;87(Spec Iss C).	excluded/irrelevant	title
Cioffi I, Piccolo A, Tagliaferri R, Paduano S, Galeotti A, Martina R. Pain perception following first orthodontic archwire placement--thermoelastic vs superelastic alloys: a randomized controlled trial. Quintessence Int. 2012;43(1):61-9.	excluded/irrelevant	title
Franchi L, Baccetti T. Forces released during alignment with a preadjusted appliance with different types of elastomeric ligatures. American Journal of Orthodontics and Dentofacial Orthopedics. 2006;129(5):687-90.	excluded/ineligible outcome	full text
Magno AF, Enoki C, Ito IY, Matsumoto MA, Faria G, Nelson-Filho P. In-vivo evaluation of the contamination of Super Slick elastomeric rings by Streptococcus mutans in orthodontic patients. Am J Orthod Dentofacial Orthop. 2008;133(4 Suppl):S104-9.	excluded/irrelevant	title
Yin Mj, Hou L, Zhang Lb, Xu Sq. A comparative study of the static friction force between LF bracket and conventional bracket. Journal of Clinical Rehabilitative Tissue Engineering Research. 2011;15(42):7859-62.	excluded/irrelevant	title
Harradine N. Self-ligating brackets increase treatment efficiency. American Journal of Orthodontics and Dentofacial Orthopedics. 2013;143(1):10-8.	excluded/irrelevant	title
Elekdag-Turk S, Cakmak F, Isci D, Turk T. 12-month self-ligating bracket failure rate with a self-etching primer. Angle Orthod. 2008;78(6):1095-100.	excluded/irrelevant	title
Felicita AS. Orthodontic extrusion of Ellis Class VIII fracture of maxillary lateral incisor – The sling shot method. Saudi Dental Journal. 2018;30(3):265-9.	excluded/irrelevant	title
Fleming PS, Johal A. Self-ligating brackets in orthodontics. A systematic review. Angle Orthod. 2010;80(3):575-84.	excluded/irrelevant	title
Qamruddin I, Khan AG, Asif FM, Karim M, Nowrin SA, Shahid F, et al. Pain Perception and Rate of Canine Retraction Through Self-Ligating Brackets and Conventional Elastomeric Ligation System: A Split Mouth Study. Pesqui bras odontopediatria clín integr. 2020;20:e5147-e.	excluded/irrelevant	title
Dehbi H, Bahije L, Zaoui F, Benyahia H. [Self-ligating brackets and friction in vivo : micro-morphological and chemical assessment of active slot surface]. L' Orthodontie francaise. 2020;91(3):249-62.	excluded/irrelevant	title
Iijima M, Zinelis S, Papageorgiou SN, Brantley W, Eliades T. Orthodontic brackets. Orthodontic Applications of Biomaterials: A Clinical Guide. Woodhead Publishing Series in Biomaterials2017. p. 75-96.	excluded/irrelevant	title
Pejda S, Varga ML, Milosevic SA, Mestrovic S, Slaj M, Repic D, et al. Clinical and microbiological parameters in patients with self-ligating and conventional brackets during early phase of orthodontic treatment. Angle Orthod. 2013;83(1):133-9.	excluded/ineligible comparator	full text
Tctr. Treatment effects of mandibular anterior position training versus a fixed Class II corrector in growing patients with skeletal Class II malocclusion. https://trialsearchwhoint/Trial2aspx?TrialID=TCTR20180220003. 2018.	excluded/irrelevant	title
Baccetti T, Franchi L, Camporesi M, Defraia E. Orthodontic forces released by low-friction versus conventional systems during alignment of apically or buccally malposed teeth. Eur J Orthod. 2011;33(1):50-4.	excluded/irrelevant	title
Kusy RP, Whitley JQ. Coefficients of Friction for Arch Wires in Stainless-Steel and Polycrystalline Alumina Bracket Slots. American Journal of Orthodontics and Dentofacial Orthopedics. 1990;98(4):300-12.	excluded/irrelevant	title
Lim S-h, 정혜진, 김광원. Changes in frictional resistance between stainless steel various orthodontic wires according to. The Korean Journal of Orthodontics. 2007;37(2):137-49.	excluded/irrelevant	title
Chen SS, Greenlee GM, Kim JE, Smith CL, Huang GJ. Systematic review of self-ligating brackets. Am J Orthod Dentofacial Orthop. 2010;137(6):726.e1-.e18; discussion -7.	excluded/irrelevant	title
Isrctn. Self-ligating brackets and elastomeric rings - a comparison of orthodontic ligation techniques on patient oral hygiene and microbial colonisation. https://trialsearchwhoint/Trial2aspx?TrialID=ISRCTN56613406. 2006.	excluded/ineligible comparator	full text
Chen H, Han B, Xu T. Effect of different combinations of bracket, archwire and ligature on resistance to sliding and axial rotational control during the first stage of orthodontic treatment: An in vitro study. Korean Journal of Orthodontics. 2019;49(1):21-31.	excluded/irrelevant	title
Ho KS, West VC. Friction ... Friction resistance between edgewise brackets and archwires. Australian orthodontic journal. 1991;12(2):95-9.	excluded/irrelevant	title
Leite VV, Lopes MB, Gonini Júnior A, Almeida MR, Moura SK, Almeida RR. Comparison of frictional resistance between self-ligating and conventional brackets tied with elastomeric and metal ligature in orthodontic archwires. Dental Press J Orthod. 2014;19(3):114-9.	excluded/irrelevant	title
Aknin JJ. [The Top Wire Appliance bracket: tooth movement and friction]. Orthod Fr. 2002;73(4):415-27.	excluded/irrelevant	title
Fleming PS, Johal A, Pandis N. The effectiveness of laceback ligatures during initial orthodontic alignment: a systematic review and meta-analysis. Eur J Orthod. 2013;35(4):539-46.	excluded/irrelevant	title
Shin K. Self-ligating Brackets May Not Have Clinical Advantages Over Conventional Brackets for the Periodontal Health of Adolescent Orthodontic Patients. J Evid Based Dent Pract. 2017;17(2):102-4.	excluded/irrelevant	title
Alobeid A, El-Bialy T, Khawatmi S, Dirk C, Jäger A, Bourauel C. Comparison of the force levels among labial and lingual self-ligating and conventional brackets in simulated misaligned teeth. Eur J Orthod. 2017;39(4):419-25.	excluded/irrelevant	title
Jung M-H. Effects of self-ligating brackets and other factors influencing orthodontic treatment outcomes: A prospective cohort study. Korean Journal of Orthodontics. 2021;51(6):397-406.	excluded/irrelevant	title
Mezomo M, de Lima ES, de Menezes LM, Weissheimer A, Allgayer S. Maxillary canine retraction with self-ligating and conventional brackets. Angle Orthod. 2011;81(2):292-7.	excluded/irrelevant	title
Sfondrini MF, Xheka E, Scribante A, Gandini P, Sfondrini G. Reconditioning of self-ligating brackets A shear bond strength study. Angle Orthodontist. 2012;82(1):158-64.	excluded/irrelevant	title
Kaur T, Tripathi T, Rai P, Kanase A. SEM evaluation of enamel surface changes and enamel microhardness around orthodontic brackets after application of CO2 laser, Er,Cr:YSGG laser and fluoride varnish: An in vivo study. Journal of Clinical and Diagnostic Research. 2017;11(9):ZC59-ZC63.	excluded/irrelevant	title
Krishnan M, Kalathil S, Abraham KM. Comparative evaluation of frictional forces in active and passive self-ligating brackets with various archwire alloys. Am J Orthod Dentofacial Orthop. 2009;136(5):675-82.	excluded/irrelevant	title
Harikrishnan P, Magesh V. Effect of patterns of stainless steel ligation on orthodontic bracket with contact boundary conditions using finite element analysis C3 - IOP Conference Series: Materials Science and Engineering. 2018;402(1).	excluded/irrelevant	title
Sharma R, Sharma K, Sawhney R. Evidence of variable bacterial colonization on coloured elastomeric ligatures during orthodontic treatment: An intermodular comparative study. Journal of Clinical and Experimental Dentistry. 2018;10(3):e271-e8.	excluded/irrelevant	title
Rose MM. The Mollin Technique. Fortschritte der Kieferorthopaedie. 1985;46(6):465-70.	excluded/ irrelevant	title
Pandis N, Fleming PS, Spineli LM, Salanti G. Initial orthodontic alignment effectiveness with self-ligating and conventional appliances: a network meta-analysis in practice. Am J Orthod Dentofacial Orthop. 2014;145(4 Suppl):S152-63.	excluded/irrelevant	title
Jakob SR, Matheus D, Jimenez-Pellegrin MC, Turssi CP, Amaral FL. Comparative study of friction between metallic and conventional interactive self-ligating brackets in different alignment conditions. Dental Press J Orthod. 2014;19(3):82-9.	excluded/irrelevant	title
Liu Y, Hou R, Jin H, Zhang X, Wu Z, Li Z, et al. Relative effectiveness of facemask therapy with alternate maxillary expansion and constriction in the early treatment of Class III malocclusion. American journal of orthodontics and dentofacial orthopedics : official publication of the American Association of Orthodontists, its constituent societies, and the American Board of Orthodontics. 2021;159(3):321-32.	excluded/irrelevant	title
Frank CA, Nikolai RJ. A comparative study of frictional resistances between orthodontic bracket and arch wire. Am J Orthod. 1980;78(6):593-609.	excluded/irrelevant	title
Jiang J, Huang Z, Ma X, Zhang Y, He T, Liu Y. Establishment and Experiment of Utility Archwire Dynamic Orthodontic Moment Prediction Model. Ieee Transactions on Biomedical Engineering. 2020;67(7):1958-68.	excluded/irrelevant	title
Anand M, Turpin DL, Jumani KS, Spiekerman CF, Huang GJ. Retrospective investigation of the effects and efficiency of self-ligating and conventional brackets. Am J Orthod Dentofacial Orthop. 2015;148(1):67-75.	excluded/ineligible comparator	abstract
Holtmann S, Konermann A, Keilig L, Reimann S, Jäger A, Montasser M, et al. Different bracket-archwire combinations for simulated correction of two-dimensional tooth malalignment: Leveling outcomes and initial force systems. J Orofac Orthop. 2014;75(6):459-70.	excluded/irrelevant	title
Karnam SK, Reddy AN, Manjith CM. Comparison of metal ion release from different bracket archwire combinations: an in vitro study. The journal of contemporary dental practice. 2012;13(3):376-81.	excluded/irrelevant	title
Cervera Sabater A, Bravo De Pedro J, Azcarate Leturia S, Uriarte Ibarrola LG, inventors; Euroortodoncia SL, assignee. Plastic bracket with mechanical retention and production method thereof patent US 07927097. 2011 Apr 19 2011.	excluded/irrelevant	title
Zhou Y, Zheng M, Lin J, Wang Y, Ni ZY. Self-ligating brackets and their impact on oral health-related quality of life in Chinese adolescence patients: a longitudinal prospective study. ScientificWorldJournal. 2014;2014:352031.	excluded/ineligible comparator	full text
Geramy A. Optimization of unilateral overjet management: three-dimensional analysis by the finite element method. Angle Orthod. 2002;72(6):585-92.	excluded/irrelevant	title
Muguruma T, Iijima M, Brantley WA, Ahluwalia KS, Kohda N, Mizoguchi I. Effects of third-order torque on frictional force of self-ligating brackets. Angle Orthod. 2014;84(6):1054-61.	excluded/irrelevant	title
Nct. Labial Alveolar Bone Thickness and Apical Root Resorption Changes Associated With Self-ligating Versus Conventional Brackets. https://clinicaltrialsgov/show/NCT02816489. 2016.	excluded/ irrelevant	title
Nct. Comparison of Oral Hygiene & Root Resorption During Orthodontic Treatment. https://clinicaltrialsgov/show/NCT02745626. 2016.	excluded/ irrelevant	title
Rodrigues CF, Sales LdAR, Vitral RWF, Fraga MR, Quintão CCA. Efeito da amarração em Ortodontia, com ligaduras elastoméricas e de aço inoxidável, na saúde periodontal. Dental press j orthod (Impr). 2011;16(1):48-56.	excluded/irrelevant	abstract
Lai TT, Chiou JY, Lai TC, Chen T, Wang HY, Li CH, et al. Perceived pain for orthodontic patients with conventional brackets or self-ligating brackets over 1 month period: A single-center, randomized controlled clinical trial. J Formos Med Assoc. 2020;119(1 Pt 2):282-9.	excluded/irrelevant	title
Dehbi H, Azaroual MF, Zaoui F, Halimi A, Benyahia H. Therapeutic efficacy of self-ligating brackets: A systematic review. Int Orthod. 2017;15(3):297-311.	excluded/irrelevant	title
Nainan O, Mitra R, Chopra SS. Re: Maxillary canine retraction with self-ligating and conventional brackets. A randomized clinical trial. By: Maurı´cio Mezomo; Eduardo S. de Lima; Luciane Macedo de Menezes; Andre' Weissheimer; Susiane Allgayer. Angle Orthod. 2011;81:292–297. Angle Orthod. 2011;81(5):926-7; author reply 7.	excluded/irrelevant	title
Archambault A, Lacoursiere R, Badawi H, Major PW, Carey J, Flores-Mir C. Torque expression in stainless steel orthodontic brackets. A systematic review. Angle Orthod. 2010;80(1):201-10.	excluded/irrelevant	abstract
Rengifo RM, Peña-Reyes D, de Freitas MR, de Freitas KMS, Aliaga-Del Castillo A, Janson G. Dental inclination with self-ligating and conventional fixed appliances, with and without rapid maxillary expansion. Orthod Craniofac Res. 2019;22(2):93-8.	excluded/irrelevant	title
Alobeid A, El-Bialy T, Reimann S, Keilig L, Cornelius D, Jäger A, et al. Comparison of the efficacy of tooth alignment among lingual and labial brackets: an in vitro study. Eur J Orthod. 2018;40(6):660-5.	excluded/irrelevant	title
Nct. A Comparative CT Evaluation of the Amount of Root Resorption in Self-ligating Versus Conventional Brackets. https://clinicaltrialsgov/show/NCT03613285. 2018.	excluded/irrelevant	title
Aboujaoude R, Kmeid R, Gebrael C, Amm E. Comparison of the accuracy of bracket positioning between direct and digital indirect bonding techniques in the maxillary arch: a three-dimensional study. Prog Orthod. 2022;23(1):31.	excluded/duplicate	duplicate
Kuroda S, Watanabe H, Nakajima A, Shimizu N, Tanaka E. Evaluation of torque moment in a novel elastic bendable orthodontic wire. Dent Mater J. 2014;33(3):363-7.	excluded/irrelevant	title
Mallory DC, English JD, Powers JM, Brantley WA, Bussa HI. Force-deflection comparison of superelastic nickel-titanium archwires. American Journal of Orthodontics and Dentofacial Orthopedics. 2004;126(1):110-2.	excluded/irrelevant	title
Wiltshire WA. Determination of fluoride from fluoride-releasing elastomeric ligature ties. American Journal of Orthodontics and Dentofacial Orthopedics. 1996;110(4):383-7.	excluded/irrelevant	title

Gameiro GH, Nouer DF, Cenci MS, Cury JA. Enamel demineralization with two forms of archwire ligation investigated using an in situ caries model--a pilot study. Eur J Orthod. 2009;31(5):542-6.	excluded/irrelevant	title
Yang Y, Wang L, Chen W, Zhang Z. Establishment of an animal model of periodontitis using the old rat of kidney deficiency physique. Medical Journal of Wuhan University. 2011;32(1):65-8.	excluded/animal study	abstract
Matarese G, Nucera R, Militi A, Mazza M, Portelli M, Festa F, et al. Evaluation of frictional forces during dental alignment: an experimental model with 3 nonleveled brackets. Am J Orthod Dentofacial Orthop. 2008;133(5):708-15.	excluded/irrelevant	title
Burrow SJ, Proffit WR, Keim RG. Drs. S.J. "Jack" Burrow and William R. Proffit on the efficacy of self-ligating brackets. J Clin Orthod. 2013;47(7):413-8.	excluded/irrelevant	title
Klocke A, Kahl-Nieke B. Influence of force location in orthodontic shear bond strength testing. Dental Materials. 2005;21(5):391-6.	excluded/irrelevant	title
Tecco S, Di Lorio D, Corclasco G, Verrocchi I, Festa F. An in vitro investigation of the influence of self-ligating brackets, low friction ligatures, and archwire on frictional resistance. European Journal of Orthodontics. 2007;29(4):390-7.	excluded/irrelevant	title
Sakuda M, Tanne K, Araki K, Kinami H, Ishibe H, Kokubu K. [Mechanical properties of nickel-titanium alloy wire developed by the diffusion method]. [Osaka Daigaku shigaku zasshi] The journal of Osaka University Dental Society. 1989;34(2):392-9.	excluded/irrelevant	title
Haskova JE, Palmer G, Jones SP. An ex vivo laboratory study to determine the static frictional resistance of a variable ligation orthodontic bracket system. J Orthod. 2008;35(2):112-8; discussion 0.	excluded/irrelevant	title
Pellegrini P, Sauerwein R, Finlayson T, McLeod J, Covell DA, Jr., Maier T, et al. Plaque retention by self-ligating vs elastomeric orthodontic brackets: quantitative comparison of oral bacteria and detection with adenosine triphosphate-driven bioluminescence. Am J Orthod Dentofacial Orthop. 2009;135(4):426.e1-9; discussion -7.	excluded/ineligible comparator	full text
Jerome J, Brunson T, Takeoka G, Foster C, Moon HB, Grageda E, et al. Celebrex offers a small protection from root resorption associated with orthodontic movement. Journal of the California Dental Association. 2005;33(12):951-9.	excluded/irrelevant	title
Buzzoni R, Elias CN, Fernandes DJ, Miguel JAM. Influência da secção transversa de fios ortodônticos na fricção superficial de braquetes autoligados friction of self-ligating brackets. Dental Press Journal of Orthodontics. 2011;16(4):35.e1-.e7.	excluded/non-english language	title
Shi J, Liu Y, Hou J, Yan Z, Peng H, Chang X. [Comparison of periodontal indices and Porphyromonas gingivalis between conventional and self-ligating brackets]. Hua Xi Kou Qiang Yi Xue Za Zhi. 2013;31(3):228-31.	excluded/ineligible comparator	full text
Alabdullah MM, Burhan AS, Nabawia A, Nawaya F, Saltaji H. Comparative assessment of dental and basal arch dimensions of passive and active self-ligating versus conventional appliances A randomized clinical trial. Journal of Orofacial Orthopedics-Fortschritte Der Kieferorthopadie. 2022.	excluded/irrelevant	title
Higa RH, Henriques JFC, Janson G, Matias M, de Freitas KMS, Henriques FP, et al. Force level of small diameter nickel-titanium orthodontic wires ligated with different methods. Prog Orthod. 2017;18(1):21-.	excluded/irrelevant	title
Hamilton R, Goonewardene MS, Murray K. Comparison of active self-ligating brackets and conventional pre-adjusted brackets. Aust Orthod J. 2008;24(2):102-9.	excluded/irrelevant	abstract
Oliveira SCd, Furquim RDA, Ramos AL. Impact of brackets on smile esthetics: laypersons and orthodontists perception. Dental Press Journal of Orthodontics. 2012;17(5):64-70.	excluded/irrelevant	title
Kusy RP, O'Grady P W. Evaluation of titanium brackets for orthodontic treatment: Part II--The active configuration. Am J Orthod Dentofacial Orthop. 2000;118(6):675-84.	excluded/irrelevant	title
Arash V, Teimoorian M, Farajzadeh Jalali Y, Sheikhzadeh S. Clinical comparison between Multi-Stranded Wires and Single strand Ribbon wires used for lingual fixed retainers. Prog Orthod. 2020;21(1):22.	excluded/irrelevant	abstract
Northrup RG, Berzins DW, Bradley TG, Schuckit W. Shear bond strength comparison between two orthodontic adhesives and self-ligating and conventional brackets. Angle Orthod. 2007;77(4):701-6.	excluded/irrelevant	title
Pramustika A, Soedarsono N, Widayati R. Comparison of Tumor Necrosis Factor-α Concentrations in Gingival Crevicular Fluid between Self-Ligating and Preadjusted Edgewise Appliances in the Early Leveling Stage of Orthodontic Treatment. Contemp Clin Dent. 2018;9(1):92-6.	excluded/ineligible comparator	full text
Voudouris J, inventor; Spartan Orthodontics Inc, assignee. Orthodontic device for overbite correction patent US 11317993. 2022 May 3 2022.	excluded/irrelevant	title
Schumacher HA, Bourauel C, Drescher D. Der Einfluß der Ligatur auf die Friktion zwischen Bracket und Bogen. Fortschritte der Kieferorthopädie. 1990;51(2):106-16.	excluded/irrelevant	abstract
Owen B, Bolen B, Wubie BA, Heo G, Carey JP, Major PW, et al. Three-dimensional in vitro measurement of initial forces and moments acting on maxillary canine teeth using various Class II elastic configurations with a straight archwire fixed lingual appliance. Orthodontic Waves. 2019;78(2):56-62.	excluded/irrelevant	title
Thiry P, Barthelemi S. Towards slide enhancement with the titanium-molybdenum wire? International orthodontics. 2010;8(4):319-41.	excluded/irrelevant	title
Jones SP, Ben Bihi S. Static frictional resistance with the slide low-friction elastomeric ligature system. Aust Orthod J. 2009;25(2):136-41.	excluded/irrelevant	title
Alpern MC. Gaining Control with Self-Ligation. Seminars in Orthodontics. 2008;14(1):73-86.	excluded/irrelevant	title
Johal A, Bourauel C, Moghanchi S, Dickerson T, Gaudin P, Elliott EL. The Tip and Torque adjustable bracket as a new concept in design: An in vitro study. Angle Orthodontist. 2022;92(3):380-7.	excluded/irrelevant	title
Blomlof L, Lindskog S, Appelgren R, Jonsson B, Weintraub A, Hammarstrom L. New Attachment in Monkeys with Experimental Periodontitis with and without Removal of the Cementum. Journal of Clinical Periodontology. 1987;14(3):136-43.	excluded/animal study	abstract
Tecco S, Tete S, Festa F. Friction between Archwires of Different Sizes, Cross-Section and Alloy and Brackets Ligated with Low-Friction or Conventional Ligatures. Angle Orthodontist. 2009;79(1):111-6.	excluded/irrelevant	title
Franchi L, Baccetti T, Camporesi M, Barbato E. Forces released during sliding mechanics with passive self-ligating brackets or nonconventional elastomeric ligatures. American Journal of Orthodontics and Dentofacial Orthopedics. 2008;133(1):87-90.	excluded/irrelevant	title
Guo J, Wang W, Yao L, Yan F. Local inflammation exacerbates cyclosporine a-induced gingival overgrowth in rats. Inflammation. 2008;31(6):399-407.	excluded/irrelevant	title
Romanyk DL, Au K, Isfeld D, Heo G, Major MP, Major PW. The effect of buccal-lingual slot dimension size on third-order torque response. European Journal of Orthodontics. 2017;39(2):209-14.	excluded/irrelevant	title
Katona TR, Chen J. Engineering and Experimental Analyses of the Tensile Loads Applied during Strength Testing of Direct-Bonded Orthodontic Brackets. American Journal of Orthodontics and Dentofacial Orthopedics. 1994;106(2):167-74.	excluded/irrelevant	title
Qin YJ, Zhang GD, Zhang Y, Ping YF, Zhao CY. Natural reversal of tooth discoloration and pulpal response to testing following removal of a miniscrew implant for orthodontic anchorage: a case report. Int Endod J. 2016;49(4):402-9.	excluded/ irrelevant	title
Nilforoushan D, Shirazi M, Dehpour A-R. The role of opioid systems on orthodontic tooth movement in cholestatic rats. Angle Orthod. 2002;72(5):476-80.	excluded/irrelevant	title
Fortini A, Lupoli M, Cacciafesta V. A new low-friction ligation system. J Clin Orthod. 2005;39(8):464-70; quiz 71.	excluded/irrelevant	title
Papageorgiou SN, Sifakakis I, Doulis I, Eliades T, Bourauel C. Torque efficiency of square and rectangular archwires into 0.018 and 0.022 in. conventional brackets. Progress in Orthodontics. 2016;17.	excluded/irrelevant	title
Lombardo L. Ectopic canine control with conventional brackets. Int Orthod. 2012;10(4):377-403.	excluded/irrelevant	title
Bi S, Guo Z, Zhang X, Shi G. Anchorage effects of ligation and direct occlusion in orthodontics: A finite element analysis. Comput Methods Programs Biomed. 2022;226:107142-.	excluded/irrelevant	title
Kusy RP. Ongoing innovations in biomechanics and materials for the new millennium. Angle Orthod. 2000;70(5):366-76.	excluded/irrelevant	title
Pandis N, Eliades T, Partowi S, Bourauel C. Moments generated during simulated rotational correction with self-ligating and conventional brackets. Angle Orthod. 2008;78(6):1030-4.	excluded/irrelevant	title
El-Bialy T, Alobeid A, Dirk C, Jäger A, Keilig L, Bourauel C. Comparison of force loss due to friction of different wire sizes and materials in conventional and new self-ligating orthodontic brackets during simulated canine retraction. J Orofac Orthop. 2019;80(2):68-78.	excluded/irrelevant	title
Marín MJ, Ambrosio N, O'Connor A, Herrera D, Sanz M, Figuero E. Validation of a multiplex qPCR assay for detection and quantification of Aggregatibacter actinomycetemcomitans, Porphyromonas gingivalis and Tannerella forsythia in subgingival plaque samples. A comparison with anaerobic culture. Arch Oral Biol. 2019;102:199-204.	excluded/irrelevant	title
Lee SA, Chang CCH, Roberts WE. Severe unilateral scissors-bite with a constricted mandibular arch: Bite turbos and extra-alveolar bone screws in the infrazygomatic crests and mandibular buccal shelf. Am J Orthod Dentofacial Orthop. 2018;154(4):554-69.	excluded/irrelevant	title
Pinto LS, Nakane Matsumoto MA, Romualdo PC, Romano FL, da Silva RAB, da Silva LAB, et al. Esthetic elastomeric ligatures: Quantification of bacterial endotoxin in vitro and in vivo. Am J Orthod Dentofacial Orthop. 2021;159(5):660-5.	excluded/irrelevant	title
David Partouche AJ, Castro F, Baptista AS, Costa LG, Fernandes JCH, Fernandes GVdO. Effects of Multibracket Orthodontic Treatment versus Clear Aligners on Periodontal Health: An Integrative Review. Dentistry Journal. 2022;10(10).	excluded/review	title
Bednar JR, Gruendeman GW, Sandrik JL. A Comparative-Study of Frictional Forces between Orthodontic Brackets and Arch Wires. American Journal of Orthodontics and Dentofacial Orthopedics. 1991;100(6):513-22.	excluded/irrelevant	title
Buck T, Pellegrini P, Sauerwein R, Leo MC, Covell DA, Jr., Maier T, et al. Elastomeric-ligated vs self-ligating appliances: a pilot study examining microbial colonization and white spot lesion formation after 1 year of orthodontic treatment. Orthodontics (Chic). 2011;12(2):108-21.	excluded/ineligible comparator	full text
Zufall SW, Kusy RP. Sliding mechanics of coated composite wires and the development of an engineering model for binding. Angle Orthod. 2000;70(1):34-47.	excluded/irrelevant	title
Park JH, Lee YK, Lim BS, Kim CW. Frictional forces between lingual brackets and archwires measured by a friction tester. Angle Orthodontist. 2004;74(6):816-24.	excluded/irrelevant	title
Nam HJ, Flores-Mir C, Major PW, Heo G, Kim J, Lagravère MO. Dental and skeletal changes associated with the Damon system philosophical approach. Int Orthod. 2019;17(4):621-33.	excluded/irrelevant	title
Faber J. Tying twin brackets. Am J Orthod Dentofacial Orthop. 2000;118(1):101-6.	excluded/irrelevant	title
Miranda AGF, Godoi APT, Menezes CC, Vedovello Filho M, Venezian GC. The influence of elastomeric ligatures pigmentation on smile aesthetics during orthodontic treatment. Dental Press J Orthod. 2021;26(2):e2119199.	excluded/irrelevant	title
Ozer M, Bayram M, Dincyurek C, Tokalak F. Clinical bond failure rates of adhesive precoated self-ligating brackets using a self-etching primer. Angle Orthod. 2014;84(1):155-60.	excluded/irrelevant	title
Sukh R, Singh GK, Tandon P, Singh GP, Singh A. A comparative study of frictional resistance during simulated canine retraction on typodont model. J Orthod Sci. 2013;2(2):61-6.	excluded/irrelevant	title
Cannavale R, Matarese G, Isola G, Grassia V, Perillo L. Early treatment of an ectopic premolar to prevent molar-premolar transposition. Am J Orthod Dentofacial Orthop. 2013;143(4):559-69.	excluded/ irrelevant	title
Miles PG, Weyant RJ, Rustveld L. A clinical trial of Damon 2 (TM) vs conventional twin brackets during initial alignment. Angle Orthodontist. 2006;76(3):480-5.	excluded/irrelevant	title
Kuijpers-Jagtman AM. [Repair and revision 8. Relapse of lower incisors: retreatment?]. Ned Tijdschr Tandheelkd. 2002;109(2):42-6.	excluded/irrelevant	title
Baek S-H, Seo Y-J. Application of orthodontic mini-implants and ligation for absolute skeletal anchorage to the intraoral labiolingual appliance: midface distraction osteogenesis cases treated with the RED System. J Craniofac Surg. 2011;22(2):609-13.	excluded/irrelevant	title
Mezeg U, Primožic J. Influence of long-term in vivo exposure, debris accumulation and archwire material on friction force among different types of brackets and archwires couples. Eur J Orthod. 2017;39(6):673-9.	excluded/irrelevant	title
Butti AC, Mangiacapra R, Saporito I, Augusti G, Salvato A, Re D. Second order root control of self-ligating brackets and traditional brackets: a "typodont" study. Minerva stomatologica. 2014;63(3):51-7.	excluded/ineligible comparator	title
Zucch TU, Nóbrega C, Benetti JJ, Gick MR, Jakob SR, Arsati F. Avaliação da força liberada por fios ortodônticos de níquel-titânio associados a bráquetes autoligante. Ortho Sci, Orthod sci pract. 2011;3(13):407-13.	excluded/non-english language	title
Sinato G, Guiducci D, Ghislanzoni LH, Albertini P. Carriere Motion (R) 3D (TM) appliance followed by Self-ligating brackets System in the correction of Class II malocclusion: dentoalveolar and skeletal effects compared to the use of intermaxillary elastics. Journal of Biological Regulators and Homeostatic Agents. 2022;36(2):115-26.	excluded/irrelevant	title
Jurela A, Sudarevic K, Budimir A, Brailo V, Loncar Brzak B, Jankovic B. Clinical and Salivary Findings in Patients with Metal and Crystalline Conventional and Self-Ligating Orthodontic Brackets. Acta Stomatol Croat. 2019;53(3):224-30.	excluded/ineligible comparator	full text
Muguruma T, Iijima M, Brantley WA, Mizoguchi I. Effects of a diamond-like carbon coating on the frictional properties of orthodontic wires. Angle Orthodontist. 2011;81(1):141-8.	excluded/irrelevant	title
Kim D-Y, Lim B-S, Baek S-H. Frictional property comparisons of conventional and self-ligating lingual brackets according to tooth displacement during initial leveling and alignment: an in vitro mechanical study. Korean Journal of Orthodontics. 2016;46(2):87-95.	excluded/irrelevant	title

Reichardt E, Decker S, Dalstra M, Nalabothu P, Steineck M, Fernandez L, et al. The Effect of Ligature Type on Lateral Tooth Movement during Orthodontic Treatment with Lingual Appliances—An In Vitro Study. Materials. 2022;15(9).	excluded/irrelevant	title
Fleming PS, Lee RT, McDonald T, Pandis N, Johal A. The timing of significant arch dimensional changes with fixed orthodontic appliances: data from a multicenter randomised controlled trial. Journal of dentistry. 2014;42(1):1-6.	excluded/irrelevant	title
Pesce RE, Uribe F, Janakiraman N, Neace WP, Peterson DR, Nanda R. Evaluation of rotational control and forces generated during first-order archwire deflections: a comparison of self-ligating and conventional brackets. Eur J Orthod. 2014;36(3):245-54.	excluded/irrelevant	title
Nct. A Randomized Clinical Trial to Investigate Whether Self Ligating Brackets or Conventional Brackets Are Better for Treating Bimaxillary Proclination. https://clinicaltrialsgov/show/NCT04001816. 2019.	excluded/irrelevant	title
Pizzatto LV, Prevedello LR, Pizzatto S, Losso EM, Pizzatto E, Shimizu IA. Avaliação do índice de biofilme dentário em pacientes sob tratamento ortodôntico corretivo. Ortho Sci, Orthod sci pract. 2018;11(43):72-6.	excluded/irrelevant	title
Heiser W, inventorSelf-ligating orthodontic bracket patent US 07967603. 2011 Jun 28 2011.	excluded/irrelevant	title
Kamarudin Y, Skeats MK, Ireland AJ, Barbour ME. Chlorhexidine hexametaphosphate as a coating for elastomeric ligatures with sustained antimicrobial properties: A laboratory study. Am J Orthod Dentofacial Orthop. 2020;158(5):e73-e82.	excluded/irrelevant	title
[No authors] The Role of Friction in Orthodontic Appliances. Biomechanical Foundation of Clinical Orthodontics2015. p. 453-74.	excluded/irrelevant	title
Tiwari A, Jain RK. Comparison of enamel demineralisation scores between passive self-ligation brackets and conventional ligation brackets in patients undergoing orthodontic treatment-a laser fluorescence study. Journal of Clinical and Diagnostic Research. 2020;14(11):ZC16-ZC9.	excluded/ineligible comparator	full text
Parkin N. Clinical pearl: clinical tips with System-R. J Orthod. 2005;32(4):244-6.	excluded/irrelevant	title
Khoroushi M, Kachuie M. Prevention and Treatment of White Spot Lesions in Orthodontic Patients. Contemporary Clinical Dentistry. 2017;8(1):11-9.	excluded/irrelevant	title
Shaughnessy T, Kantarci A, Kau CH, Skrenes D, Skrenes S, Ma D. Intraoral photobiomodulation-induced orthodontic tooth alignment: a preliminary study. BMC oral health. 2016;16:3.	excluded/irrelevant	title
Santos RATd. Avaliação da aderência de biofilme bacteriano em bráquetes metálicos, plásticos e cerâmicos. 2011. p. 47-.	excluded/non-english language	title
Abu Alhaija ES, Taha NA. A comparative study of initial changes in pulpal blood flow between conventional and self-ligating fixed orthodontic brackets during leveling and alignment stage. Clin Oral Investig. 2021;25(3):971-81.	excluded/ineligible outcome	full text
Gandini P, Orsi L, Sfondrini MF, Scribante A. Opening and closure forces of sliding mechanisms of different self-ligating brackets. Journal of Applied Oral Science. 2013;21(3):231-4.	excluded/irrelevant	title
Colby CJ, Herrod R, Miloro M. Tooth autotransplantation with a figure-of-eight wire technique. Journal of Oral and Maxillofacial Surgery. 2017;75(10):e376.	excluded/irrelevant	title
Ctri. A study to compare the amount of backward movement of teeth between newer lockable kind of dental brackets and ordinary dental brackets using laser. https://trialsearchwhoint/Trial2aspx?TrialID=CTRI/2018/04/013228. 2018.	excluded/irrelevant	title
Bantleon HP, Droschl H. Incisor Torque with the Aid of the Segmented Arch Technique. Fortschritte der Kieferorthopaedie. 1988;49(2):203-12.	excluded/irrelevant	title
Walker S. Root resorption during orthodontic treatment. Evid Based Dent. 2010;11(3):88-.	excluded/irrelevant	title
Tecco S, Marzo G, Di Bisceglie B, Crincoli V, Tetè S, Festa F. Does the design of self-ligating brackets show different behavior in terms of friction? Orthodontics (Chic). 2011;12(4):330-9.	excluded/irrelevant	title
Pandis N, Polychronopoulou A, Katsaros C, Eliades T. Comparative assessment of conventional and self-ligating appliances on the effect of mandibular intermolar distance in adolescent nonextraction patients: a single-center randomized controlled trial. Am J Orthod Dentofacial Orthop. 2011;140(3):e99-e105.	excluded/irrelevant	title
Huang Y, Keilig L, Rahimi A, Reimann S, Bourauel C. Torque capabilities of self-ligating and conventional brackets under the effect of bracket width and free wire length. Orthod Craniofac Res. 2012;15(4):255-62.	excluded/irrelevant	title
Franchi L, Baccetti T, Camporesi M. An experimental study on the forces released by ceramic preadjusted brackets with low friction vs. conventional elastomeric ligatures. Progress in orthodontics. 2007;8(2):294-9.	excluded/irrelevant	title
Becker A, Chaushu G, Chaushu S. Analysis of failure in the treatment of impacted maxillary canines. Am J Orthod Dentofacial Orthop. 2010;137(6):743-54.	excluded/duplicate	duplicate
Rafighi A, Sohrabi A, Zokaee M, Moghaddam SF, Sharghi R. Evaluation of the epithelial cells of lower lip mucosa after debonding of fixed orthodontic appliances. Minerva Stomatologica. 2020;69(4):245-50.	excluded/irrelevant	title
Olson JE, Liu Y, Nickel JC, Walker MP, Iwasaki LR. Archwire vibration and stick-slip behavior at the bracket-archwire interface. Am J Orthod Dentofacial Orthop. 2012;142(3):314-22.	excluded/irrelevant	title
Punke C, Schöntag C, Hortian B, Behrend D, Hingst V, von Schwanewede H, et al. [Tongue fixation system for therapy of sleeping disorders. A feasibility study]. HNO. 2010;58(12):1184-9.	excluded/irrelevant	title
Yanisarapan T, Thunyakitpisal P, Chantarawaratit P-o. Corrosion of metal orthodontic brackets and archwires caused by fluoride-containing products: Cytotoxicity, metal ion release and surface roughness. Orthodontic Waves. 2018;77(2):79-89.	excluded/irrelevant	title
Domínguez A, Velásquez SA. Effect of low-level laser therapy on pain following activation of orthodontic final archwires: a randomized controlled clinical trial. Photomed Laser Surg. 2013;31(1):36-40.	excluded/irrelevant	title
Mascarelo AC, Godoi AP, Furletti V, Custódio W, Valdrighi HC. Evaluation of friction in metal, ceramic and self-ligating brackets submitted to sliding mechanics. Rev odontol UNESP (Online). 2018;47(4):244-8.	excluded/irrelevant	title
Seo YJ, Lim BS, Park YG, Yang IH, Ahn SJ, Kim TW, et al. Effect of self-ligating bracket type and vibration on frictional force and stick-slip phenomenon in diverse tooth displacement conditions: an in vitro mechanical analysis. Eur J Orthod. 2015;37(5):474-80.	excluded/irrelevant	title
Hanson GH. The SPEED system: a report on the development of a new edgewise appliance. Am J Orthod. 1980;78(3):243-65.	excluded/ irrelevant	abstract
Atik E, Taner T. Stability comparison of two different dentoalveolar expansion treatment protocols. Dental Press J Orthod. 2017;22(5):75-82.	excluded/irrelevant	title
Nct. 0.018 or 0.022 Bracket Slot System More Effective in Orthodontic Treatment? https://clinicaltrialsgov/show/NCT02080338. 2014.	excluded/irrelevant	title
Phukaoluan A, Khantachawana A, Kaewtatip P, Dechkunakorn S, Anuwongnukroh N, Santiwong P, et al. Comparison of friction forces between stainless orthodontic steel brackets and TiNi wires in wet and dry conditions. International Orthodontics. 2017;15(1):13-24.	excluded/irrelevant	title
Malik DES, Fida M, Afzal E, Irfan S. Comparison of anchorage loss between conventional and self-ligating brackets during canine retraction - A systematic review and meta-analysis. Int Orthod. 2020;18(1):41-53.	excluded/irrelevant	title
Rathinasamy R, Vannala V, Mahabob N, Bhuvaneshwari S, Sam G, Ganapathy A. Evaluation of Frictional Forces Generated between Three Different Ligation Methods with Four Different Sizes of Orthodontic Archwires: An <i>In vitro</i> Study. J Pharm Bioallied Sci. 2021;13(Suppl 2):S1434-S41.	excluded/irrelevant	title
Venâncio FR, Vedovello SAS, Tubel CAM, Degan VV, Lucato AS, Lealdim LN. Effect of elastomeric ligatures on frictional forces between the archwire and orthodontic bracket. Brazilian Journal of Oral Sciences. 2013;12(1):41-5.	excluded/irrelevant	title
Scott P, DiBiase AT, Sherriff M, Cobourne MT. Alignment efficiency of Damon3 self-ligating and conventional orthodontic bracket systems: a randomized clinical trial. Am J Orthod Dentofacial Orthop. 2008;134(4):470.e1-8.	excluded/irrelevant	title
Oliver CL, Daskalogiannakis J, Tompson BD. Archwire depth is a significant parameter in the frictional resistance of active and interactive, but not passive, self-ligating brackets. Angle Orthod. 2011;81(6):1036-44.	excluded/irrelevant	title
Rodrigues CF, Sales LAR, Vitral RWF, Fraga MR, Quintão CCA. Effects of orthodontic ligation-using elastomeric and stainless steel ligatures-on periodontal health. Dental Press Journal of Orthodontics. 2011;16(1):48-56.	included	
Stefanos S, Secchi AG, Coby G, Tanna N, Mante FK. Friction between various self-ligating brackets and archwire couples during sliding mechanics. American Journal of Orthodontics and Dentofacial Orthopedics. 2010;138(4):463-7.	excluded/irrelevant	title
Khambay B, Millett D, McHugh S. Evaluation of methods of archwire ligation on frictional resistance. Eur J Orthod. 2004;26(3):327-32.	excluded/irrelevant	title
Mittal R, Rai D, Patil A, Garg A. An easy method of attachment to an impacted canine. Prog Orthod. 2013;14:11.	excluded/irrelevant	title
Read-Ward GE, Jones SP, Davies EH. A comparison of self-ligating and conventional orthodontic bracket systems. Br J Orthod. 1997;24(4):309-17.	excluded/irrelevant	title
Lima VNC, Coimbra MER, Derech CD, Ruellas ACO. Frictional forces in stainless steel and plastic brackets using four types of wire ligation. Dental Press Journal of Orthodontics. 2010;15(2):82-6.	excluded/irrelevant	title
Pollit DJ. Twist ligation: a technique to reduce archwire binding. J Clin Orthod. 1996;30(3):150-3.	excluded/irrelevant	abstract
Fleming PS, DiBiase AT, Sarri G, Lee RT. Efficiency of mandibular arch alignment with 2 preadjusted edgewise appliances. Am J Orthod Dentofacial Orthop. 2009;135(5):597-602.	excluded/irrelevant	title
Kopsahilis IE, Drescher D. Friction behavior of the wire material Gummetal (R). Journal of Orofacial Orthopedics-Fortschritte Der Kieferorthopadie. 2022;83(1):59-72.	excluded/irrelevant	title
Seghir M, Popov E, Salsedo I, Amara MA. [A new ligation method in treating maxillary fractures in children]. Rev Stomatol Chir Maxillofac. 1984;85(5):424-5.	excluded/irrelevant	title
Petersen A, Rosenstein S, Kim KB, Israel H. Force decay of elastomeric ligatures: influence on unloading force compared to self-ligation. Angle Orthod. 2009;79(5):934-8.	excluded/ineligible comparator	full text
Atik E, Akarsu-Guven B, Kocadereli I. Mandibular dental arch changes with active self-ligating brackets combined with different archwires. Niger J Clin Pract. 2018;21(5):566-72.	excluded/irrelevant	title
Sobral GC, Vedovello Filho M, Degan VV, Santamaria M, Jr. Photoelastic analysis of stress generated by wires when conventional and self-ligating brackets are used: a pilot study. Dental Press J Orthod. 2014;19(5):74-8.	excluded/irrelevant	title
Katsikogianni EN, Reimann S, Weber A, Karp J, Bourauel C. A comparative experimental investigation of torque capabilities induced by conventional and active, passive self-ligating brackets. Eur J Orthod. 2015;37(4):440-6.	excluded/irrelevant	title
Irct201302269085N. Measuring backward movement of upper posterior teeth in orthodontic patients using a new distalizer appliance. https://trialsearchwhoint/Trial2aspx?TrialID=IRCT201302269085N3. 2013.	excluded/ irrelevant	title
Baccetti T, Franchi L, Camporesi M, Defraia E, Barbato E. Forces produced by different nonconventional bracket or ligature systems during alignment of apically displaced teeth. Angle Orthod. 2009;79(3):533-9.	excluded/irrelevant	title
nn2w RBR. Color evaluation of different aesthetic elastics submitted to mouthwash with and without alcohol. https://trialsearchwhoint/Trial2aspx?TrialID=RBR-62nn2w4. 2021.	excluded/irrelevant	title
Pandis N, Strigou S, Eliades T. Maxillary incisor torque with conventional and self-ligating brackets: a prospective clinical trial. Orthod Craniofac Res. 2006;9(4):193-8.	excluded/irrelevant	title
Yadav S, Chen J, Upadhyay M, Jiang F, Roberts WE. Comparison of the force systems of 3 appliances on palatally impacted canines. Am J Orthod Dentofacial Orthop. 2011;139(2):206-13.	excluded/irrelevant	title
Lalnunpuii H, Batra P, Sharma K, Srivastava A, Raghavan S. Comparison of rate of orthodontic tooth movement in adolescent patients undergoing treatment by first bicuspid extraction and en-mass retraction, associated with low level laser therapy in passive self-ligating and conventional brackets: A randomized controlled trial. Int Orthod. 2020;18(3):412-23.	excluded/irrelevant	title
Amaral MRDd, Neto PS, Pithon MM, Oliveira DD. Evaluation In Vitro of Frictional Resistance of Self-Ligating Esthetic and Conventional Brackets Autoligado Estéticos y Convencionales. International journal of odontostomatology. 2014;8(2):261-6.	excluded/irrelevant	title
Burrow SJ. Canine retraction rate with self-ligating brackets vs conventional edgewise brackets. Angle Orthod. 2010;80(4):438-45.	excluded/irrelevant	title
Nct. Efficacy and Periodontal Parameters in Self-ligating Brackets Alone or With Corticotomy vs Conventional Brackets. https://clinicaltrialsgov/show/NCT04950829. 2021.	excluded/ irrelevant	title
Papageorgiou SN, Keilig L, Vandevska-Radunovic V, Eliades T, Bourauel C. Torque differences due to the material variation of the orthodontic appliance: a finite element study. Prog Orthod. 2017;18(1):6.	excluded/irrelevant	title
Savoldi F, Visconti L, Dalessandri D, Bonetti S, Tsoi JKH, Matinlinna JP, et al. In vitro evaluation of the influence of velocity on sliding resistance of stainless steel arch wires in a self-ligating orthodontic bracket. Orthodontics & Craniofacial Research. 2017;20(2):119-25.	excluded/irrelevant	title
Hassan SE, Hajeer MY, Alali OH, Kaddah AS. The Effect of Using Self-ligating Brackets on Maxillary Canine Retraction: A Split-mouth Design Randomized Controlled Trial. J Contemp Dent Pract. 2016;17(6):496-503.	excluded/irrelevant	title

Burrow SJ. Canine retraction rate with self-ligating brackets vs conventional edgewise brackets. Angle Orthodontist. 2010;80(4):626-33.	excluded/duplicate	duplicate
Baker KL, Nieberg LG, Weimer AD, Hanna M. Frictional Changes in Force Values Caused by Saliva Substitution. American Journal of Orthodontics and Dentofacial Orthopedics. 1987;91(4):316-20.	excluded/ineligible intervention	full text
Miles PG. Self-ligating vs conventional twin brackets during en-masse space closure with sliding mechanics. Am J Orthod Dentofacial Orthop. 2007;132(2):223-5.	excluded/irrelevant	title
Little SA, Kubba H, Hussain SSM. An evidence-based approach to the child who drools saliva. Clin Otolaryngol. 2009;34(3):236-9.	excluded/irrelevant	title
Pereira GO, Gimenez CM, Prieto L, Prieto MG, Basting RT. Influence of ligation method on friction resistance of lingual brackets with different second-order angulations: an in vitro study. Dental Press J Orthod. 2016;21(4):34-40.	excluded/irrelevant	title
Wichelhaus A. A new elastic slot system and V-wire mechanics. Angle Orthod. 2017;87(5):774-81.	excluded/irrelevant	title
Farnoush A. Techniques for the protection and coverage of the donor sites in free soft tissue grafts. J Periodontol. 1978;49(8):403-5.	excluded/irrelevant	title
Narita S, Narita K, Yamaguchi M. A Novel Technique for Shortening Orthodontic Treatment: The "JET System". Medicina (Kaunas). 2022;58(2).	excluded/irrelevant	title
Brêtas SM, Macari S, Elias AM, Ito IY, Matsumoto MA. Effect of 0.4% stannous fluoride gel on Streptococci mutans in relation to elastomeric rings and steel ligatures in orthodontic patients. Am J Orthod Dentofacial Orthop. 2005;127(4):428-33.	excluded/ineligible comparator	full text
Kawabata E, Dantas VL, Kato CB, Normando D. Color changes of esthetic orthodontic ligatures evaluated by orthodontists and patients: a clinical study. Dental press journal of orthodontics. 2016;21(5):53-7.	excluded/irrelevant	title
Dupray DJ, Trinh N, Smith JA, inventors; RMO Inc, assignee. Self ligating orthodontic bracket having a rotatable member patent US 09987105. 2018 Jun 5 2018.	excluded/ irrelevant	title
Leite V, Conti AC, Navarro R, Almeida M, Oltramari-Navarro P, Almeida R. Comparison of root resorption between self-ligating and conventional preadjusted brackets using cone beam computed tomography. Angle Orthod. 2012;82(6):1078-82.	excluded/irrelevant	title
Liu XQ, Sun XL, Yang Q, Fan CH, Chen XJ. [Comparative study on the apical root resorption between self-ligating and conventional brackets in extraction patients]. Shanghai Kou Qiang Yi Xue. 2012;21(4):460-5.	excluded/irrelevant	title
Ubios AM, Costa OR, Cabrini RL. Early steps in bone resorption in experimental periodontitis: a histomorphometric study. Acta Odontol Latinoam. 1993;7(1):45-50.	excluded/irrelevant	title
Kim SJ, Kwon YH, Hwang CJ. Biomechanical characteristics of self-ligating brackets in a vertically displaced canine model: a finite element analysis. Orthod Craniofac Res. 2016;19(2):102-13.	excluded/irrelevant	title
Little RA, Spary DJ. The effect of conventional versus figure-of-eight module ligation on mandibular incisor alignment: a randomised controlled trial. J Orthod. 2017;44(4):231-40.	excluded/irrelevant	title
Johannessen L, Keilig L, Reimann S, Jäger A, Bourauel C. First order couples induced by nickel-titanium archwires featuring an electrochemically refined surface during simulated rotation of teeth. J Orofac Orthop. 2013;74(2):153-64.	excluded/irrelevant	title
Atik E, Ciğer S. An assessment of conventional and self-ligating brackets in Class I maxillary constriction patients. Angle Orthod. 2014;84(4):615-22.	excluded/irrelevant	title
Miller R, Sakamoto E, Zell A, Arthur A, Stratigos GT. Cleidocranial dysostosis: a multidisciplinary approach to treatment. J Am Dent Assoc. 1978;96(2):296-300.	excluded/ irrelevant	abstract
Tecco S, D'Attilio M, Tetè S, Festa F. Prevalence and type of pain during conventional and self-ligating orthodontic treatment. Eur J Orthod. 2009;31(4):380-4.	excluded/irrelevant	title
Harradine N. Northcroft Memorial Lecture self-ligation: past, present and future. J Orthod. 2009;36(4):260-71.	excluded/irrelevant	title
Naziris K, Piro NE, Jaeger R, Schmidt F, Elkholy F, Lapatki BG. Experimental friction and deflection forces of orthodontic leveling archwires in three-bracket model experiments. Journal of Orofacial Orthopedics-Fortschritte Der Kieferorthopadie. 2019;80(5):223-35.	excluded/irrelevant	title
Cordasco G, Farronato G, Festa F, Nucera R, Parazzoli E, Grossi GB. In vitro evaluation of the frictional forces between brackets and archwire with three passive self-ligating brackets. Eur J Orthod. 2009;31(6):643-6.	excluded/irrelevant	title
Kraus CD, Campbell PM, Spears R, Taylor RW, Buschange PH. Bony adaptation after expansion with light-to-moderate continuous forces. American Journal of Orthodontics and Dentofacial Orthopedics. 2014;145(5):655-66.	excluded/irrelevant	title
Fathimani M, Melenka GW, Romanyk DL, Toogood RW, Heo G, Carey JP, et al. Development of a standardized testing system for orthodontic sliding mechanics. Prog Orthod. 2015;16:14.	excluded/irrelevant	title
Edwards IR, Spary DJ, Rock WP. The effect upon friction of the degradation of orthodontic elastomeric modules. European Journal of Orthodontics. 2012;34(5):618-24.	excluded/irrelevant	title
Ctri. Comparison between arch width and inclination of the incisors with Self-ligating & Conventional brackets. https://trialsearchwhoint/Trial2aspx?TrialID=CTRI/2022/09/045206. 2022.	excluded/irrelevant	title
Irct20181203041837N. Effect of chlorhexidine-releasing elastomerics on Streptococcus mutans Levels in orthodontic patients Saliva. https://trialsearchwhoint/Trial2aspx?TrialID=IRCT20181203041837N1. 2020.	excluded/protocol	title
Sepolia S, Kushwah AP, Natt AS, Vashisht L, Sahoo SK, Subudhi SK. Retrospective Analysis of Different Bracket Systems used in the Treatment of Patients with Anterior Crowding: A Longitudinal Comparative Study. J Contemp Dent Pract. 2016;17(8):687-91.	excluded/irrelevant	title
Fleming PS. Self-Ligation. Evidence-Based Orthodontics2011. p. 135-47.	excluded/irrelevant	title
Scott P, Sherriff M, Dibiase AT, Cobourne MT. Perception of discomfort during initial orthodontic tooth alignment using a self-ligating or conventional bracket system: a randomized clinical trial. Eur J Orthod. 2008;30(3):227-32.	excluded/irrelevant	title
Handem RH, Janson G, Matias M, de Freitas KM, de Lima DV, Garib DG, et al. External root resorption with the self-ligating Damon system-a retrospective study. Prog Orthod. 2016;17(1):20.	excluded/irrelevant	title
Sirisaowaluk N, Kravchuk O, Ho CT. The influence of ligation on frictional resistance to sliding during repeated displacement. Aust Orthod J. 2006;22(2):141-6.	excluded/irrelevant	title
Keith DJ, Rinchuse DJ, Kennedy M, Zullo T. Effect of text message follow-up on patient's self-reported level of pain and anxiety. Angle Orthod. 2013;83(4):605-10.	excluded/irrelevant	title
Fansa M, Keilig L, Reimann S, Jäger A, Bourauel C. The leveling effectiveness of self-ligating and conventional brackets for complex tooth malalignments. J Orofac Orthop. 2009;70(4):285-96.	excluded/irrelevant	title
Ardila CM, Elorza-Durán A, Arrubla-Escobar D. Efficacy of CAD/CAM Technology in Interventions Implemented in Orthodontics: A Scoping Review of Clinical Trials. Biomed Res Int. 2022;2022:5310555.	excluded/irrelevant	title
Hain M, Dhopatkar A, Rock P. The effect of ligation method on friction in sliding mechanics. Am J Orthod Dentofacial Orthop. 2003;123(4):416-22.	excluded/irrelevant	title
AlSubaie M, Talic N. Comparison of the static frictional resistance and surface topography of ceramic orthodontic brackets: an in vitro study. Australasian Orthodontic Journal. 2017;33(1):24-34.	excluded/duplicate	duplicate
Pillai AR, Gangadharan A, Kumar S, Shah A. Comparison of the frictional resistance between archwire and different bracket system: An in vitro study. Journal of Pharmacy and Bioallied Sciences. 2014;6(SUPPL. 1):S150-S5.	excluded/irrelevant	title
Nishio C, da Motta AFJ, Elias CN, Mucha JN. In vitro evaluation of frictional forces between archwires and ceramic brackets. American Journal of Orthodontics and Dentofacial Orthopedics. 2004;125(1):56-64.	excluded/irrelevant	title
Turnbull NR, Birnie DJ. Treatment efficiency of conventional vs self-ligating brackets: effects of archwire size and material. Am J Orthod Dentofacial Orthop. 2007;131(3):395-9.	excluded/irrelevant	title
Yavan MA, Cingoz M, Ceylan TM, Calisir M. Incidence of orthodontic appliance failures during the COVID-19 lockdown period. Am J Orthod Dentofacial Orthop. 2022;161(1):e87-e92.	excluded/irrelevant	title
Sukontapatipark W, el-Agroudi MA, Selliseth NJ, Thunold K, Selvig KA. Bacterial colonization associated with fixed orthodontic appliances. A scanning electron microscopy study. Eur J Orthod. 2001;23(5):475-84.	excluded/ineligible intervention	full text
Harradine NWT. Self-ligating brackets: where are we now? J Orthod. 2003;30(3):262-73.	excluded/review	title
Caminiti MF, Sandor GK, Giambattistini C, Tompson B. Outcomes of the surgical exposure, bonding and eruption of 82 impacted maxillary canines. J Can Dent Assoc. 1998;64(8):572-4, 6-9.	excluded/irrelevant	title
da CMA, Gandini LG, Jr., Vianna AP, Martins RP, Jacob HB. Tooth movement rate and anchorage lost during canine retraction: A maxillary and mandibular comparison. Angle Orthod. 2019;89(4):559-65.	excluded/duplicate	duplicate
Loftus BP, Artun J, Nicholls JI, Alonzo TA, Stoner JA. Evaluation of friction during sliding tooth movement in various bracket-arch wire combinations. Am J Orthod Dentofacial Orthop. 1999;116(3):336-45.	excluded/irrelevant	title
Rucker BK, Kusy RP. Resistance to sliding of stainless steel multistranded archwires and comparison with single-stranded leveling wires. Am J Orthod Dentofacial Orthop. 2002;122(1):73-83.	excluded/irrelevant	title
Andrews LF, Andrews WA, inventors; Ortho Organizers Inc, assignee. Orthodontic bracket system patent US 08834156. 2014 Sep 16 2014.	excluded/irrelevant	abstract
Isidor F, Karring T, Nyman S, Lindhe J. New Attachment Formation on Citric-Acid Treated Roots. Journal of Periodontal Research. 1985;20(4):421-30.	excluded/irrelevant	title
da Cunha AC, Marquezan M, Ayres de Freitas AO, Nojima LI. Frictional resistance of orthodontic wires tied with 3 types of elastomeric ligatures. Brazilian Oral Research. 2011;25(6):526-30.	excluded/irrelevant	abstract
Farronato G, Maijer R, Carìa MP, Esposito L, Alberzoni D, Cacciatore G. The effect of Teflon coating on the resistance to sliding of orthodontic archwires. Eur J Orthod. 2012;34(4):410-7.	excluded/irrelevant	title
Redlich M, Mayer Y, Harari D, Lewinstein I. In vitro study of frictional forces during sliding mechanics of "reduced-friction" brackets. Am J Orthod Dentofacial Orthop. 2003;124(1):69-73.	excluded/irrelevant	title
Alsayegh E, Balut N, Ferguson DJ, Makki L, Wilcko T, Hansa I, et al. Maxillary Expansion: A Comparison of Damon Self-Ligating Bracket Therapy with MARPE and PAOO. BioMed Research International. 2022;2022.	excluded/irrelevant	title
Keith O, Jones SP, Davies EH. The influence of bracket material, ligation force and wear on frictional resistance of orthodontic brackets. British journal of orthodontics. 1993;20(2):109-15.	excluded/irrelevant	title
Othman SA, Mansor N, Saub R. Randomized controlled clinical trial of oral health-related quality of life in patients wearing conventional and self-ligating brackets. Korean journal of orthodontics. 2014;44(4):168-76.	excluded/irrelevant	title
Ctri. Conventional versus self ligating Brackets: treatment duration, root resorption and pain perception. https://trialsearchwhoint/Trial2aspx?TrialID=CTRI/2019/02/017647. 2019.	excluded/ irrelevant	title
Celar AG, Onodera K, Bertl MH, Astl E, Bantleon HP, Sato S, et al. Geometric morphometric evaluations of a randomized prospective split-mouth study on modes of ligation and reverse-curve mechanics. Orthod Craniofac Res. 2014;17(3):158-69.	excluded/irrelevant	abstract
Montasser MA, El-Bialy T, Keilig L, Reimann S, Jaeger A, Bourauel C. Force loss in archwire-guided tooth movement of conventional and self-ligating brackets. European Journal of Orthodontics. 2014;36(1):31-8.	excluded/irrelevant	title
Gómez SL, Montoya Y, Garcia NL, Virgen AL, Botero JE. Comparison of frictional resistance among conventional, active and passive selfligating brackets with different combinations of arch wires: a finite elements study. Acta Odontol Latinoam. 2016;29(2):130-6.	excluded/irrelevant	title
Paduano S, Cioffi I, Iodice G, Rapuano A, Silva R. Time efficiency of self-ligating vs conventional brackets in orthodontics: effect of appliances and ligating systems. Prog Orthod. 2008;9(2):74-80.	excluded/ineligible comparator	full text
Doherty UB, Benson PE, Higham SM. Fluoride-releasing elastomeric ligatures assessed with the in situ caries model. Eur J Orthod. 2002;24(4):371-8.	excluded/irrelevant	abstract
Rai P, Tripathi T. Prestrecher. Int J Orthod Milwaukee. 2014;25(1):39-40.	excluded/irrelevant	title
Karabin LB, Selander RW, Lavoie DC, Hunter DM, Ritucci RH, inventors; Acme Monaco Corporation, assignee. Orthodontic appliance and orthodontic treatment using the same patent US 10201402. 2019 Feb 12 2019.	excluded/ irrelevant	title
Dholakia KD, Bhat SR. Clinical efficiency of nonconventional elastomeric ligatures in the canine retraction phase of preadjusted edgewise appliance therapy: an in-vivo study. Am J Orthod Dentofacial Orthop. 2012;141(6):715-22.	excluded/irrelevant	title
Almeida MR, Futagami C, Conti AC, Oltramari-Navarro PV, Navarro Rde L. Dentoalveolar mandibular changes with self-ligating versus conventional bracket systems: A CBCT and dental cast study. Dental Press J Orthod. 2015;20(3):50-7.	excluded/ irrelevant	abstract
Tada Y, Hayakawa T, Nakamura Y. Load-Deflection and Friction Properties of PEEK Wires as Alternative Orthodontic Wires. 2017;10(8).	excluded/irrelevant	title
Pandis N, Papaioannou W, Kontou E, Nakou M, Makou M, Eliades T. Salivary Streptococcus mutans levels in patients with conventional and self-ligating brackets. Eur J Orthod. 2010;32(1):94-9.	excluded/ineligible comparator	full text

Martu MA, Danila CE, Luchian I, Solomon SM, Martu I, Foia L, et al. Effect of Laser Therapy on Gingivitis during Orthodontic Treatment. International Journal of Medical Dentistry. 2017;21(4):284-9.	excluded/irrelevant	title
Aykut-Yetkiner A, Eden E, Ertuğrul F, Ergin E, Ateş M. Antibacterial efficacy of prophylactic ozone treatment on patients with fixed orthodontic appliances. Acta Odontol Scand. 2013;71(6):1620-4.	excluded/irrelevant	title
Angolkar PV, Kapila S, Duncanson MG, Nanda RS. Evaluation of Friction between Ceramic Brackets and Orthodontic Wires of 4 Alloys. American Journal of Orthodontics and Dentofacial Orthopedics. 1990;98(6):499-506.	excluded/irrelevant	title
Gyawali R, Pokharel PR, Giri J. Emergency appointments in orthodontics. Apos Trends in Orthodontics. 2019;9(1):40-3.	excluded/irrelevant	title
Al-Haifi HAA, Ishaq RAA, Al-Hammadi MSA. Salivary pH changes under the effect of stainless steel versus elastomeric ligatures in fixed orthodontic patients: a single-center, randomized controlled clinical trial. BMC Oral Health. 2021;21(1):544.	excluded/ineligible outcome	full text
Baturina O, Tufekci E, Guney-Altay O, Khan SM, Wnek GE, Lindauer SJ. Development of a sustained fluoride delivery system. The Angle orthodontist. 2010;80(6):1129-35.	excluded/irrelevant	title
da Silva RR, Pereira GO, Macari S, Barbosa JA, Basting RT. Friction evaluation of an elastic chain positioned under or over the wire in self-ligating brackets. APOS Trends in Orthodontics. 2021;11(3):183-90.	excluded/ irrelevant	title
Yassir YA, McIntyre GT, Bearn DR. Variation in bracket slot sizes, ligation methods and prescriptions: UK national survey. Int Orthod. 2019;17(3):519-28.	excluded/irrelevant	full text
O'Dwyer JJ, Tinsley D, Benson PE. The effect of stretching on the release of fluoride from fluoridated elastomeric ligatures. American Journal of Orthodontics and Dentofacial Orthopedics. 2005;128(4):471-6.	excluded/irrelevant	title
Lee SJ, Kho HS, Lee SW, Yang WS. Experimental salivary pellicles on the surface of orthodontic materials. Am J Orthod Dentofacial Orthop. 2001;119(1):59-66.	excluded/irrelevant	title
Downing A, McCabe J, Gordon P. A study of frictional forces between orthodontic brackets and archwires. British journal of orthodontics. 1994;21(4):349-57.	excluded/irrelevant	title
Nahás-Scocate ACR, Neves MB, de Souza LT, de Cerqueira Kasaz A, Listik E, da Silva HDP, et al. An in vitro assessment of the influences of different wire materials and bracket systems when correcting dental crowding. J Mater Sci Mater Med. 2020;31(11):108.	excluded/irrelevant	title
Major PW, Toogood RW, Badawi HM, Carey JP, Seru S. Effect of wire size on maxillary arch force/couple systems for a simulated high canine malocclusion. J Orthod. 2014;41(4):285-91.	excluded/irrelevant	title
Qiao YQ, Zhu LS, Cui SJ, Zhang T, Yang RL, Zhou YH. Local Administration of Stem Cells from Human Exfoliated Primary Teeth Attenuate Experimental Periodontitis in Mice. Chin J Dent Res. 2019;22(3):157-63.	excluded/irrelevant	title
Deguchi T, Imai M, Sugawara Y, Ando R, Kushima K, Takano-Yamamoto T. Clinical evaluation of a low-friction attachment device during canine retraction. Angle Orthod. 2007;77(6):968-72.	excluded/irrelevant	title
Quintão CC, Cal-Neto JP, Menezes LM, Elias CN. Force-deflection properties of initial orthodontic archwires. World J Orthod. 2009;10(1):29-32.	excluded/irrelevant	title
Barrett RD, Bishara SE, Quinn JK. Biodegradation of Orthodontic Appliances .1. Biodegradation of Nickel and Chromium Invitro. American Journal of Orthodontics and Dentofacial Orthopedics. 1993;103(1):8-14.	excluded/irrelevant	title
Darling S, Darling J, inventorsElastomeric orthodontic ligator patent US 07204691. 2007 Apr 17 2007.	excluded/irrelevant	full text
Suryawanshi GR, Sundareswaran S, Philip K, Kumar S. In vitro evaluation of different methods of ligation on friction in sliding mechanics. Orthodontics (Chic). 2013;14(1):e102-9.	excluded/irrelevant	title
Radica N, Dukic P, Vidovic N, Gabric D, Skaricic J, Mestrovic S. Debonding of ceramics self-ligating brackets by diode laser. Lasers in surgery and medicine. 2017;49:44-.	excluded/irrelevant	title
Tochigi K, Oda S, Arai K. Influences of archwire size and ligation method on the force magnitude delivered by nickel-titanium alloy archwires in a simulation of mandibular right lateral incisor linguoversion. Dent Mater J. 2015;34(3):388-93.	excluded/irrelevant	title
Andregg M, Kaminski E, Forbes D. Risk assessment of extractable residual glutaraldehyde from orthodontic elastic ligatures following sterilization in a 3.2% glutaraldehyde solution. Northwest Dent Res. 1997;7(2):7-15.	excluded/irrelevant	title
Le Gall M, Bachet C, Dameron C. The time needed to refit an orthodontic wire: influence of the attachments. Int Orthod. 2014;12(4):431-42.	excluded/irrelevant	title
Uribe F, Davoody L, Mehr R, Jayaratne YSN, Almas K, Sobue T, et al. Efficiency of piezotome-corticision assisted orthodontics in alleviating mandibular anterior crowding-a randomized clinical trial. Eur J Orthod. 2017;39(6):595-600.	excluded/irrelevant	abstract
AlSubaie M, Talic N, Khawatmi S, Alobeid A, Bourauel C, El-Bialy T. Study of force loss due to friction comparing two ceramic brackets during sliding tooth movement. J Orofac Orthop. 2016;77(5):334-40.	excluded/irrelevant	title
Tochigi K, Saze N, Arai K. Impact of passive self-ligation and conventional elastic ligation on orthodontic force in the simulation of a mandibular lateral incisor linguoversion. Am J Orthod Dentofacial Orthop. 2020;157(3):320-8.	excluded/irrelevant	title
Huntley PN. Amodified over-tie for the ligation of Incognito™ lingual fixed appliances. Journal of Orthodontics. 2013;40(3):244-8.	excluded/duplicate	duplicate
Tecco S, Festa F, Caputi S, Traini T, Di Iorio D, D'Attilio M. Friction of conventional and self-ligating brackets using a 10 bracket model. Angle Orthod. 2005;75(6):1041-5.	excluded/irrelevant	title
Gölz L, Knickenberg AC, Keilig L, Reimann S, Papageorgiou SN, Jäger A, et al. Nickel ion concentrations in the saliva of patients treated with self-ligating fixed appliances: a prospective cohort study. J Orofac Orthop. 2016;77(2):85-93.	excluded/irrelevant	title
Guarnieri R, Bertoldo S, Cassetta M, Altieri F, Grenga C, Vichi M, et al. Periodontal results of different therapeutic approaches (open vs. closed technique) and timing evaluation (< 2 year vs. > 2 year) of palatal impacted canines: a systematic review. Bmc Oral Health. 2021;21(1).	excluded/irrelevant	title
Forsberg CM, Brattström V, Malmberg E, Nord CE. Ligature wires and elastomeric rings: two methods of ligation, and their association with microbial colonization of Streptococcus mutans and lactobacilli. Eur J Orthod. 1991;13(5):416-20.	included	
González-Sáez A, Antonio-Zancajo L, Montero J, Albaladejo A, Melo M, Garcovich D, et al. The influence of friction on design of the type of bracket and its relation to ohrqol in patients who use multi-bracket appliances: A randomized clinical trial. Medicina (Lithuania). 2021;57(2):1-10.	excluded/irrelevant	title
Bai MP, Vaz AC. "Comparative evaluation of surface modified elastomeric ligatures for microbial colonization": An in vivo study. Indian J Dent Res. 2015;26(2):180-5.	excluded/ineligible outcome	full text
Lo Giudice A, Portelli M, Militi A, Spinuzza P, Bellocchio AM, Nucera R, et al. Is static friction affected by aging and amount of elastomeric ligatures in orthodontic sliding mechanics? An in-vitro investigation. Journal of biological regulators and homeostatic agents. 2018;32(2 Suppl. 2):67-73.	excluded/irrelevant	title
Akaike S, Hayakawa T, Kobayashi D, Aono Y, Hirata A, Hiratsuka M, et al. Reduction in static friction by deposition of a homogeneous diamond-like carbon (DLC) coating on orthodontic brackets. Dental Materials Journal. 2015;34(6):888-95.	excluded/irrelevant	title
Čelar A, Schedlberger M, Dörfler P, Bertl M. Systematic review on self-ligating vs. conventional brackets: initial pain, number of visits, treatment time. J Orofac Orthop. 2013;74(1):40-51.	excluded/irrelevant	title
Mikulewicz M, Wołowiec P, Loster BW, Chojnacka K. Do soft drinks affect metal ions release from orthodontic appliances? 2015;31:74-7.	excluded/irrelevant	title
Gurgel JD, Kerr S, Powers JM, LeCrone V. Force-deflection properties of superelastic nickel-titanium archwires. American Journal of Orthodontics and Dentofacial Orthopedics. 2001;120(4):378-82.	excluded/irrelevant	title
Abdala-Junior R, No-Cortes J, Arita ES, Ackerman JL, da Silva RLB, Kim JH, et al. Influence of receiver bandwidth on MRI artifacts caused by orthodontic brackets composed of different alloys. Imaging science in dentistry. 2021;51(4):413-9.	excluded/irrelevant	title
Trevisi H, Bergstrand F. The SmartClip Self-Ligating Appliance System. Seminars in Orthodontics. 2008;14(1):87-100.	excluded/irrelevant	title
Turrel B, Valran V, Gebeile-Chauty S. [Biomechanics of mini-implants : analysis of the adverse effects of four clinical situations and proposed resolutions]. Orthod Fr. 2021;92(2):195-214.	excluded/non-english language	abstract
Aljabaa AH, Almoammar K, Al-Kharboush G, Al-Dayel RM, Alsaloom NS, Albarakati S. Colour preferences of elastic ligatures among orthodontic patients. Journal of Clinical and Diagnostic Research. 2021;15(1):ZC01-ZC4.	excluded/irrelevant	title
Nieto Uribe M, Barrera Chaparro JP, Gonzáles Cáceres EJ, Parra Mazo IL, Rodríguez Quijada AC. Comparación de la resistencia al deslizamiento en brackets deautoligado y brackets convencionales ligados con ligadura elastomérica convencional y ligaduras de baja fricción. Rev Fac Odontol Univ Antioq. 2012;23(2):192-206.	excluded/irrelevant	title
Kasuya S, Nagasaka S, Hanyuda A, Ishimura S, Hirashita A. The effect of ligation on the load deflection characteristics of nickel titanium orthodontic wire. Eur J Orthod. 2007;29(6):578-82.	excluded/irrelevant	title
Dragomirescu AO, Bencze MA, Vasilache A, Teodorescu E, Albu CC, Popoviciu NO, et al. Reducing Friction in Orthodontic Brackets: A Matter of Material or Type of Ligation Selection? In-Vitro Comparative Study. Materials. 2022;15(7).	excluded/irrelevant	title
Tctr. Effectiveness of a combined Horizontal-Charters-modified Bass brushing technique and dietary advice in reducing plaque pathogenicity in patients with stainless steel and elastomeric ligatures: a randomized clinical trial. https://trialsearchwhoint/Trial2aspx?TrialID=TCTR20220221003. 2022.	excluded/ irrelevant	title
Gioka C, Eliades T. Materials-induced variation in the torque expression of preadjusted appliances. Am J Orthod Dentofacial Orthop. 2004;125(3):323-8.	excluded/irrelevant	title
Koike F, Maruo H, Lacerda-Santos R, Pithon MM, Tanaka OM. Mechanical properties of orthodontic wires on ceramic brackets associated with low friction ligatures associados com ligaduras de baixa fricção. Revista de Odontologia da UNESP. 2017;46(3):125-30.	excluded/irrelevant	title
Migliorati M, Poggio D, Drago S, Lagazzo A, Stradi R, Barberis F, et al. Torque expression of a customized lingual appliance according to different elastomeric ligatures over time: an in vitro study. Annali Di Stomatologia. 2018;9(2):59-64.	excluded/irrelevant	title
Dot G, Licha R, Goussard F, Sansalone V. Clinical and numerical study of a statically determinate lingual mechanism for orthodontic tooth displacement. Computer Methods in Biomechanics and Biomedical Engineering. 2020;23(SUPPL 1):S85-S7.	excluded/ irrelevant	title
Hiroce M, Fernandes DJ, Elias CN, Miguel JA. Sliding resistance of polycarbonate self-ligating brackets and stainless steel esthetic archwires. Prog Orthod. 2012;13(2):148-53.	excluded/irrelevant	title
Ding P, Lin J-x, Zhou Y-h. [Development and preliminary application of orthodontic friction dynamic testing apparatus]. Beijing Da Xue Xue Bao Yi Xue Ban. 2009;41(3):319-23.	excluded/irrelevant	abstract
Taylor NG, Ison K. Frictional resistance between orthodontic brackets and archwires in the buccal segments. Angle Orthod. 1996;66(3):215-22.	excluded/irrelevant	title
Becker A, Chaushu G, Chaushu S. Analysis of failure in the treatment of impacted maxillary canines. Am J Orthod Dentofacial Orthop. 2010;137(6):743-54.	excluded/irrelevant	title
Jiang RP, Fu MK. [Non-extraction treatment with self-ligating and conventional brackets]. Zhonghua Kou Qiang Yi Xue Za Zhi. 2008;43(8):459-63.	excluded/irrelevant	title
Fleming PS, Dibiase AT, Sarri G, Lee RT. Pain experience during initial alignment with a self-ligating and a conventional fixed orthodontic appliance system. A randomized controlled clinical trial. Angle Orthod. 2009;79(1):46-50.	excluded/irrelevant	title
Bertelè M, Minniti PP, Dalessandri D, Bonetti S, Visconti L, Paganelli C. A computer-guided minimally-invasive technique for orthodontic forced eruption of impacted canines. Minerva Stomatol. 2016;65(3):176-84.	excluded/ irrelevant	title
Ziuchkovski JP, Fields HW, Johnston WM, Lindsey DT. Assessment of perceived orthodontic appliance attractiveness. American Journal of Orthodontics and Dentofacial Orthopedics. 2008;133(4):S68-S78.	excluded/irrelevant	title
Kusy RP, Whitley JQ, de Araújo Gurgel J. Comparisons of surface roughnesses and sliding resistances of 6 titanium-based or TMA-type archwires. Am J Orthod Dentofacial Orthop. 2004;126(5):589-603.	excluded/irrelevant	title
Kessler P, Türp JC. Influence of Coca-Cola on orthodontic materials. A systematic review. Swiss Dent J. 2020;130(12):983-93.	excluded/irrelevant	title
Sifakakis I, Pandis N, Makou M, Katsaros C, Eliades T, Bourauel C. A comparative assessment of forces and moments generated by lingual and conventional brackets. Eur J Orthod. 2013;35(1):82-6.	excluded/irrelevant	title
Kassam SK, Stoops FR. Are clear aligners as effective as conventional fixed appliances? Evid Based Dent. 2020;21(1):30-1.	excluded/irrelevant	title
Khamatkar A, Sonawane S, Narkhade S, Gadhiya N, Bagade A, Soni V, et al. Effects of different ligature materials on friction in sliding mechanics. J Int Oral Health. 2015;7(5):34-40.	excluded/irrelevant	title
Ctri. To compare lingual bonded retainer post orthodontically. https://trialsearchwhoint/Trial2aspx?TrialID=CTRI/2019/05/019136. 2019.	excluded/ irrelevant	title
Pasha A, Vishwakarma S, Narayan A, Vinay K, Shetty SV, Roy PP. Comparison of Frictional Forces Generated by a New Ceramic Bracket with the Conventional Brackets using Unconventional and Conventional Ligation System and the Self-ligating Brackets: An In Vitro Study. J Int Oral Health. 2015;7(9):108-13.	excluded/irrelevant	title
Conley RS, Boyd SB, Legan HL, Jernigan CC, Starling C, Potts C. Treatment of a patient with multiple impacted teeth. Angle Orthod. 2007;77(4):735-41.	excluded/ irrelevant	title
Patni V, Dmello K, Wadhwa J, Reddy MSR, Singh A. An in-vitro evaluation of microbial adhesion on different types of orthodontic brackets. Journal of Clinical and Diagnostic Research. 2021;15(6):ZC01-ZC5.	excluded/ irrelevant	title

Mattick CR, Mitchell L, Chadwick SM, Wright J. Fluoride-releasing elastomeric modules reduce decalcification: a randomized controlled trial. J Orthod. 2001;28(3):217-9.	excluded/irrelevant	title
Thorstenson GA, Kusy RP. Effect of archwire size and material on the resistance to sliding of self-ligating brackets with second-order angulation in the dry state. Am J Orthod Dentofacial Orthop. 2002;122(3):295-305.	excluded/irrelevant	title
Camporesi M, Bulhoes Galvão M, Tortamano A, Dominguez GC, Defraia N, Defraia E, et al. Ceramic brackets and low friction : A possible synergy in patients requiring multiple MRI scanning. J Orofac Orthop. 2016;77(3):214-23.	excluded/irrelevant	title
Harradine N. The History and Development of Self-Ligating Brackets. Seminars in Orthodontics. 2008;14(1):5-18.	excluded/irrelevant	title
Esteves T, Salvatore Freitas KM, Vaz de Lima D, Cotrin P, Cançado RH, Valarelli FP, et al. Comparison of WALA ridge and dental arch dimensions changes after orthodontic treatment using a passive self-ligating system or conventional fixed appliance. Indian J Dent Res. 2019;30(3):386-92.	excluded/irrelevant	title
Hain M, Dhopatkar A, Rock P. A comparison of different ligation methods on friction. Am J Orthod Dentofacial Orthop. 2006;130(5):666-70.	excluded/ineligible outcome	full text
Sifakakis I, Pandis N, Makou M, Eliades T, Katsaros C, Bourauel C. Torque expression of 0.018 and 0.022 inch conventional brackets. Eur J Orthod. 2013;35(5):610-4.	excluded/duplicate	duplicate
Alqahtani ND. Assessment on the Precision of the Orthodontic Bracket Slot Dimensions Using Micro-computed Tomography (Micro-CT). The journal of contemporary dental practice. 2021;22(1):27-33.	excluded/irrelevant	title
Kusy RP, Whitley JQ. Frictional resistances of metal-lined ceramic brackets versus conventional stainless steel brackets and development of 3-D friction maps. Angle Orthodontist. 2001;71(5):364-74.	excluded/irrelevant	title
Mølsted K. [Ceramic brackets]. Tandlaegebladet. 1992;96(1):6-11.	excluded/irrelevant	title
Nct. A RCT to Compare OHRQoL Outcome for SLB and CB. https://clinicaltrialsgov/show/NCT02084277. 2014.	excluded/irrelevant	title
Liu X, Ding P, Lin J. Effects of bracket design on critical contact angle. Angle Orthod. 2013;83(5):877-84.	excluded/irrelevant	title
Chhibber A, Agarwal S, Yadav S, Kuo CL, Upadhyay M. Which orthodontic appliance is best for oral hygiene? A randomized clinical trial. Am J Orthod Dentofacial Orthop. 2018;153(2):175-83.	excluded/ineligible comparator	full text
Ctri. A clinical try to compare the changes in the tooth and bone position when using a new type of braces using very light forces and a conventional braces used for treating patients with irregular teeth-A 3 Dimensional study. https://trialsearchwhoint/Trial2aspx?TrialID=CTRI/2020/05/025028. 2020.	excluded/irrelevant	title
Hegele J, Seitz L, Claussen C, Baumert U, Sabbagh H, Wichelhaus A. Clinical effects with customized brackets and CAD/CAM technology: a prospective controlled study. Prog Orthod. 2021;22(1):40.	excluded/irrelevant	title
Prososki RR, Bagby MD, Erickson LC. Static Frictional Force and Surface-Roughness of Nickel-Titanium Arch Wires. American Journal of Orthodontics and Dentofacial Orthopedics. 1991;100(4):341-8.	excluded/irrelevant	title
Yoon SH, inventor; Biocetec Co Ltd, assignee. Bracket for self-ligating correction patent US 11395721. 2022 Jul 26 2022.	excluded/ineligible comparator	full text
Isrctn. A randomised controlled trial comparing conventional, active and passive self-ligating orthodontic bracket systems. https://trialsearchwhoint/Trial2aspx?TrialID=ISRCTN51381850. 2007.	excluded/ irrelevant	title
Mencattelli M, Donati E, Cultrone M, Stefanini C. Novel universal system for 3-dimensional orthodontic force-moment measurements and its clinical use. Am J Orthod Dentofacial Orthop. 2015;148(1):174-83.	excluded/irrelevant	title
Lefebvre C, Saadaoui H, Olive JM, Renaudin S, Jordana F. Variability of slot size in orthodontic brackets. Clin Exp Dent Res. 2019;5(5):528-33.	excluded/irrelevant	title
Jian F, Lai W, Furness S, McIntyre GT, Millett DT, Hickman J, et al. Initial arch wires for tooth alignment during orthodontic treatment with fixed appliances. Cochrane Database Syst Rev. 2013;2013(4):Cd007859.	excluded/irrelevant	title
Lombardo L, Wierusz W, Toscano D, Lapenta R, Kaplan A, Siciliani G. Frictional resistance exerted by different lingual and labial brackets: An in vitro study. Progress in Orthodontics. 2013;14(1):1-10.	excluded/duplicate	duplicate
Oliveira AS, Kaizer MR, Salgado VE, Soldati DC, Silva RC, Moraes RR. Influence of whitening and regular dentifrices on orthodontic clear ligature color stability. J Esthet Restor Dent. 2015;27 Suppl 1:S58-64.	excluded/irrelevant	title
Contaldo M, Lucchese A, Lajolo C, Rupe C, Di Stasio D, Romano A, et al. The oral microbiota changes in orthodontic patients and effects on oral health: An overview. Journal of Clinical Medicine. 2021;10(4):1-13.	excluded/review	full text
Villaman-Santacruz H, Torres-Rosas R, Acevedo-Mascarúa AE, Argueta-Figueroa L. Root resorption factors associated with orthodontic treatment with fixed appliances: A systematic review and meta-analysis. Dent Med Probl. 2022;59(3):437-50.	excluded/irrelevant	abstract
Salandin M. Técnicas autoligantes mecánica de fuerzas ligeras? Ortodoncia. 2011;74(149):56-66.	excluded/irrelevant	title
Ravishankar M, Duraisamy S, Rajaram K, Kannan R, Sivakumar N. Frictional Resistance of Non Coated and Epoxy Coated Superelastic NiTi Wires used for Aligning in Three Types of Brackets. Journal of Clinical and Diagnostic Research. 2022;16(6):ZC40-ZC3.	excluded/irrelevant	title
Nct. Biological Anchorage With Selective Micro-osteoperforations (MOPs) in Canine Distalization. https://clinicaltrialsgov/show/NCT03741504. 2018.	excluded/ irrelevant	title
Pliska BT, Beyer JP, Larson BE. A comparison of resistance to sliding of self-ligating brackets under an increasing applied moment. Angle Orthod. 2011;81(5):794-9.	excluded/irrelevant	title
Cantarelli MC, Godoi APTd, Sinhoreti MAC, Neves JG, Santos EA, Correr-Sobrinho L, et al. Effect of horizontal slot of maxillary canines' brackets with varying wire angulations - An in vitro study. Brazilian dental journal. 2022;33(5):55-63.	excluded/irrelevant	title
Rhoden FK, Maltagliati L, de Castro Ferreira Conti AC, Almeida-Pedrin RR, Filho LC, de Almeida Cardoso M. Cone Beam Computed Tomography-based Evaluation of the Anterior Teeth Position Changes obtained by Passive Self-ligating Brackets. J Contemp Dent Pract. 2016;17(8):623-9.	excluded/irrelevant	title
Carneiro GKM, Roque JA, Segundo ASG, Suzuki H. Evaluation of stiffness and plastic deformation of active ceramic self-ligating bracket clips after repetitive opening and closure movements. Dental press journal of orthodontics. 2015;20(4):45-50.	excluded/irrelevant	title
St George G, Donachie MA. Case report: orthodontic separators as periodontal ligatures in periodontal bone loss. Eur J Prosthodont Restor Dent. 2002;10(3):97-9.	excluded/case report	full text
Nair SV, Padmanabhan R, Janardhanam P. Evaluation of the effect of bracket and archwire composition on frictional forces in the buccal segments. Indian J Dent Res. 2012;23(2):203-8.	excluded/irrelevant	title
Moresca R, Vigorito JW. Avaliação in vitro da força produzida por fios de ligadura utilizados como lacebacks. Ortodontia. 2005;38(3):212-8.	excluded/irrelevant	title
Abass AA, Abed RA. Effect of Composition of Bracket on the Accumulation of Plaque in the Oral Cavity : In Vivo Study. Biochemical and Cellular Archives. 2020;20(Suppl. 2):3779-82.	excluded/ineligible intervention	full text
Adams DM, Powers JM, Asgar K. Effects of Brackets and Ties on Stiffness of an Arch Wire. American Journal of Orthodontics and Dentofacial Orthopedics. 1987;91(2):131-6.	excluded/irrelevant	title
Mummolo S, Marchetti E, Giuca MR, Gallusi G, Tecco S, Gatto R, et al. In-office bacteria test for a microbial monitoring during the conventional and self-ligating orthodontic treatment. Head Face Med. 2013;9:7.	excluded/ineligible comparator	full text
Ribeiro AA, Mattos CT, Ruellas AC, Araújo MT, Elias CN. In vivo comparison of the friction forces in new and used brackets. Orthodontics (Chic). 2012;13(1):e44-50.	excluded/irrelevant	title
Otasevic M, Naini FB, Gill DS, Lee RT. Prospective randomized clinical trial comparing the effects of a masticatory bite wafer and avoidance of hard food on pain associated with initial orthodontic tooth movement. Am J Orthod Dentofacial Orthop. 2006;130(1):6.e9-15.	excluded/irrelevant	title
Mirzakouchaki B. Asymmetrical O-ring ligation. J Clin Orthod. 2008;42(2):100.	excluded/irrelevant	title
Nct. Effects Of Low Level Laser Therapy On Tooth Movement,Treatment Related Complications Of Gingivitis,Periodontitis And Pain In Fixed Orthodontic Patients. https://clinicaltrialsgov/show/NCT04230096. 2020.	excluded/ irrelevant	title
Pacheco MR, Oliveira DD, Smith Neto P, Jansen WC. Avaliação do atrito em braquetes autoligáveis submetidos à mecânica de deslizamento: um estudo in vitro. Dental press j orthod (Impr). 2011;16(1):107-15.	excluded/irrelevant	title
Cattaneo PM, Salih RA, Melsen B. Labio-lingual root control of lower anterior teeth and canines obtained by active and passive self-ligating brackets. Angle Orthodontist. 2013;83(4):691-7.	excluded/irrelevant	abstract
Maia LH, Lopes Filho H, Ruellas AC, Araújo MT, Vaitsman DS. Corrosion behavior of self-ligating and conventional metal brackets. Dental Press J Orthod. 2014;19(2):108-14.	excluded/irrelevant	title
Oz AA, Arici N, Arici S. The clinical and laboratory effects of bracket type during canine distalization with sliding mechanics. Angle Orthod. 2012;82(2):326-32.	excluded/irrelevant	title
Xiaowei L, Luyi Y, Huifang Z, Yan D, Qi W, Wei J, et al. [Comparison of friction force between Lock-loose bracket and traditional bracket]. Hua Xi Kou Qiang Yi Xue Za Zhi. 2014;32(6):570-4.	excluded/irrelevant	title
Uzuner FD, Kaygisiz E, Cankaya ZT. Effect of the bracket types on microbial colonization and periodontal status. Angle Orthod. 2014;84(6):1062-7.	excluded/ineligible intervention	full text
Deshpande A, Srinivas N, Kumar KK, Mapare S. Comparison of Opal self-ligating brackets with manually ligating brackets. J Contemp Dent Pract. 2012;13(4):494-503.	excluded/irrelevant	title
Ctri. An evaluation of the efficacy of labial versus lingual bonded retainers: an in-vivo study. https://trialsearchwhoint/Trial2aspx?TrialID=CTRI/2022/06/043125. 2022.	excluded/irrelevant	title
Nct. Changes in Pulpal Blood Flow Between Conventional and Self-ligating Fixed Orthodontic Brackets During Leveling and Alignment Stage. https://clinicaltrialsgov/show/NCT03780764. 2018.	excluded/ irrelevant	title
Badawi HM, Toogood RW, Carey JPR, Heo G, Major PW. Torque expression of self-ligating brackets. American Journal of Orthodontics and Dentofacial Orthopedics. 2008;133(5):721-8.	excluded/irrelevant	title
Reimann S, Bourauel C, Weber A, Dirk C, Lietz T. Friction behavior of ceramic injection-molded (CIM) brackets. J Orofac Orthop. 2016;77(4):262-71.	excluded/irrelevant	title
Bortoly TG, Guerrero AP, Rached RN, Tanaka O, Guariza-Filho O, Rosa EAR. Sliding resistance with esthetic ligatures: An in-vitro study. American Journal of Orthodontics and Dentofacial Orthopedics. 2008;133(3).	excluded/irrelevant	title
Ctri. Evaluation of three different Nickel Titanium archwires during initial phase of comprehensive orthodontic treatmentÃ¢??A randomized controlled clinical trial. https://trialsearchwhoint/Trial2aspx?TrialID=CTRI/2019/06/019647. 2019.	excluded/ irrelevant	title
Pollit DJ. Twist ligation: a technique to reduce archwire binding. Journal of clinical orthodontics : JCO. 1996;30(3):150-3.	excluded/duplicate	duplicate
Lawal OA, Temisanren OT, Adeyemi AT. In vivo and in vitro evaluation of the mechanical properties of orthodontic elastomeric ligatures. Journal of the World Federation of Orthodontists. 2019;8(2):68-72.	excluded/ineligible outcome	full text
Aristizábal JF, Martínez-Smit R, Díaz C, Pereira Filho VA. Surgery-first approach with 3D customized passive self-ligating brackets and 3D surgical planning: Case report. Dental Press J Orthod. 2018;23(3):47-57.	excluded/irrelevant	title
Articolo LC, Kusy K, Saunders CR, Kusy RP. Influence of ceramic and stainless steel brackets on the notching of archwires during clinical treatment. Eur J Orthod. 2000;22(4):409-25.	excluded/irrelevant	title
Rampon FB, Nóbrega C, Bretos JL, Arsati F, Jakob S, Jimenez-Pellegrin MC. Profile of the orthodontist practicing in the State of São Paulo--part 2. Dental Press J Orthod. 2013;18(1):32.e1-6.	excluded/irrelevant	title
Chung M, Nikolai RJ, Kim KB, Oliver DR. Third-order torque and self-ligating orthodontic bracket-type effects on sliding friction. Angle Orthod. 2009;79(3):551-7.	excluded/irrelevant	title
Choi S, Lee S, Cheong Y, Park KH, Park HK, Park YG. Ultrastructural effect of self-ligating bracket materials on stainless steel and superelastic NiTi wire surfaces. Microsc Res Tech. 2012;75(8):1076-83.	excluded/irrelevant	title
Gameiro GH, Nouer DF, Cenci MS, Cury JA. Enamel demineralization with two forms of archwire ligation investigated using an in situ caries model—a pilot study. The European Journal of Orthodontics. 2009 Oct 1;31(5):542-6.	included	
Hirunyapanun N, Saengfai NN, Chintavalakorn R, Joachakarasiri P. The study of frictional force in self-ligating orthodontic brackets. International Journal of Mechanical Engineering and Robotics Research. 2017;6(4):290-5.	excluded/irrelevant	title
Gopalakrishnan U, Felicita S, Ronald B, Appavoo E, Patil S. Microbial Corrosion in Orthodontics. J Contemp Dent Pract. 2022;23(6):569-71.	excluded/irrelevant	title
Loreille JP. [Corrosion and calculus. How can the wire/bracket slide mechanics be improved?]. Orthod Fr. 2002;73(1):71-81.	excluded/irrelevant	title
O'Brien K. Longer treatment times with self-ligated orthodontic brackets. Evid Based Dent. 2014;15(3):92.	excluded/irrelevant	title
Postlethwaite KM. Advances in fixed appliance design and use: 1. Brackets and archwires. Dent Update. 1992;19(7):276-8, 80.	excluded/irrelevant	title
Huang Y, Keilig L, Rahimi A, Reimann S, Eliades T, Jäger A, et al. Numeric modeling of torque capabilities of self-ligating and conventional brackets. Am J Orthod Dentofacial Orthop. 2009;136(5):638-43.	excluded/irrelevant	title

Vaughan JL, Duncanson MG, Jr., Nanda RS, Currier GF. Relative kinetic frictional forces between sintered stainless steel brackets and orthodontic wires. Am J Orthod Dentofacial Orthop. 1995;107(1):20-7.	excluded/irrelevant	title
Skilbeck MG, Mei L, Mohammed H, Cannon RD, Farella M. The effect of ligation methods on biofilm formation in patients undergoing multi-bracketed fixed orthodontic therapy - A systematic review. Orthod Craniofac Res. 2022;25(1):14-30.	excluded/review	full text
Yang X, Xue C, He Y, Zhao M, Luo M, Wang P, et al. Transversale Veränderungen, Lückenschluss und Wirksamkeit von selbstligierenden vs. konventionellen Brackets : Ein quantitatives systematisches Review. Journal of orofacial orthopedics = Fortschritte der Kieferorthopadie : Organ/official journal Deutsche Gesellschaft fur Kieferorthopadie. 2018;79(1):1-10.	excluded/non-english language	title
Sakamoto T, Sakamoto S, Harazaki M, Isshiki Y, Yamaguchi H. Orthodontic treatment for jaw deformities in cleft lip and palate patients with the combined use of an external-expansion arch and a facial mask. Bull Tokyo Dent Coll. 2002;43(4):223-9.	excluded/irrelevant	title
Camporesi M, Baccetti T, Franchi L. Forces released by esthetic preadjusted appliances with low-friction and conventional elastomeric ligatures. American Journal of Orthodontics and Dentofacial Orthopedics. 2007;131(6):772-5.	excluded/irrelevant	title
Ben Rejeb Jdir S, Tobji S, Turki W, Dallel I, Khedher N, Ben Amor A. [Brackets and friction in orthodontics: experimental study]. Orthod Fr. 2015;86(3):255-64.	excluded/irrelevant	title
Vincent-Bugnas S, Borsa L, Gruss A, Lupi L. Prioritization of predisposing factors of gingival hyperplasia during orthodontic treatment: the role of amount of biofilm. BMC Oral Health. 2021;21(1):84.	excluded/irrelevant	abstract
Manni A, Lupini D, Cozzani M. Bone-anchored intermaxillary elastics in an asymmetric Class II malocclusion: A case report. Int Orthod. 2017;15(2):263-77.	excluded/ irrelevant	title
Nct. How Often Should We See Our Orthodontic Patients. https://clinicaltrialsgov/show/NCT02918240. 2016.	excluded/ irrelevant	title
Benson PE, Douglas CW, Martin MV. Fluoridated elastomers: effect on the microbiology of plaque. Am J Orthod Dentofacial Orthop. 2004;126(3):325-30.	excluded/ineligible intervention	full text
Sfondrini MF, Gandini P, Castroflorio T, Garino F, Mergati L, D'Anca K, et al. Buccolingual Inclination Control of Upper Central Incisors of Aligners: A Comparison with Conventional and Self-Ligating Brackets. Biomed Res Int. 2018;2018:9341821.	excluded/irrelevant	title
Pandis N, Eliades T, Bourauel C. Comparative assessment of forces generated during simulated alignment with self-ligating and conventional brackets. Eur J Orthod. 2009;31(6):590-5.	excluded/irrelevant	title
Pattan SK, Peddu R, Bandaru SK, Lanka D, Mallavarapu K, Pathan AB. Efficacy of Super Slick elastomeric modules in reducing friction during sliding: a comparative in vitro study. J Contemp Dent Pract. 2014;15(5):543-51.	excluded/irrelevant	title
Jung MH. Factors influencing treatment efficiency. Angle Orthod. 2021;91(1):1-8.	excluded/irrelevant	title
Herman RJ, Currier GF, Miyake A. Mini-implant anchorage for maxillary canine retraction: a pilot study. Am J Orthod Dentofacial Orthop. 2006;130(2):228-35.	excluded/irrelevant	title
Pacheco MR, Oliveira DD, Neto PS, Jansen WC. Evaluation of friction in self-ligating brackets subjected to sliding mechanics: An in vitro study. Dental Press Journal of Orthodontics. 2011;16(1):107-15.	excluded/irrelevant	title
Kilic N, Oktay H. Orthodontic Intervention to Impacted and Transposed Lower Canines. Case Reports in Dentistry. 2017;2017.	excluded/irrelevant	title

Appendix 4. Sensitivity analysis according to the design of included studies.

	Non-randomized studies		Randomized studies		
**Outcome**	**Studies**	**Effect (95% CI)**	**Studies**	**Effect (95% CI)**	P_{SG}
Plaque Index	5	SMD 0.48 (-0.03, 1.00)	0	-	-
Gingival Index	2	MD 0.01 (-0.14, 0.16)	0	-	-
PPD	2	MD 0 (-0.17, 0.16)	0	-	-
Bacterial count	2	SMD 0.47 (-0.57, 1.51)	1	SMD 0.27 (-0.23, 0.77)	0.45
S. mutans count	2	SMD 0.73 (-1.63, 3.10)	2	SMD -0.10 (-1.44, 1.24)	<0.001

CI, confidence interval; MD, mean difference; PPD, periodontal probing depth; P_{SG}, P value for differences between subgroups; SMD, standardized mean difference.
